# The Pharmacological Activities of *Crocus sativus* L.: A Review Based on the Mechanisms and Therapeutic Opportunities of its Phytoconstituents

**DOI:** 10.1155/2022/8214821

**Published:** 2022-02-14

**Authors:** Monica Butnariu, Cristina Quispe, Jesús Herrera-Bravo, Javad Sharifi-Rad, Laxman Singh, Nora M. Aborehab, Abdelhakim Bouyahya, Alessandro Venditti, Surjit Sen, Krishnendu Acharya, Moein Bashiry, Shahira M. Ezzat, William N. Setzer, Miquel Martorell, Ksenija S. Mileski, Iulia-Cristina Bagiu, Anca Oana Docea, Daniela Calina, William C. Cho

**Affiliations:** ^1^Banat's University of Agricultural Sciences and Veterinary Medicine “King Michael I of Romania” from Timisoara, Timișoara, Romania; ^2^Facultad de Ciencias de la Salud, Universidad Arturo Prat, Avda Arturo Prat 2120, Iquique 1110939, Chile; ^3^Departamento de Ciencias Básicas, Facultad de Ciencias, Universidad Santo Tomas, Chile; ^4^Center of Molecular Biology and Pharmacogenetics, Scientific and Technological Bioresource Nucleus, Universidad de La Frontera, Temuco 4811230, Chile; ^5^Facultad de Medicina, Universidad del Azuay, Cuenca, Ecuador; ^6^G.B. Pant National Institute of Himalayan Environment & Sustainable Development Kosi-Katarmal, Almora, Uttarakhand, India; ^7^Biochemistry Department, Faculty of Pharmacy, October University for Modern Sciences and Arts (MSA), 6th of October 12566, Egypt; ^8^Laboratory of Human Pathologies Biology, Department of Biology, Faculty of Sciences and Genomic Center of Human Pathologies, Faculty of Medicine and Pharmacy, Mohammed V University of Rabat, Morocco; ^9^Dipartimento di Chimica, “Sapienza” Università di Roma, Piazzale Aldo Moro 5, 00185 Rome, Italy; ^10^Molecular and Applied Mycology and Plant Pathology Laboratory, Department of Botany, University of Calcutta, Kolkata 700019, India; ^11^Department of Botany, Fakir Chand College, Diamond Harbour, West Bengal 743331, India; ^12^Department of Food Science and Technology, Nutrition and Food Sciences Faculty, Kermanshah University of Medical Sciences, Kermanshah, Iran; ^13^Pharmacognosy Department, Faculty of Pharmacy, Cairo University, Kasr El-Ainy Street, Cairo 11562, Egypt; ^14^Pharmacognosy Department, Faculty of Pharmacy, October University for Modern Sciences and Arts (MSA), 6th of October 12566, Egypt; ^15^Department of Chemistry, University of Alabama in Huntsville, Huntsville, AL 35899, USA; ^16^Department of Nutrition and Dietetics, Faculty of Pharmacy, University of Concepcion, Concepcion, Chile; ^17^Department of Morphology and Systematic of Plants, Faculty of Biology, University of Belgrade, Studentski Trg 16, 11000 Belgrade, Serbia; ^18^Victor Babes University of Medicine and Pharmacy of Timisoara Discipline of Microbiology, Timișoara, Romania; ^19^Multidisciplinary Research Center on Antimicrobial Resistance, Timișoara, Romania; ^20^Department of Toxicology, University of Medicine and Pharmacy of Craiova, 200349 Craiova, Romania; ^21^Department of Clinical Pharmacy, University of Medicine and Pharmacy of Craiova, 200349 Craiova, Romania; ^22^Department of Clinical Oncology, Queen Elizabeth Hospital, Kowloon, Hong Kong

## Abstract

*Crocus* species are mainly distributed in North Africa, Southern and Central Europe, and Western Asia, used in gardens and parks as ornamental plants, while *Crocus sativus* L. (saffron) is the only species that is cultivated for edible purpose. The use of saffron is very ancient; besides the use as a spice, saffron has long been known also for its medical and coloring qualities. Due to its distinctive flavor and color, it is used as a spice, which imparts food preservative activity owing to its antimicrobial and antioxidant activity. This updated review discusses the biological properties of *Crocus sativus* L. and its phytoconstituents, their pharmacological activities, signaling pathways, and molecular targets, therefore highlighting it as a potential herbal medicine. Clinical studies regarding its pharmacologic potential in clinical therapeutics and toxicity studies were also reviewed. For this updated review, a search was performed in the PubMed, Science, and Google Scholar databases using keywords related to *Crocus sativus* L. and the biological properties of its phytoconstituents. From this search, only the relevant works were selected. The phytochemistry of the most important bioactive compounds in *Crocus sativus* L. such as crocin, crocetin, picrocrocin, and safranal and also dozens of other compounds was studied and identified by various physicochemical methods. Isolated compounds and various extracts have proven their pharmacological efficacy at the molecular level and signaling pathways both *in vitro* and *in vivo*. In addition, toxicity studies and clinical trials were analyzed. The research results highlighted the various pharmacological potentials such as antimicrobial, antioxidant, cytotoxic, cardioprotective, neuroprotective, antidepressant, hypolipidemic, and antihyperglycemic properties and protector of retinal lesions. Due to its antioxidant and antimicrobial properties, saffron has proven effective as a natural food preservative. Starting from the traditional uses for the treatment of several diseases, the bioactive compounds of *Crocus sativus* L. have proven their effectiveness in modern pharmacological research. However, pharmacological studies are needed in the future to identify new mechanisms of action, pharmacokinetic studies, new pharmaceutical formulations for target transport, and possible interaction with allopathic drugs.

## 1. Introduction

The genus *Crocus* is a member of the Iridaceae (subfamily Crocoideae) and consists of about 100 species that occur in the wild. These are mainly found in central-southern Europe (Balkan Peninsula), North Africa, and Western Asia [1]. Several species of this genus are currently used in gardens and parks as ornamental plants for their colorful flowers, while *Crocus sativus* L. (saffron) is the only species that is cultivated for the edible purpose [2]. The use of saffron is very ancient: its earliest representation appeared approximately 4000 years ago in some paintings and ceramics of the Minoan civilization in the region of Crete [3, 4]. The stigmas from flowers are traditionally handpicked at dawn to preserve all the aroma and organoleptic characteristics. Then the stigmas are dried in the shade and finally powdered [5]. Due to its distinctive flavor and yellow-orange color, it has an ancient use as spice in Arab, European, Indian, and Persian cuisine. It is also used in liquors, candies, food supplements [5, 6], and medical and coloring qualities [7–9].

**Figure 1:**
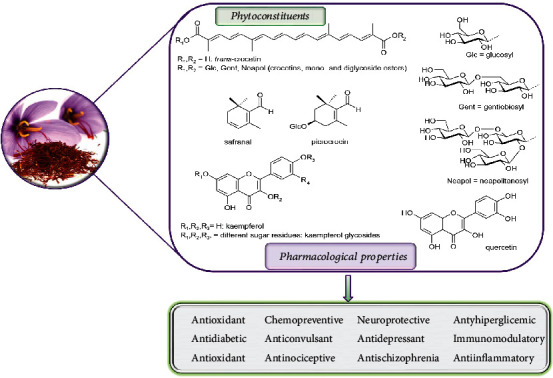
The main phytoconstituents of *Crocus sativus* L. and their pharmacological properties.

Among the phytoconstituents, there have been described several compounds that are thought to be the chemical determinants of the bitterness, scent, and color of saffron [10]. These are formally terpenoids or thought to be derived from terpenoid precursors. In particular, these are the apocarotenoids such as *trans*-crocetin and its glycosylated forms (crocins), especially *trans-*crocetin di-(*β*-D-gentiobiosyl) ester and *trans-*crocetin (*β*-D-gentiobiosyl)-(*β*-D-glucosyl) ester, together with picrocrocin and safranal (Figure 1), formerly a monoterpene glycoside and a monoterpene, respectively, and believed to be degradation products of zeaxanthin. As minor components, which also contribute to the spice color and biological effects, there are a series of glycosidic derivatives of kaempferol (Figure 1) [11] and quercetin [12]. Other minor components that contribute to the peculiar aroma of saffron are volatile compounds related to isophorone [13, 14].

Obviously, several of the medicinal and health-promoting properties attributed to saffron are due to the presence of these compounds. For example, safranal, in addition to its antioxidant and radical-scavenger properties, is useful as an anticonvulsant and antidepressant in preclinical models [15–18]. Due to the high added value of saffron as a spice and of its chemical constituents mainly related to the healthy properties, the studies on saffron and its plant source (*C. sativus*) are in the limelight, especially concerning the analytical methods used to evaluate the quality and to identify marker compounds related to the country of origin.

In this updated review, the following aspects of *Crocus* plants are considered: traditional uses, phytochemical composition, pharmacological properties with mechanisms evidenced from *in vitro* and *in vivo* studies, clinical studies, toxicological data, and upcoming clinical perspectives.

## 2. Methodology

An extensive search of the PubMed, Science, and Google Scholar databases was conducted to select the most important information for this updated review. The main search keywords were: “*Crocus* plant,” “*Crocus sativus*,” “ethnopharmacology,” “phytoconstituents,” “chemical compounds,” “pharmacological activity,” “pharmacological mechanisms,” “human clinical studies,” “toxicity,” and “food preservatives.”

Inclusion criteria are as follows: only extenso articles written in English that included data on *Crocus sativus* L. analyzed, chemical compounds isolated from each genus, pharmacological molecular research for each of the studied plants, types of *in vitro*/*in vivo* pharmacological studies, concentration and dose at which the chemical compounds studied were pharmacologically active, clinical trials, and toxicological data. The chemical names were validated with PubChem and SciFinder, and the plant names were in accordance with The Plant List [19, 20]. The most important data have been summarized in the tables and figures presented in this article.

Exclusion criteria are as follows: abstracts, letters to the editor, papers in languages other than English, studies that did not have dose-effect correlations, studies that did not have proven pharmacological activity, and studies that included homeopathic preparations.

## 3. Traditional Medicine Use of *Crocus* Plants

The folklore knowledge and extensive traditional use of medicinal *Crocus sativus* L. have a long history worldwide [21, 22]. The cultivation of *Crocus* species for culinary and therapeutic purposes has been known from ancient times, first taking place in Greece and Iran. Later, the utilization of these plants was spread through the Mediterranean region to Eastern Europe and South Asia and China [1]. Although *C. sativus* was the most popular medicinal plant in European and Asian countries for ages, among medically significant herbs from this genus are also *Crocus caspius* Fisch. & C.A.Mey. ex Hohen, *Crocus heuffelianus* Herb., *Crocus hyemalis* Boiss. & Blanche, and *Crocus vernus* (L.) Hill. Their ethnobotanical importance is reflected through traditional use against various ailments and health disorders [23–27].

The traditional application of *C*. *sativus* is very diverse from diet and cosmetics to essential roles in medicine. It is a reputable plant owing to the specific aroma, flavor, and color of the stiles. As a valuable medicinal plant, it is a part of many alternative therapies and folklore medicines [4, 22]. It is documented that saffron was applied for the treatment of about 90 health disorders by different cultures [28].

In some Asian countries, traditional knowledge on saffron healing effects dates from prehistoric periods [8, 29]. The first records about its medical application date from the 12th century BC [30]. Saffron was recognized by different nations in the Middle East and South Asia as a health-promoting drug and medicine to fight various diseases. It is believed that the people of ancient Persia were the first to cultivate this species for their traditional needs. In Persian traditional medicine, saffron was used as a tonic for the strength and meaning of the organism, especially the vascular and nervous system (NS). Additionally, it is an ingredient of recipes for the treatment of depression, insomnia, measles, and dysentery [8]. Concerning this, *C*. *sativus* remains one of the most important medicinal drugs in traditional Iranian medicine [4, 30].

It was an important curative herb in ancient Greece, Rome, and Egypt, South and Central Asia (Unani), Hindu (Ayurveda), and Chinese medicine [21, 31, 32]. Also, *C. sativus* has been utilized as a medicinal spice by the Moroccan people [30] and some locals in Jordan [23].

The ancient Romans added this plant to wines to prevent hangovers and intoxication. Also, it was used in ancient Egypt and Greece as a herbal remedy to fight ulcers on the skin or mucous membranes to reduce eye health problems such as pain, infection, or cataracts and to heal some urinary and menstrual disorders [30]. In Unani medicine, this spice is also recognized as a remedy for urinary and kidney infections, and it is used as a part of the mixtures against menstrual disorders. Besides, it is considered stomachic, while in combination with honey, it is useful as a diuretic [31].

In Ayurveda medicine, saffron has many purposes as well. It is recommended against skin problems, asthma, arthritis, and kidney and digestive disorders. In powdered form, the herb is useful for external application on wounds, swellings, or major bleedings [30]. Powdered saffron is also advised for the treatment of poor vision, cataracts, and blindness [8]. This herb acts against diabetes when combined with ghee. Among its most famous roles in Ayurveda, the system is in the reduction of the enlargement of the spleen and the liver. [21, 28, 31].

In general, *C*. *sativus* is advised as a strengthening tonic that stimulates the immune defense, and it is a common ingredient of restorative preparations recommended for the general improvement of the physical and mental health of the body [30]. As an antiseptic and anti-inflammatory drug, it heals bacterial and fungal infections, inflammations, insect bites, and also apoplexy, alcoholism, arthritis, and diabetes [30].

Saffron's stigmas are extensively applied as an indigenous medicine across India. It serves as an antiseptic, analgesic, and expectorant agent as a nerve sedative and stimulator for immunity, blood flow, and menstruation. Also, it is effective against smallpox and a wide range of stomach problems. In lower doses, this spice can stimulate the contraction of the uterus in pregnancy, while in more significant amounts, it can cause general spasm and constriction [8]. It is a popular natural cosmetic in Iranian and Indian medicine for improvement of skin complexion, and as a part of folk phytopreparation, it brightens the skin of the body [33].

In the cosmetic practice, this spice showed to be effective against acne, erysipelas, skin, wounds, and similar skin diseases, while in China, it is recommended for purpura, eczema, and measles [8, 30].

The aqueous extract of saffron, in combination with other medicinal drugs, is useful for the treatment of painful and dislocated joints, sprains, or fractured bones [29, 34]. Some nations use saffron to treat migraines or mental disorders such as depression and dementia. For example, in Iran, insomnia, severe headaches, and obstructions in the area of the head and the neck are cured with boiled water preparation of saffron, while in India, it is applied against depression and similar mental disorders [1, 30]. *Crocus sativus* acts as an improving mood and calming anxiety agent [29]. The essential oil of saffron can be used as a sedative in combination with olive or sesame oil (as a medicine, the mixture of oils is left for five days with periodically stirring then filtered afterwards) [30]. The flowers of *C. sativus* are used as a part of alternative therapy for Alzheimer's disease [26].

Saffron is regarded as a stomachic and carminative drug, helpful for the liver, spleen, and stomach irregularities [29]. Also, it demonstrates emetic activity and can relieve problems with dyspepsia and to decrease appetite [30]. It helps with obesity-related metabolic disorders like hyperlipidemia and diabetes. Regarding this, the role of saffron in action against obesity was later confirmed [34]. Considering the cardiovascular system, *C*. *sativus* is used for many purposes. As cardiotonic, it affects the heart and speeds the circulation. As a result of better blood flow, other medicaments reach the targeted organs more quickly. Saffron is used in England with the purpose to strengthen and stimulate the heart by better nutrition caused by faster blood flow. Besides these functions, the drug is applied in China against anemia and to prevent coagulation by breaking the blood clots [29, 34]. In both Western and Eastern parts of the world, it is used as a medicine for problems with the respiratory system [30]. The saffron essential oil quickens the lung's function, relaxes the breathing, and is recognized as an expectorant. It is known to combat coughs and colds, partly by its specific odor, as well as asthma, pleurisy, and diaphragmatic [32]. Additionally, the plant helps with urinary infections. Traditionally, it is used as a herbal antispasmodic remedy that prevents obstructed urination.

The diuretic combination of saffron and honey helps to release kidney stones [30]. *Crocus sativus* is a folk medicament advised for irregularities in the menstrual cycle, which expresses many beneficial roles in the female reproductive system. It alleviates dysmenorrhea and has an emmenagogue application. Saffron relieves the pain in the uterus when combined with other drugs. Also, it can be used against uterus ulcers when applied with wax or yolk and olive oil. *Crocus sativus* is an aphrodisiac drug recognized for treatment of impotence. It has a traditional role in sperm activation. As already mentioned, it demonstrates abortive activity in larger amounts which can cause the uterus spasm and abortion in the pregnancy. Also, it is popularly used as a postlabour antiseptic in cows [29, 30, 35].

According to literature data, other *Crocus* species are also utilized as natural phytotherapeutics. It is claimed in Iranian natural medicine that the endemic species *C. caspius* is medicinally effective against microbial infections [24]. The infusion of *C. hyemalis* aerial parts and stigma's filaments is recommended as an antitussive and antiasthmatic remedy and as a medicine for respiratory problems [23]. Furthermore, the significance of *C. vernus* in mountainous areas of Italy is expressed through the use of its flowers or entire flowering plant as an antiseptic, while the flower buds are advised externally against lice [27]. It was cited in traditional books that the mixture of *C. sativus*, *Cyperus rotundus* L., honey, and currant refreshes the memory and reduces forgetfulness and distraction [26].

Many beneficial properties of *Crocus* species are mainly related to a variety of carotenoids and their crocetin-type derivatives [1, 27]. The most important findings on the health-promoting effects of saffron are confirmed for cardiovascular ailments like blood pressure (BP), atherosclerosis, and coronary artery diseases. Furthermore, studies have confirmed that it impacts ocular blood flow, retinal function, and learning behavior, and it demonstrates anti-inflammatory, cytotoxic, and contraceptive activities [31].

## 4. Phytochemical Composition

Because of its wide range of pharmacological uses, saffron has undergone extensive biochemical and phytochemical studies (Supplementary material (available here), Tables 1 and 2), and several biologically active compounds have been isolated. Each of these bioactive compounds has characteristic inherent properties (Figure 1).

Saffron showed antimicrobial effects and can protect foods from microbial attack. Saffron flower petals are rich in phenolic compounds showing antimicrobial and antioxidant activity. Crocin is the most powerful antibacterial agent in saffron, particularly against Gram-negative bacteria like *Escherichia coli*. The high antibacterial activity of crocin is attributed to the alcoholic groups (-OH) in its structure.

The peculiar quality and sensory properties in terms of aroma and taste have been attributed to three main bioactive compounds, crocin, picrocrocin, and safranal [36–38]. It also contains many nonvolatile components like lycopene, *α*- and *β*-carotene, and zeaxanthin [39].

The crocins, hydrophilic compounds, are considered the main active constituents that give saffron the golden yellow-orange color [40–42]. Picrocrocin is responsible for the bitter taste (the bitter glucoside) [43, 44]. Safranal, a monoterpene aldehyde, produces the characteristic odor and aroma. [45–47].

These bioactive compounds are the degradation products of carotenoids, i.e., derived from oxidative cleavage zeaxanthin carotenoid, and are known as apocarotenoids [48, 49]. Additional bioactive compounds that are present include carbohydrates, flavonoids, gum, minerals, proteins, sugars, and vitamins, which have been isolated and reported from the saffron stigma [47, 50, 51].

## 5. Pharmacological Activities

Traditional and alternative medicine has a significant impact on the current trends in preclinical and clinical research of medicinal plants [52–54]. Since saffron is a popular folk remedy in many countries, recent preclinical studies verified its wide application and the use of related *Crocus* species for medical purposes.

### 5.1. *In Vitro* studies

#### 5.1.1. Cytotoxic Activity

Cancer is a term used to describe malignancies in which abnormal cells multiply in an uncontrolled and continuous manner and can invade the surrounding healthy tissues [53–55]. Abnormal cells come from any tissue in the human body and can occur anywhere in the body [56, 57]. The malignancies caused by this uncontrolled multiplication are numerous and difficult to control therapeutically [58, 59].

Antitumor effects of *Crocus* plant derivatives have been highlighted in a wide variety of isolated cell study models. The viability of healthy cells remained unaffected under treatment, while compared to malignant cells, including human cancer cells, saffron develops selective cytotoxic effects at micromolar doses.

Aqueous extract of saffron used in different concentrations (100, 200, 400, and 800 *μ*g/mL) exhibited cytotoxic and proapoptotic effects when investigated on lung cancer cell line A549. It was found that saffron reduced the proliferation of the A549 cells in a dose-dependent manner. In addition, it induced morphological changes, reduced the number of viable cells, and induced apoptosis. The IC_50_ against A549 cells was 380 and 170 *μ*g/mL after 48 and 72 hours, as reported by Samarghandian et al. [60].

Another study conducted by Vali et al. [61] on a breast cancer cell line (MCF-7), a synergistic effect was found between combinations of crocin with gamma radiation or paclitaxel in increasing apoptosis and decreasing survival rate of the cells. The MTT assay was used to determine the IC_50_ (3.5 mg/mL) of crocin after 48-hour treatment. Also, the treatment of MCF-7 with crocin at different time intervals increased apoptosis of the cells as detected by flow cytometry, where the combined therapy of crocin and paclitaxel increased apoptosis significantly over single therapy. On the other hand, the combined therapy caused an increase in the expression of caspase-7, caspase-9, P53, and poly (ADP-ribose) polymerase (PARP) [61].

Another approach was taken Mousavi et al. [62] on the MCF-7 cell line treated with aqueous saffron extract (100, 200, 400, and 800 *μ*g/mL) using the trypan blue assay to investigate the morphological changes of cells under an inverted microscope as well as gene expression of matrix metalloproteinase (MMP). The treatment groups showed a significant reduction in MMP gene levels compared to the control group. Since MMPs regulate the signaling pathway that controls cell growth, inflammation, or angiogenesis, MMPs may be a target in cancer treatment and metastasis inhibition.

An earlier study was performed in which HeLa cells were treated with an ethanolic extract of saffron and its compounds. The IC_50_ for the ethanolic extract was 2.3 mg/mL, 3 mM for crocin and picrocrocin, separately, and 0.8 mM for safranal. Crocin, safranal, and picrocrocin showed a dose-dependent inhibition of cell growth, while crocetin did not show any effect on cell proliferation [63].

Sun et al. [64] examined the effect of crocin on human promyelocytic leukaemia cells and HL-60 cells *in vitro* where they found that crocin (0.625-10 mg/mL) significantly inhibited HL-60 cell proliferation dose-dependently and induced cell cycle arrest at G0-G1 phase in HL-60 by flow cytometry using propidium iodide staining.

Another study was performed by Tuberoso et al. [65] in which cytotoxicity of saffron juice was evaluated by MTT assay on Caco-2 colon cancer cell line; the cell viability was 30% at 48 h treatment with saffron juice (10 *μ*L/mL), while at 24 h treatment, cell viability was 32% but only at a higher concentration (50 *μ*L/mL).

#### 5.1.2. Antimicrobial Activity

Bacterial infections can trigger various diseases such as pneumonia, otitis, diarrhea, and skin infections and are the result of severe infections with germs difficult to treat such as bacteria in the category of Gram-negative germs Enterobacteriaceae, Streptococci, *Escherichia coli*, or *Salmonella* [66, 67]. The main method of fighting bacterial infections is antibiotic treatment [68]. Although they are effective in most cases, their widespread use has led to antibiotic resistance. This phenomenon consists in the adaptation of bacteria and, consequently, to difficulties in treating infections. Due to the use of antibiotics, the saprophytic microbial flora is destroyed, the flora that facilitates digestion and supports the immune system. Thus, side effects such as gastrointestinal disorders, diarrhea, and allergic reactions occur. Therefore, alternative natural treatments with proven antibacterial effects are an important antimicrobial alternative.

Antimicrobial activity of *C. sativus* extracts (500, 750, and 1,000 *μ*g/disc) was evaluated by the presence or absence of inhibition zone and zone diameter [69]. Maximum zone inhibition of the petroleum ether extract was shown against *Proteus vulgaris*, *Bacillus subtilis*, and *Pseudomonas aeruginosa*, whereas the methanolic extract showed maximum zone inhibition against *S. aureus* and *E. coli*, as demonstrated by Muzaffar et al. [69].

Kakouri et al. [70] investigated the antimicrobial activity of two extracts of *C. sativus* tepals. One extract contained the aglycon part of flavonoids and the other contained flavonoids glycosides. The antimicrobial activity of the extracts was evaluated against six bacterial species by well diffusion assay, where the extract that contained the glycosidic part of flavonoids exhibited weak antimicrobial activity. The best antimicrobial capacity was presented by tepal extract containing aglycons. Results from a study conducted by Hussein et al. [71] indicated strong antibacterial activity of the saffron extract against *E. coli* and *Staphylococcus aureus* at a concentration of 100 *μ*g/mL. They claimed that the methanolic extract of crocin possesses the highest antibacterial activity against *E. coli* and *S. aureus* compared to other saffron pigments. They found that the antibacterial effect of crocin is approximately equal to chloramphenicol and ciprofloxacin (known as standard antibiotics) at 100 *μ*g/mL concentration [71].

Another study was performed to assess the antimicrobial activity of aqueous extract of *C. sativus* collected from different areas in Iran using 3 different bacteria by a modified well plate test where different concentrations of extract were done and evaluated based on growth inhibition zone in which some of them had antimicrobial (*S. aureus* and *E. faecalis*) activity while others had no detectable activity (*E. coli*) [72].

#### 5.1.3. Antioxidant Activity

**Figure 2:**
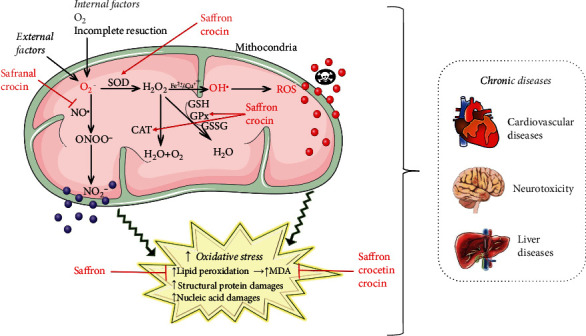
Illustrative scheme with different pathways of ROS formation and their impact on health. Bioactive compounds of *Crocus sativus* L. interfere with these mechanisms showing the beneficial effects for human health. Abbreviations and symbols: ↑ (increase, stimulate), ꓕ (decrease, inhibition), CAT (catalase), NO (nitric oxide), MDA (malondialdehyde), ROS (reactive oxidative species), glutathione peroxidase (GPx), GSSG (oxidized glutathione), GSH (reduced glutathione), O_2_^−^ (superoxide), H_2_O_2_ (hydrogen peroxide), OH^●^ (hydroxyl ions), and NO (nitric oxide).

Oxidative stress is the term used for diseases caused by reactive oxygen species (ROS) called free radicals [73, 74] and is defined as the imbalance between oxidants and antioxidants, in favor of oxidants, with destructive and pathogenetic potential [75, 76]. Depending on the intensity, oxidative stress can occur intra- or extracellularly [77]. Intracellular oxidative stress can cause cell necrosis or more or less marked cell disorganization, with catastrophic effects in the case of a cell that cannot reproduce. Extracellular oxidative stress is also cytotoxic [78, 79] (Figure 2). Among the saffron constituents, crocetin has stronger antioxidant activity (DPPH (2,2-diphenyl-1-picryl-hydrazyl-hydrate)) than safranal, and the potential of crocetin was equivalent to that of Trolox and butyl hydroxyl toluene (BHT) [80].

Crocin, ethanolic extract of saffron, and different extracts of *C. chrysanthus* were evaluated by different *in vitro* assays: antihemolysis, DPPH free radical-scavenging assay, *in vitro* lipid peroxidation, ferric reducing antioxidant power, phosphomolybdenum method, metal chelating, and reductive potentials [81–83]. The studied extracts and isolates from saffron exhibited significant antioxidant activity (Table 3); however, this activity is affected by the type of solvent used that is why they may be involved in the treatment of various diseases *via* free radical scavengers.

Another study was done to evaluate the antioxidant activity of corms, tepals, and leaves of saffron to increase the profitability of this crop in which the leaf extract showed the best antioxidant activity *via* total inhibition of the *β*-carotene oxidation at 10 *μ*g/mL and a DPPH scavenger activity higher (up to 32 times) than those reported for traditional sources of antioxidants; a similar effect was shown with tepal extract, but in contrast, corm extract was a weak antioxidant [84].

Crocin had the stronger antioxidant capacity on rat pheochromocytoma (PC-12) cells than the standard antioxidant agent *α*-tocopherol so that it could reverse the results of the cell membrane damage and enhanced superoxide dismutase (SOD) level in oxidatively stressed neurons [85]. Bukhari et al. [86] investigated in stressed bronchial epithelial cells by cytokine combination the effects of *C. sativus*. Saffron treatment and its constituents (safranal and crocin) decreased nitric oxide of (NO), induced nitric oxide synthase (iNOS) levels, and peroxynitrite ion generation and prevented cytochrome c release.

Saffron constituents reduced lipid peroxidation [87] and prevented the increase of oxidative stress markers induced by diazinon through free radical-scavenging activity [88]. Moreover, a mitochondrial and lipid peroxidation protection against 3-nitropropionic acid toxin has also been reported in striatal synaptosomes isolated from rat brain [89].

A summarized scheme with the most representative antioxidant mechanisms of phytocompounds of *Crocus sativus* L. is shown in Figure 2.

### 5.2. *In Vivo* studies, cellular, and molecular pharmacology

#### 5.2.1. Effects on Cardiovascular System

*(1) Antihypertensive Effect*. High BP is a major cardiovascular risk factor with a growing incidence [75]. High BP can be controlled with medication, but natural remedies with proven antihypertensive effects are also used as adjuvant therapy [91].

*Crocus sativus* extract and safranal were reported to stimulate *β*2-adrenoreceptors [92, 93]. Also, safranal can act as a muscarinic receptor blocker, and *C. sativus* has an inhibitory or even antagonistic effect on histamine (H1) receptors [93].

Crocetin has been reported to lower the BP [94]. Yoshino et al. [95] observed the antioxidant potential of crocetin in stroke-prone spontaneously hypertensive rats and a significant inhibition of hydroxyl radical generation.

Crocin and safranal have BP-modulating features, but the mechanism of action is still under investigation [96]. Crocin and safranal showed a hypotensive effect in a dose-dependent manner, being safranal more potent [97].

In addition, the cardioprotective role of saffron and its constituents has been reported [98]. The aqueous extract of saffron stigmas was reported to have an antihypertensive and normalizing effect on the BP of normotensive and desoxycorticosterone acetate (DOCA) salt-induced hypertensive rats [99]. Aqueous extract of saffron petals (500 mg/kg) reduced BP through its direct effect on the heart itself or the total peripheral resistance or both [100]. In rats' isolated vas deferens, contractile responses to electrical field stimulation were decreased by the petal extracts [100]; this effect was mediated by cotransmitter noradrenaline and ATP released from sympathetic nerves. Another study suggested that saffron exerted a significant cardioprotective effect by preserving hemodynamics and left ventricular functions [101].

*(2) Antiarrhythmic effect*. Saffron plays an important role in the electrophysiological remodeling of the atrioventricular (AV) node during atrial fibrillation [102]. Boskabady et al. [103] observed a potent inhibitory effect of saffron aqueous extract on the noradrenaline of the guinea pig's isolated heart. In patients with ischemic heart disease, crocin also can be utilized for the prevention or treatment of arrhythmias [104]. Crocin was tested against cardiac reperfusion-induced arrhythmia, where it showed a defensive role in cardiac reperfusion arrhythmias, through amplification of antioxidant systems [104].

Myocardial damage and arrhythmia are associated with increased malondialdehyde (MDA) level, decreased activity of antioxidant enzymes, accumulation of free radicals, and the effect on ion Ca^2+^ channels [105]. Inhibition of ADP and collagen-induced platelet aggregation by crocetin *via* inhibition of Ca^2+^ elevation in stimulated platelets have also been reported in a dose-dependent manner [106]. The suggested cardioprotective effect of saffron mechanism is antioxidant activity, recovery, and upregulation of antioxidant enzymes [107], e.g., glutathione peroxidase (GPx), by inhibition of cardiac calcium channels.

*(3) Effect on Myocardial Ischemia*. In isoproterenol- (ISO-) induced myocardial infarction rat model, Goyal et al. [108] observed a dose-dependent preventive effect of saffron through histopathological and ultrastructural examinations. In addition, intravenous crocin reduced myocardial injury and lactate dehydrogenase (LDH) and creatine kinase (CK) level [109]. Orally administration did not show the same effects, possibly because of the inefficient absorption.

*(4) Antiatherosclerotic Effect in Cardiovascular Diseases*. In bovine aortic endothelial cells, crocin regulated redox status in a dose-dependent pattern and exhibits regression and inhibition of atherosclerosis *via* apoptosis by increasing Bcl2/Bax ratio expression [109]. Antiatherosclerotic effects of saffron were observed mainly because of crocetin that decreased the level of cardiac markers, e.g., LDH, CK, and MDA, besides increasing the mitochondrial potential in noradrenaline-treated cardiac myocytes [110].

Crocetin administration significantly decreased total cholesterol (TC) deposits in aorta, atheroma, foam cells, and atherosclerotic lesions in the crocetin fed animals [110]. A possible mechanism involved is due to suppression of nuclear factor- (NF-) *κ*B, which in turn decreases the vascular cell adhesion molecule-1 (VCAM-1) expression [111]. This antiatherosclerotic effect of crocetin has been also attributed to its antioxidant activity that decreases ROS-induced MDA levels [101]. In another study, crocetin decreased the TC level in the blood and thus reduced the risk of atherosclerosis and heart attacks. This effect may be due to the reinforcement of blood circulation [112]. Hemmati et al. [113] compared the antiatherogenic effects of three medicinal plants *C. sativus*, *Beta vulgaris* L., and *Ziziphus jujuba* Mill. in diabetic rat models, where the three extracts possessed antiatherogenic activity, which is probably associated with the antioxidant capacities of the extracts.

#### 5.2.2. Antiproliferative and Cytotoxic Activities

Saffron and its carotenoid constituents are chemopreventive in the growth of human malignant cells and animal models. Chermahini et al. [114] reported that saffron and its constituents could inhibit the synthesis of cellular DNA and RNA with no effect on protein synthesis in tumor cells. The antitumor effect of saffron and its ingredients is due to the free radical-scavenging effect, together with the interaction with topoisomerase II [114, 115].

Saffron exerted a protective effect against the toxicity of cisplatin when applied with the cysteine and vitamin E [116, 117]. Saffron can potentiate the effect of other anticancer agents through the inhibition of colony formation and nucleic acid synthesis [116]. Saffron aqueous extract also reduced the dimethylnitrosamine- (DEN-) induced hepatic cancer through induction of apoptosis, inhibition of cell proliferation, oxidative stress, and inflammation [118]. Premkumar et al. [119] showed the antimutagenic and antioxidant potential of aqueous extract of saffron.

In mice, an aqueous extract of saffron has been found to prevent specific drugs (cisplatin, urethane, cyclophosphamide, and mitomycin C) that induced genotoxicity and oxidative stress besides increasing hepatic enzymes such as SOD, catalase (CAT), and nonenzymatic antioxidants [120]. The authors suggest that its chemopreventive role is observed because of its antioxidant activity and modulatory property during lipid peroxidation and detoxification.

#### 5.2.3. Neuroprotective Effects

*(1) Anticonvulsant Activity*. In pentylenetetrazole- (PTZ-) and maximal electroshock seizure- (MES-) induced seizures in mice, Hosseinzadeh and Khosravan [121] indicated an anticonvulsant activity of aqueous and ethanolic extracts of *C. sativus*. Similar anticonvulsant activity was shown by safranal, contrary to that of crocin, which did not show any effect [16]. It is suggested that the anticonvulsant effect of safranal is mediated partly through GABA (A)-benzodiazepine receptor complex [122–126]. In addition, it is assumed that saffron's anticonvulsant and analgesic properties and its effects on morphine withdrawal might be due to an interaction between saffron, GABA, and opioid system [127]. Saffron did not significantly suppress PTZ-induced seizures at a dose of 200 mg/kg in rats [128].

*(2) Neuroprotection in Neurodegenerative Diseases*. Neurodegenerative diseases, such as Alzheimer's and Parkinson's diseases, are characterized by the presence of protein aggregates, inflammation, and oxidative stress in the central nervous system (CNS) [129]. A number of factors are involved in the onset of neurodegenerative diseases, which lead to the gradual deterioration of the health of the nervous system, with serious consequences on the quality of life of the patient with such a disease [130]. Although there are still no treatment solutions to restore nerve function in neurodegenerative diseases, more and more studies insist on several natural formulas that have been shown to have the effect of reducing symptoms and improving the quality of life of patients with neurodegenerative diseases. Alzheimer's disease is a neurodegenerative disease that causes disorders of memory, thinking, and behavior [131].

Saffron has been reported to inhibit the aggregation and deposition of amyloid *β* (A*β*) and thus prevent the short-term memory problems characteristic of mild to moderate Alzheimer's disease. Inhibition of A*β* fibrillogenesis by methanol and water extract of *C. sativus* stigmas is dose- and time-dependent [132–134]. Crocin was found more effective in preventing the toxic amyloid structures accumulation due to its amphiphilic properties [132]. On the other hand, *trans*-crocin 4 was more effective in Alzheimer's disease than dimethyl crocetin in inhibiting A*β* fibrillogenesis through oxidation of the amyloid *β*-peptide fibrils [135]. Treatment with saffron extract could improve cognitive deficits induced by intracerebroventricular (ICV) injection of STZ in rats [136]. However, Khalili and Hamzeh [137] reported that the main component of saffron, crocin, is responsible for antagonizing the cognitive deficits caused by STZ-ICV in rats and can be used for treating the neurodegenerative diseases. Saffron had shown about 30% inhibitory effect on acetylcholinesterase (AChE) activity, which can be another mechanism for treating Alzheimer's disease [138, 139].

Parkinson's disease is related to dopamine deficiency due to genetic factors or Pb intoxication and is characterized by the degeneration of neurons in the substantia nigra [140]. The accumulation of lead (Pb) in the environment causes intoxication of the body, mainly affecting the CNS as it leads to structural and functional disruption of the CNS, and it may also develop Parkinson's disease. In a study performed by Tamegart et al. [141], the intraperitoneal injection of Pb caused a neurotoxic effect on the dopaminergic system and locomotor performance in Meriones shawi rats. The oral gavage of *C. sativus* (50 mg/kg body weight) prevented Pb-induced damages. Polyphenols such as quercetin and catechin have demonstrated Fe and Zn chelation activities. Thus, saffron may have a neuroprotective activity for neurodegenerative disorders, implying dopaminergic and noradrenergic injuries, especially heavy metal-induced Parkinson's disease.

Oxidative stress in the CNS is related with neurodegenerative diseases [142–144]. Crocetin could strengthen the antioxidant system and reduce thiobarbituric acid (TBARS), therefore inhibiting the effect of 6-hydroxydopamine, which is involved in inducing Parkinson's disease, and also decreased the utilization of dopamine [145]. In mice, saffron also showed effectiveness against MPTP- (1-methyl-4-phenyl-1,2,3,6-tetrahydropyridine-) induced Parkinson's disease, as pretreatment with saffron protected the dopaminergic cells in the substantia nigra pars compacta and retina [146].

*(3) Antidepressant Effect*. Saffron and its components possess antidepressant and anxiolytic effects [132]. Crocin (50–600 mg/kg) reduced immobility time in rats in the forced swimming test, with the increase in climbing time [18]. Wang et al. [147] demonstrated that the petroleum ether and dichloromethane fractions *C. cerebral sativus* L. corms have an antidepressant effect.

The aqueous and ethanolic extracts of *C. sativus* petal and stigma [148], as well as safranal and crocin, had shown antidepressant activity in mice [18]. In addition, kaempferol, a constituent of *C. sativus* petals, also reduced immobility behaviors in mice at 100 and 200 mg/kg and rats at a dose of 50 mg/kg [149]. The reduced time of immobility in rats and mice is usually due to the selective serotonin reuptake inhibitors such as fluoxetine, and this may be the mechanism by which *C. sativus* exerts its antidepressant effects [150].

*(4) The Effects on Neurotoxicity and Neuronal Oxidative Damages*. Neurotoxicity refers to disturbances or damage to the CNS by toxic substances and toxins that affect the nervous system are called neurotoxins [52, 151].

Safranal has neuroprotective effects on oxidative damage markers in hippocampal tissue in ischemic rats [152] and in hippocampal tissue in rats treated with quinolinic acid [153]. Safranal decreases extracellular content of the excitatory amino acids, glutamate, and aspartate in the hippocampus of anaesthetized rats treated with kainic acid [154].

Crocetin can inhibit early stages of apoptosis and induce angiogenesis at the subacute stage as depicted by vascular endothelial growth factor receptor-2 (VEGFR-2) and serum response factor (SRF) expression levels, so it exerts *in vivo* neuroprotective effects the brain [155]. It has been demonstrated that crocetin could potentiate the antioxidant capacity in the brain and prevents 6-hydroxydopamine-induced neurotoxicity [145].

Crocin has a unique, protective effect on ethanol-induced impairment of learning and memory [134].

Sahraeil et al. [156] reported that saffron ethanolic extract and crocin are effective against chronic stress-induced Wistar rats, through interaction with hormonal, metabolic, and behavioral changes induced by electric shock stress in rats. Saffron extract and crocin also improved spatial cognitive abilities following chronic cerebral hypoperfusion, most probably due to their antioxidant potential [157].

Crocin also potentiated SOD and GPx activity and decreased MDA concentration in the cortex of the ischemic stroke rat model [158]. In an ischemic stroke rat model, crocin increased antioxidant enzyme activity of SOD, CAT, and GPx and reduced MDA levels and lipid peroxidation [89]. In cerebral ischemia, crocin inhibited oxidizing reactions in mice microvessels in addition to modulating the ultrastructure of cortical microvascular endothelial cells (CMEC) [159]. Crocin and crocetin can inhibit the activated microglia by the repression of the NF-*κ*B transcriptional activity [160].

Both saffron extract and crocin may improve learning and memory [161, 162] as both can prevent oxidative stress in the hippocampus [138]. The enhancing effect of saffron on memory is mediated by its effect on the cholinergic system [162, 163].

Saffron and its derivatives act as curative agents in focal ischemia [164], autoimmune encephalomyelitis in C57BL/6 mice, cerebral ischemia [158], hippocampal ischemia [165], and renal ischemia/reperfusion [166]. In the whole brain and cerebellum, saffron extract reversed aluminum-induced changes in monoamine oxidase A and B activity and lipid peroxidation levels [167]. The antioxidant potential of saffron may be responsible for attenuation in cerebral ischemia-induced oxidative damage in the rat hippocampus [152]. Ghazavi et al. [123] investigated ethanolic extracts of saffron in mice and observed an increase of antioxidant potential, increased level of glutathione and its dependent enzyme, and a suppression of the increased levels of MDA, glutamate, and aspartate.

*(5) Effect on Brain Receptors*. Saffron was reported to have a similar effect to *N*-methyl-D-aspartate (NMDA) receptor antagonists on conditioning place preference induced by morphine [168]. Furthermore, saffron analgesic effect may be reduced by NMDA receptor antagonists, which suggested an interaction of saffron with the glutamatergic system [169].

Crocin (200 and 600 mg/kg) could inhibit the morphine withdrawal symptoms with no effect on the locomotor system [170, 171]. The saffron extract reduced morphine-induced memory impairment [125] and prevented morphine-induced inhibition of spatial learning and memory in rats [172].

#### 5.2.4. Effects on Metabolisms

*(1) Hypolipidemic Effect*. Premkumar et al. [173] showed that saffron and its constituents decreased triglycerides (TGs), TC, alkaline phosphatase (ALP), aspartate transaminase (AST), alanine aminotransferase (ALT), MDA, and GPx, reduced glutathione (GSH) and oxidized glutathione (GSSG) levels in serum, and provoked an increasing effect on SOD, CAT, fluorescence recovery after photobleaching (FRAP), and GSH values in the liver tissue. Saffron was more effective than its constituents to quench free radicals and ameliorate the damages of hyperlipidemia [174].

In diet-induced hyperlipidemic rats, crocin showed hypolipidemic effect by reducing serum TG, TC, and low-density lipoprotein (LDL), and very-low-density lipoprotein (VLDL) levels [175]. Hypoglyceridemic and hypocholesterolemic effects of crocin are also reported in quails kept on a hyperlipidemic diet [110, 176]. Crocin selectively inhibits pancreatic lipase through competitive inhibition and provokes lipid decrease [175].

In quails, the reduction of serum TC, LDL, and TG was also prominent in treatment with crocetin [110]. Cousins and Miller [177] reported that intraperitoneal injection of crocetin was more effective in showing hypolipidemic effect compared to that of the subcutaneous injection. In hypolipidemic rats, crocetin, along with crocin, showed an inhibitory effect on the increased serum TG, TC, and LDL levels [110, 176].

*(2) Antihyperglycemic Effect*. Diabetes is the most common disease of the endocrine system and is triggered when the amount of insulin secreted in the body is not optimal or when peripheral cells do not respond to its action (insulin is a hormone that lowers blood glucose) [178, 179].

*Crocus sativus* aqueous extract has also been reported to have an effect on streptozotocin- (STZ-) induced diabetic rats [180]. Diabetic rats treated with aqueous saffron extract showed reduced expression of inflammatory cytokines in the abdominal aorta. Thus, saffron can be useful also in treating diabetes mellitus and its vascular complications.

Saffron, crocin, and safranal have shown antihyperglycemic activity in the alloxan-diabetic rats through increasing blood insulin levels and caused the renewal of *β*-cells in alloxan-diabetic rats with neither liver nor kidney toxicities [181–183]. Crocetin was able to increase insulin sensitivity, improving impaired glucose tolerance, hypertension due to a high-fructose diet, and dexamethasone injection in rats [82, 175, 184]. Also, crocetin reduced the palmitate-induced insulin sensitivity in the rat adipocytes [185]. Crocetin could also prevent diabetes-related vascular complications [186, 187].

### 5.3. Other Pharmacological Activities

Saffron and safranal extract show preventive effects in lung pathology during lung inflammation of sensitized guinea pigs [188]. Safranal was shown to significantly reduce the cough count in citric acid aerosol-induced irritation in guinea pigs [189]. This effect may be due to competitive antagonistic activity to histamine H1 receptors [190]. Safranal was tested on the murine model of asthma, where it increased airway hyperresponsiveness and, in lungs, reduced iNOS production, bronchial epithelial cell apoptosis, and Th2-type cytokine production [86].

Saffron showed an important role as a curative agent in visual impairment due its antioxidant potential [191]. Saffron as a dietary supplement prevents the effects of continuous light exposure that may cause photoreceptor and retinal stress in albino rats, besides maintaining both morphology and function by acting as an apoptotic regulator [192, 193]. In ischemic retinopathy, crocin facilitates the recovery of retina functioning [194]. In murine retina, oral administration of crocetin prevents NMDA-induced retinal damage by inhibiting the caspase pathway [195], and *trans*-crocetin showed an antagonistic effect of *C. sativus* extract on NMDA receptors [196]. Crocin (50 mg/kg) inhibits retinal ganglion cell (RGC) apoptosis after retinal ischemia/reperfusion injury *via* phosphatidylinositol 3-kinase/AKT (PI3K/AKT) signaling pathway and increasing Bcl − 2/BAX ratio [197]. Crocin (10 *μ*M) could suppress tumor necrosis factor- (TNF-) *α*-induced expression of proapoptotic mRNA, which releases cytochrome c from mitochondria [198]. Moreover, crocetin can inhibit cell death of H_2_O_2_-induced RGC-5 and inhibit caspase-3 and caspase-9 activity [199].

Crocin analogues increased the blood flow in the retina and choroid and facilitated retinal function recovery [194].

Hosseinzadeh and Younesi [200] have shown that the ethanolic and aqueous extracts of saffron stigma could inhibit the acetic acid-induced writhing reflex *in vivo* and also had a curative effect on many complications such as the injury of the skeletal muscle of the lower limb [201] and reepithelialization of burn wounds [202].

**Figure 3:**
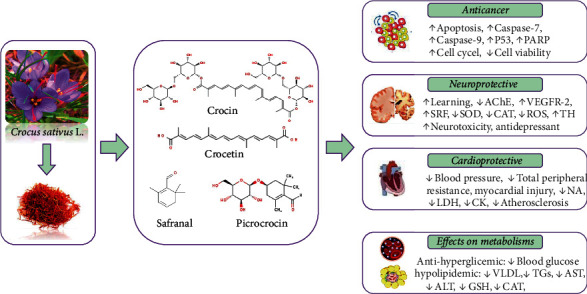
The role of *Crocus* plants' phytoconstituents in the pharmacotherapeutic management of various disorders and the possible molecular mechanisms of action. Abbreviations and symbols: ↑ increased, ↓ decreased, acetylcholinesterase (AChE), alkaline phosphatase (*ALP*), aspartate transaminase (*AST*), catalase (CAT), creatine kinase (CK), *glutathione* (*GSH*), lactate dehydrogenase (LDH), noradrenaline (NA), poly (ADP-ribose) polymerase (PARP), reactive oxygen species (ROS), serum response factor (SRF), superoxide dismutase (SOD), *tumor* protein *P53* (p53), tyrosine hydroxylase (TH), vascular endothelial growth factor receptor-2 (VEGFR-2), and very-low-density lipoprotein (VLDL).

The most relevant *in vivo* pharmacological studies with the major findings are shown in Table 2 and Figure 3.

## 6. Clinical Studies

Saffron extract (30 mg/kg for six weeks) had been reported to possess an antidepressant effect on patients similar to the effects of fluoxetine [215] and imipramine 100 mg/day [15] (Table 3). Saffron extract at this dose was equally effective to fluoxetine (40 mg/day) in improving depression symptoms in patients who were suffering from major depressive disorder (MDD) after undergoing percutaneous coronary intervention [216]. Basti et al. [217] also suggested its effectiveness in treating mild to moderate depression.

Lopresti et al. [218] designed a randomized, double-blind, placebo-controlled study for 8 weeks on patients with 12–16 years of age, with mild to moderate anxiety or depressive symptoms. Tablets containing saffron extract (Affron®, 14 mg b.i.d.) were used. The treatment improved anxiety and depressive symptoms in youth with mild to moderate symptoms, at least from the perspective of the adolescent. However, these beneficial effects were not corroborated by parents.

Administration of saffron, 30 mg/day, divided as 15 mg two times daily, in subjects of 55 years and more, was as effective as donepezil for the treatment of mild to moderate Alzheimer's disease [219]. The saffron extract had similar side effects to those of donepezil but with less vomiting [219]. Another study performed on 46 patients with mild to moderate Alzheimer's disease had shown that saffron improved the cognitive functions [220].

In another study, *C. sativus* extract administration for 3 months significantly increased white blood cell count in patients who had normal white blood cell count compared to crocin or placebo. No significant change was observed in hematologic factors during the study [221].

## 7. Safety and Toxicity Studies of *Crocus* Plants

The analysis of medicinal plants for toxicity is fundamental for their reliable and safe use among consumers [224]. Several investigations should be carried out to assure the safety of bioactive compounds. Indeed, preclinical studies based on animal toxicity are fundamental steps to determine the toxicity of drugs. In this step, studies focused on the determination of lethal dose (LD_50_) and the toxicity against several organs (vital organs). Moreover, teratogenicity should also be evaluated in preclinical studies.

Preclinical toxicological studies of *C. sativus* and its bioactive cells were investigated by several studies in various animal models and different modes of administration.

Stigma and petal extracts of saffron exhibited moderated toxicological effects in mice using intraperitoneal administration. The LD_50_ values are 1.6 and 6 g/kg for stigma and petal extracts, respectively [225]. However, the oral administration in mice of total saffron showed an LD_50_ of 4120 mg/kg [225] (Table 4).

The ethanolic extract of saffron (stigma) showed significant effects using subacute doses in rats of the ethanolic extract (0.35, 0.7, and 1.05 g/kg i.p., for 2 weeks) that caused significant reductions in the hemoglobin (Hb) and hematocrit (HCT) levels and total red blood cell (RBC) count [226]. Moreover, the total white blood cell (WBC) count showed significant dose-dependent increases in extract-treated rats. The ethanolic extract has also exhibited necessary increases of AST, ALT, urea, uric acid, and creatinine levels, which were dose-dependent. It was also shown that an ethanolic extract increased the levels of some enzymes involved in liver injury, in particular, ALT and AST [226]. Moreover, the histopathological findings reported that ethanolic extract induced mild to severe hepatic and renal injuries, thus supporting the biochemical analysis [226].

In another study also carried out by Mohajeri et al. [227], a total extract of saffron administered in rats (0.35, 0.70, and 1.05 g/kg i.p., for 2 weeks) showed some toxicity in the given doses and caused major hepatic and renal tissue damages. The aqueous extract of saffron administered intraperitoneally at 25-100 mg/kg increased survival in rats so that no mortality was observed at a dose of 10 mg/kg [228]. In another subacute study carried out by Karimi et al. [229], the aqueous extract of the petal (1.2, 2.4, and 3.6 g/kg) and stigma (0.16, 0.32, and 0.48 g/kg) of saffron administered intraperitoneally showed a significant decrease of body weight in rats. The biochemical analysis revealed that both extracts reduced the levels of Hb, HCT, and RBC counts. Moreover, both extracts produced anemia [229]. In another study, Khayatnouri et al. [230] have evaluated the effect of saffron on the spermatogenesis index in rats. The authors showed that saffron administered at 200 mg/kg of saffron for 28 days exhibited significantly decreased spermatogenesis index, including such indicators as repopulation index, tubular differentiation index, and spermatogenesis index [230].

The subchronic toxicity of saffron was also evaluated in several additional studies [231–235]. Modaghegh et al. [232] have tested the subchronic toxicity of saffron tablets on rats at 200 and 400 mg per day for 1 week. The results showed that saffron might change some hematological and biochemical parameters. However, these adverse effects were within normal ranges because they had not altered clinical parameters [232]. Bahmani et al. [236] have tested the toxicity of the aqueous extract of saffron administered orally at 500, 1000, or 2000 mg/kg/day for three weeks to adult mice and neonates during lactation. The results did not show important toxicity (LD_50_ = 4120 mg/kg) in mice. In addition, the histological analysis indicated that the aqueous extract of saffron did not have any toxic effects. The administration of saffron to BALB-c mice at 4000 and 5000 mg/kg following five weeks exposure significantly decreased RBC and WBC counts and Hb level [231]. Moreover, saffron caused kidney dysfunction revealed by the increase of blood urea nitrogen (BUN) and creatinine levels treated in animals.

Another study carried out by Amin et al. [237] showed that the aqueous saffron extract at lower doses (25, 50, and 100 mg/kg/day, administered intraperitoneally for 30 days) showed no toxicity on treated animals. Moreover, at these doses, this extract protects against ethylene glycol-induced calcium oxalate (CaOx) nephrolithiasis in rats [237]. These findings indicate that saffron extracts possess toxicity at higher doses, while they could present protective effects at lower doses.

In a recent study carried out by Hosseinzadeh et al. [233], an aqueous extract of saffron stigmas, administered intraperitoneally at 20 and 80 mg/kg, showed an important decrease of methyl methanesulfonate-induced DNA damage in mouse organs [233]. A study carried out by Hariri et al. [238] showed that aqueous extract administered intraperitoneally at 50, 100, and 200 mg/kg prevented toxicity induced by diazinon in rats.

The teratogenic effect of aqueous extracts of saffron was investigated in mice by Zeynali et al. [239]. The administration of this extract at 0.8, 0.4, and 0.2% significantly reduced the tail length, biparietal diameter, placental diameter, and weight of the fetus during the gestational period. Moreover, the mortality rate and the mean number of the resorbed fetus were significantly increased in a dose-dependent manner [239]. Edamula et al. [240] have evaluated the prenatal developmental toxicity of saffron in male Wistar rats. The administration of the saffron extract at 1000, 250, and 50 mg/kg had no effects on gravid uterine weight, early and late resorptions, corpora lutea and implantation counts, and food intake [240]. Moreover, skeletal examinations have confirmed the absence of any malformation and biochemical examinations did not show effects in biochemical parameters [240].

Crocin is a major compound of saffron extract that has shown important pharmacological properties. The toxicity evaluation of this component was reported in some studies [241–244]. The acute toxicity of crocin on rats and mice was tested by Hosseinzadeh et al. [242]. The results showed that oral and intraperitoneally administration of crocin at 3 g/kg over 2 days did not cause mortality. Moreover, biochemical, hematological, and pathological investigations revealed that crocin did not cause damage to any major organ in the body [242]. Indeed, at 180 mg/kg/day for 21 days, the intraperitoneal administration of crocin increased platelets and creatinine levels. Moreover, the same dose reduced weight, food intake, and alveolar size. Besides, at 90 mg/kg, crocin decreased the levels of albumin and ALP with a significant increase in LDL level [242].

On the other hand, it was previously reported by Wang et al. [241] that crocin induced important black pigmentation of the liver and acute hepatic damage associated with discoloration. These damages were observed only at a higher dosage (100 mg/kg for 2 weeks). However, crocin at 50 mg/kg/day (for 8 days) did not affect hepatic function [241]. Subacute toxicity of crocin on rats was examined in another study by Taheri et al. [243]. The results showed that the administration of crocin at 50, 100, and 200 mg/kg did not show negative effects on biochemical parameters such as ALT, AST, ALP, urea, uric acid, creatinine, MDA, and GSH. Moreover, no significant toxicity was observed using histopathological investigations [243].

On the other hand, the teratogenic effect of crocin was investigated by Moallem et al. [244] in mice. In this work, the intraperitoneal administration of crocin at 200 mg/kg and 600 mg/kg showed a disruption in skeletal formation. Moreover, at the same doses, crocin affected weight, length, growth, mandible, and calvaria of fetuses indicated by the examination of maternal and fetal factors [244].

The acute toxicity of safranal (main compound of saffron) was evaluated in male mice, female mice, and male Wistar rats. The intraperitoneal administration of safranal showed significant toxicity in male mice (LD_50_ = 1.48 mL/kg), female mice (LD_50_ = 1.88 mL/kg), and male Wistar rats (LD_50_ = 1.50 mL/kg). However, in oral administration of safranal, LD_50_ values were 21.42, 11.42, and 5.53 mL/kg in male mice, female mice, and male rats, respectively [225]. In this study, the authors suggested that the significant difference in LD_50_ values after intraperitoneal and oral administration is due to first-pass metabolism and lower absorption after oral exposure [225].

In another study, the subacute toxicity was evaluated in mice and rats. Safranal was administered orally at 0.1, 0.25, and 0.5 mL/kg/day over 21 days [245]. Safranal induced significant decreases in several hematological parameters such as RBC counts, HCT, Hb, and platelets. Moreover, safranal reduced some biochemical factors, including TC, TG, and ALP. Also, no noticeable heart, liver, or spleen lesions were observed after pathological examinations [246]. On the other hand, Riahi-Zanjani et al. [247] have tested the immunotoxic effect of safranal on cellular and humoral cells of the immune system in mice. The results of this work showed that the intraperitoneal administration of safranal at 0.1, 0.5, and 1 mL/kg within 3 weeks days (5 days/week) did not show any significant toxicity on immune system cells [247]. In other studies carried out by Moallem et al. [244], safranal administered at 0.075 and 0.225 mL/kg dysregulated skeletal formation and affected maternal and fetal factors such as weight, length, and growth [244].

The clinical investigations of saffron and its derivatives have also been reported [232, 248, 249]. The examination of saffron safety in healthy volunteers at 200 and 400 mg within 7 days by Modaghegh et al. [232] showed a decrease in arterial pressures, and standing systolic BPs were decreased in persons who received 400 mg [232]. Moreover, at the same concentrations (200 and 400 mg), saffron was found to be a safe drug on the coagulation system [248]. Mohamadpour et al. [249] also investigated the clinical toxicity of crocin in healthy volunteers. In this study, crocin was examined at 20 mg using a randomized, double-blind, placebo-controlled trial. Administrations of crocin tablets partially decreased thromboplastin time, amylase, and mixed WBC (monocytes, basophils, and eosinophils), which showed that crocin is a relatively safe product [249].

## 8. Discussion

*Crocus* plants have been traditionally used for several purposes (e.g., reduce bruises, promote blood circulation, anxiolytic, antitumor, antihyperglycemic, etc.) [147, 181, 253–256]. *Crocus sativus* is listed in the pharmacopoeias of several realms such as Europe, the United Kingdom, Japan, and China [253, 257–259], as well as in other national or local standards.

*Crocus* plants have undergone comprehensive validation, including phytochemical profiling and determination of targeted biological activities. Saffron is a valuable plant whose main components include safranal, crocetin, crocin, and picrocrocin.

It has been shown in bioavailability tests that when administered orally, crocins are not resorbed as such, but only after an intestinal deglycosylation, after which they reach the bloodstream, being able to cross the blood-brain barrier. This is also the reason why a process for concentrating hydroalcoholic extracts has been developed, when a product with more than 90% crocin 1 is finally obtained, which, being subjected to an enzymatic transformation treatment with *β*-glucosidase, provides the active metabolite, trans-crocetin, with a concentration of 70% [260].

Both *in vivo* and *in vitro* studies have shown that much of the biological effectiveness of saffron can be attributed to its antioxidant potential resulting from the synergistic antioxidant capacity of its bioactive ingredients [261]. The antioxidant activity of *C. sativus* is mainly attributable to its antioxidant active constituents such as safranal, crocin, and crocetin [83].

The radical-scavenging activity of *Crocus* plant extracts is associated with its anti-inflammatory activity. Crocin and safranal showed anti-inflammatory and antinociceptive effects in the carrageenan model of inflammation with comparable effect to diclofenac [214]. The saffron extract was reported to possess a more significant radical-scavenging activity than carrot and tomato extracts [135]. Crocin and safranal mediate their antioxidant activities by modulating redox status in human plasma [87], mice [204], and primary hepatocytes of rats [262]. Saffron and crocin ameliorate the effects of *Vipera russelli* venom-induced oxidative stress and hematological alterations in adult mice [173, 263].

Crocetin, as a strong antioxidant compound, was demonstrated to inhibit lipid peroxidation, increase the activity of glutathione *S*-transferase (GST), GPx, CAT, and SOD, decrease damage marker enzymes such as aryl hydrocarbon hydroxylase (AHH), LDH, *γ*-glutamyl transferase (GGT), and adenosine deaminase (ADA) in rat liver tissues, inhibit proliferation of lung cancer cells [174, 264], reduce ROS-induced lipid peroxidation in primary hepatocytes of rats [262], and reduce the levels of oxidized LDL [111]. Crocetin decreased the expression of TNF-*α*, interleukin-1*β*, and induced iNOS in the liver of the hemorrhagic shock model [265]. Crocetin also decreased the indomethacin-induced rise in glutathione in nondiabetic and diabetic rats [266] and reduced ROS generated by B*α*P in mice [204] and angiotensin II-induced ROS [126].

Crocetin was found to be more effective than dimethyl crocetin and safranal as an anticancer and chemopreventive agent [264]. This may be due to the free hydroxyl moiety of the carboxylic group in crocetin that makes it potent for proton donor, thus more reactive to free radicals [203]. Saffron extracts administered orally or topically reduced the *in vivo* incidence of induced cancers, inhibiting tumor growth rate and prolonging the life of test animals. Furthermore, the toxicity of cytostatic drugs (i.e., cisplatin) has been reduced in experimental models in animals.

The cardioprotective effect of crocetin against norepinephrine- (NE-) induced cardiac hypertrophy has been related with its modulation effects of endogenous antioxidant enzymatic activities [267]. However, the synergism between all these bioactive components significantly potentiates the antioxidant capacity of saffron [264].

Aqueous and ethanolic saffron extracts were tested, along with saffron and crocin, for antidepressant effects in mice using the forced swimming test. All proved to have antidepressant action, and the saffron and crocin content of the extracts administered is reflected in the recorded result. Crocin probably works by inhibiting dopamine and NE reuptake, while safranal inhibits serotonin reuptake at the synapse.

Saffron plays an important role in the food industry and home cooking, both as a preservative and a dye for foods and beverages [268].

The antibacterial activity of aqueous extracts of saffron stigmas was observed against *S. aureus*, *Enterococcus faecalis*, and *E. coli* by Cenci-Goga et al. [72]. It was concluded that stigmas of *C. sativus* have enough antimicrobial agents to exhibit preservative function in foods depending on its compatibility with the product [72]. Saffron, which contains carotenoids (mainly crocin), shows antiseptic activity as its alcoholic compounds can easily alter the cell protein nature and impair the permeability of the cell membranes [71]. In one more study, it was reported that the presence of antimicrobial agents in saffron stigma suppressed microbial growth. Cosano et al. [269], who considered several saffron products from different producing countries (Spain, Iran, Italy, Greece, and Morocco), showed saffron to be a safe additive having neither microbial load nor health risk to foods after addition when there are no other food-preservation methods [269]. A research carried out by Pintado et al. [270] introduced safranal and crocin as biologically active compounds responsible for bacterial growth inhibition. They reported that safranal and crocin could significantly preserve foods against *Salmonella*, *E. coli*, and *S. aureus* when added to foodstuff [270]. Abbasvali et al. [271] reported that an aqueous extract of saffron petals at a concentration of 5 mg/mL had strong antibacterial effects against *S. aureus.* They studied the preservation effects of petal extracts on the shelf life of shrimp and observed that aqueous petal extracts prolonged the shelf life of shrimp from 3 to 9 days. The influence of phenolic components on bacterial cell walls as well as chelation of metal ions necessary for microbial growth was deemed to be the main food-preservation factor. They concluded that aqueous saffron petal extracts contain significantly more phenolic compounds compared to either ethanolic or methanolic extracts and consequently showed stronger antimicrobial effects.

Another example of using saffron extract as a food preservative was reported by Aktypis et al. [272] in which saffron was added to ovine cheese. They found that saffron, as a natural additive, owed a mild reduction in bacterial growth over a month (30 days) after production weight *n* is being kept at 4°C. Saffron not only made the food functional but also imparted a pleasant flavor to the product [272]. Phenolics, especially flavonoids, usually have strong antioxidant capacities, and thus, herbs rich in them are frequently used as antioxidant food supplements [138, 261]. Cosano et al. [269], who considered several saffron products from different producing countries (Spain, Iran, Italy, Greece, and Morocco), showed saffron to be a safe additive having neither microbial load nor health risk to foods after addition when there are no other food-preservation methods [269].

The limitations are represented by the toxicological evaluation of *Crocus* species which has not been widely studied, and the majority of toxicity tests have focused mainly on *C. sativus* and its major compounds. Toxicological investigations of saffron were carried out only in several studies [225, 227, 228, 232, 236, 238, 251]. The toxicological findings for saffron are not uniform and have some variability depending on plant parts used and experimental models.

To summarize, saffron is a valuable additive containing biologically active compounds which can add functional properties to food products. Saffron powder as well as extract can dramatically influence microbial growth when added to foods and served to preserve foods from spoilage. It should be noted that using saffron does have some limitations, such as an unacceptable flavor of food when used in large enough quantities to ensure high levels of preservation. Combination of saffron with other preservatives (e.g., salt and acid) or merging with other food-preservation methods (freezing, thermal processing) has been proposed to solve this limitation.

## 9. Overall Conclusions and Future Perspectives

Saffron has been used in several traditional medicinal systems against several diseases, including asthma, cardiovascular disease, depression, digestive ailments, and insomnia.

The *Crocus* plants, *C. sativus* being the most studied, comprise a matrix of phytochemicals promising for biotechnological and pharmaceutical purposes. The pharmaceutical and biotechnological industries are continuously searching for potential functional components in the plant kingdom, and there is excellent economic interest if it is determined to be more economical to obtain these compounds by extracting them from the plants as opposed to their chemical synthesis. In the case of saffron, the extraction of its phytocomponents may be limited by economic aspects. However, the study of the phytochemical profile of saffron and the study of the biological activities of these components may lead to the discovery of new mimetics of these compounds or improvements in the laboratory syntheses. Advances in plant research have been realized to develop saffron with better profiles for the food industry but not for pharmaceutical and biotechnological industries. Crocins, picrocrocin, and safranal are relevant in terms of aroma and taste, but as it has been exposed in this review, these components also possess interesting biological properties. A small number of clinical trials have been performed using saffron as a potential agent for anti-Alzheimer's, antidepressant, and antischizophrenia effects. Further efforts are needed to study the other biological effects shown in *in vitro* and *in vivo* studies in well-designed trials in humans.

## Figures and Tables

**Table 1:** *In vitro* biological activities of *Crocus* plants.

Extract/compound	Tested cell lines/methods of analysis	Effect/mechanisms	Ref.
*Cytotoxic activity*			
Saffron/aqueous extract	A549 lung cancer cells MTT Morphological change: inverted microscope Apoptosis: flow cytometry	IC_50_ = 390 *μ*g/mL Inhibition and shrinkage of cancer cells ↑percentage of early and late apoptotic cells	[60]
Crocin/aqueous extract	MCF-7 breast cancer cells MTT Apoptosis: flow cytometry Caspase-7, caspase-9, P53, and PARP: western blot	IC_50_ = 3.5 mg/mL Crocin and paclitaxel: ↑apoptosis, ↑caspase-7, ↑caspase-9, ↑p53, and ↑PARP	[61]
Saffron and its derivatives/ethanolic extract	HeLa human cervical epithelioid carcinoma cells Cytotoxicity assay Morphological change: microscopy	IC_50_ = 2.3 mg/mL for saffron, IC_50_ = 3 mM for crocin, IC_50_ = 0.8 mM for safranal, and IC_50_ = 3 mM for picrocrocin ↑cytotoxicity	[63]
Saffron	HeLa human cervical epithelioid carcinoma cells colony formation inhibitory assay	↓tumor cell growth Trans-crocin 3: inhibitory effect	[90]
Crocin	HL-60 leukaemia cells Apoptosis: flow cytometry MTT	IC_50_ = 0.625 − 10 mg/mL ↓cell proliferation dose-dependently ↑cell cycle arrest at the G0-G1 phase	[64]
Saffron juice	Caco-2 colon cancer cells MTT	IC_50_ = 50 *μ*L/m ↓cell viability	[65]
Saffron/aqueous extract	MCF-7 breast cancer cells gene expression level of MMP using RT PCR trypan blue test	↓MMP gene expression	[62]
*Antimicrobial activity*			
*Crocus sativus/*petroleum ether, methanolic extracts	Agar well diffusion	Petroleum ether extract: effective against *Proteus vulgaris*, *Bacillus subtilis*, *Pseudomonas aeruginosa* methanolic extract: ↓development of *S. aureus*, *E. coli*	[69]
*Crocus sativus/*two extracts one contained the aglycon part of flavonoids the other contained flavonoids glycosides	Agar well diffusion	The extract that contained the glycosidic part of flavonoids exhibited weak antimicrobial activity	[70]
Saffron/aqueous extract	Modified well plate test	↓growth inhibition zone tested pathogens: *E. coli*, *S. aureus*, and *S. faecalis*	[72]
*Antioxidant activity*			
*Crocus chrysanthus* (Herb.)/ethyl acetate, methanol, and water extracts	DPPH reductive potentials, metal chelating phosphomolybdenum method	The water extract showed the most powerful antioxidant activity	[81]
Crocin, saffron/ethanolic extract	Antihemolysis activity DPPH, lipid peroxidation Phosphomolybdenum method	The saffron extract exhibited 107 mg *α*-tocopherol/g DPPH radical-scavenging activity and 98.3, 90.8, and 33.1 mg *α*-tocopherol/g, respectively, for crocin-1, crocin-2, and crocin-3	[82]
Saffron/ethanolic, methanolic extract	DPPH ferric reducing antioxidant power	Methanolic extract 300 *μ*g/mL: ↑↑antioxidant activity	[83]
Saffron/corms, tepals, and leaves	*β*-Carotene/linoleate model system, reducing power, DPPH, NO, radical scavenging, iron, and copper chelation	The best antioxidant activity: leaves and tepal extract, the least antioxidant activity: corms	[84]
Saffron/aqueous extract	Bronchial epithelial cells	↓NO, ↓iNOS, and ↓peroxynitrite ion generation ↓cytochrome c release	[86]
*Antidiabetic*			
*Crocus* chrysanthus (Herb.)/ethyl acetate, methanol, and aqueous extracts	*α*-Glucosidase inhibition *α*-Amylase inhibition	*α*-Glucosidase inhibition: 14.8-1.89 mmol acarbose equivalent/g according to different extracts *α*-Amylase inhibition: 0.8-0.15 mmol acarbose equivalent/g	[81]

Abbreviations and symbols: ↑ increased, ↓ decreased, 2,2-diphenyl-1-picryl-hydrazyl-hydrate (DPPH), induced nitric oxide synthase (iNOS), nitric oxide (NO), and poly (ADP-ribose) polymerase (PARP).

**Table 2:** *In vivo* evaluation of the pharmacological properties of *Crocus* plant.

Plant/compound/extract	Doses	Route	Model	Main pharmacological effect	Ref.
Crocetin	100 mg/kg	Oral	Rats stroke-prone spontaneously hypertensive rats high-oxidative stress model	Antioxidant ↓oxidative stress, ↓ROS in rats brain	[95]
*Crocus sativus* L./aqueous extract	10, 20, and 40 mg/kg	i.p.	Rats STZ-induced diabetes	Antihyperglycemic ↓blood glucose, ↓MDA, ↓NO, ↓lipids, ↓TG, ↓cholesterol, ↑glutathione level, ↑CAT, ↑SOD ↓inflammatory cytokines	[180]
*Crocus sativus* L./aqueous, ethanol extracts	500 mg/kg	i.v.	Rats, guinea pigs	Antihypertensive ↓blood pressure in a dose-dependent manner	[100]
Crocetin	20 mg/kg	Oral	Lung cancer-bearing mice benzo(a)pyrene- (B(a)p-) induced lung carcinoma	Antitumor ↑activities of enzymatic antioxidants ↑glutathione metabolizing enzymes	[203]
Crocetin	50 mg/kg	i.p.	Mice Benzopyrene-induced lung cancer model	Antitumor ↓proliferating cells	[204]
Zhejiang saffron	100 mg/kg	Oral	Mice Xenograft tumor	Antitumor ↓tumor size *via* caspase-3, caspase-8, caspase-9, and ↑apoptosis	[205]
Saffron/aqueous infusion	50-500 mg/kg	Oral	Mice DMBA-induced skin carcinogenesis	Antitumor ↓papilloma cells formation	[206]
Saffron/aqueous extract	100 mg/kg	Topical	Mice DMBA, croton oil-induced skin carcinogenesis, MCA-induced soft tissue sarcomas	Antitumor ↓tumor formation	[207]
Saffron	400, 800 mg/kg	Oral	Rats PTZ-induced seizures	Anticonvulsant ↓seizures frequency in a dose-dependent manner	[128]
Safranal	0.15, 0.35 mL/kg	i.p.	Mice PTZ-induced seizures	Anticonvulsant ↓seizures duration, delayed the onset of tonic convulsions	[16]
Safranal	72.75, 145.5, and 290 mg/kg	i.p.	Rats PTZ-induced seizures	Anticonvulsant ↓MCS, ↓GTCS	[17]
Crocin	200 mg/kg	i.p.	Mice PTZ-induced seizures	It did not show anticonvulsant activity	[16]
*C. sativus* L./hydroethanolic extract	50 mg/kg	Oral	Meriones shawi Pb-intoxicated (25 mg/kg bw, i.p.)	Neuroprotective ↑TH in SNC, VTA, LC, DS, and MFB ↑locomotor activity, ↓dysfunction in Pb-intoxicated meriones	[141]
Crocetin	25, 50, 75 *μ*g/kg	i.p.	Rats 6-Hydroxydopamine (10 *μ*g intrastriatal) Induced Parkinson's disease	Neuroprotective ↓dopamine utilization by tissues	[145]
Saffron	0.01% *w*/*v*	Oral	BALB-c mice MPTP-induced Parkinson's disease	Neuroprotective ↓ROS, ↑antioxidant effect → protect dopaminergic cells	[146]
*C. sativus* L./stigma extract	100 mg/kg	Oral	Rats Induction of cerebral ischemia MCAO	Neuroprotective ↓SOD, ↓CAT, ↓Na^+^, and K^+^-ATPase activities ↓glutamate, ↓aspartate induced by ischemia	[164]
Saffron/honey syrup	200, 500 mg/kg	Oral	Mice Aluminum chloride-induced neurotoxicity	Neuroprotective ↓neurotoxicity	[208]
Saffron/aqueous extract	50, 100, 200 mg/kg	Oral	Rats Diazinon- (20 mg/kg) induced neurotoxicity	Neuroprotective ↓inflammation, ↓oxidative stress, and ↓neuronal damage	[88]
Crocin	30, 60, 120 mg/kg	Oral	Rats Ischemia/reperfusion injury model of stroke	Neuroprotective Crocin 60 mg/kg → ↓brain oedema	[158]
Crocetin	50 mg/kg	Oral	Rats Induced cerebral contusion	Neuroprotective ↑neurological function, ↓neuronal apoptosis ↑VEGFR-2, ↑SRF	[155]
Crocin	25, 50 mg/kg	i.p.	Rats Retinal damage induced by ↑intraocular pressure	Retinal damage protection ↑RGCs, ↓apoptosis, and ↑PI3K/AKT	[197]
Crocetin	100 mg/kg	Oral	Mice N-Methyl-d-aspartate in the murine retina	Retinal damage protection ↓NMDA, ↓GCL cell number, ↓TUNEL-positive cells, ↓b-wave amplitude, ↑caspase-3/7, and ↑caspase-3 in the GCL	[195]
Saffron/aqueous extract	50, 100, 150, and 250 mg/kg	i.p.	Rats	Effect on brain neurotransmitters ↑dopamine, ↑glutamate in a dose-dependent manner No effect on brain serotonin, norepinephrine	[209]
Saffron/aqueous, ethanolic extracts	80–320, 400–800 mg/kg	i.p.	Mice Naloxone-induced model	Effect on opioid system ↓morphine withdrawal signs ↓locomotor activity (open-field test)	[171]
Saffron/ethanolic extract safranal	10, 50, and 100 mg/kg 1, 5, and 10 mg/kg	i.p.	Mice	Effect on opioid system ↓acquisition, ↓expression of morphine conditioning place preference	[210]
Crocin	400, 600 mg/kg	i.p.	Mice	Effect on opioid system ↓acquisition, ↓reinstatement of morphine-induced conditioning place preference	[211]
*Crocus sativus* L./ethanolic extract from stigma	5, 10 *μ*g/kg	Intra-accumbal	Rats	Effect on opioid system ↓time spent on the drug paired side, ↓expression of morphine conditioning place preference	[212]
*Crocus sativus* L./aqueous, ethanolic extracts from stigma safranal crocin	Extracts 0.2-0.8 g/kg Safranal 0.15-0.5 mL/kg Crocin 50-600 mg/kg	i.p.	Mice Forced open-field swimming test	Antidepressant Extracts, safranal, crocin: ↓immobility time, ↑stereotypic activities Safranal: ↑swimming time Safranal and crocin: ↑climbing time Crocin: ↑dopamine, ↑norepinephrine Safranal: ↑serotonin	[18]
*Crocus sativus* L./aqueous extract	30 mg/kg	ICV	Rats STZ-ICV Alzheimer's disease model	Anti-Alzheimer ↓cognitive deficits ↑learning, ↑memory *via* metabolism/enzyme mechanisms, no anatomical structural repair involved	[136]
Saffron	60 mg/kg	i.p.	BALB-c mice adult and aged	Cognitive enhancing effect ↑learning, ↑memory (passive avoidance behavior test) ↓AChE in adult mice No effect on AChE activity on aged mice	[213]
Crocin	30 mg/kg	ICV	Rats STZ-ICV Alzheimer's disease model	Anti-Alzheimer ↑memory (passive avoidance test) ↑spatial cognition (Y-maze task)	[137]
Crocin	25-100 mg/kg	Oral	Rats Diet-induced hyperlipidemia	**Hypolipidemic** ↑faecal excretion of fat and cholesterol Not influence the elimination of bile acids ↓pancreatic lipase as a competitive inhibitor	[175]
Crocin safranal	25, 50, and 100 mg/kg 0.5, 1, and 2 mg/kg	i.p.l.	Rats Local inflammation induced by i.p.l. injection of carrageenan (100 *μ*L, 2%)	Anti-inflammatory ↓oedema, ↓inflammatory pain responses ↓neutrophils	[214]
*Crocus sativus* L./ethanolic extract from stigma	20, 40, and 80 mg/kg	Oral	Rats Alloxan-induced diabetes	Antihyperglycemic 40 mg/kg: ↓blood glucose, ↑serum insulin	[182]
Safranal	0.2, 0.5, 0.75 mL/kg	Aerosol	Guinea pigs' citric acid aerosol for 10 min	Antitussive ↓cough count significantly as compared to the saline-treated group	[189]

Abbreviations and symbols: ↑ increase, ↓ decrease, body weight (bw), catalase (CAT), dimethylbenzene [a] anthracene (DMBA), dorsal striatum (DS), generalized tonic-clonic seizures (GTCS), intracerebroventricular (ICV), intraperitoneal (i.p.), intraplantar (i.p.l.), intravenously (i.v.), locus coeruleus (LC), 20-methylcholanthrene (MCA), 1-methyl-4-phenyl-1,2,3,6-tetrahydropyridine (MPTP), malondialdehyde (MDA), medial forebrain bundle (MFB), middle cerebral artery occlusion (MCAO), minimal clonic seizures (MCS), nitric oxide (NO), pentylenetetrazole (PTZ), phosphatidylinositol 3-kinase (*PI3K*)/protein kinase B (*AKT*), reactive oxygen species (ROS), retinal ganglion cells (RGCs), serum response factor (SRF), substantia nigra compacta (SNc), superoxide dismutase (SOD), streptozotocin (STZ), triglycerides (TG), tyrosine hydroxylase (TH), ventral tegmental area (VTA), and vascular endothelial growth factor receptor-2 (VEGFR-2).

**Table 3:** Clinical studies.

Type of extract	Type of study	Dose/period	Effect	References
Saffron extract	Randomized double-blind clinical trial	30 mg/day Six weeks	Saffron supplements statistically improved the mood of subjects compared to the placebo group based on the Hamilton depression rating scale (HAM-D)	[222]
Saffron extract	Randomized double-blind clinical trial	30 mg/day for six weeks	Treatment of mild to moderate depression ↓clinical signs	[215]
Hydroalcoholic extract of *Crocus sativus* L. coadministered with fluoxetine	Randomized double-blind clinical trial	(40 or 80 mg) (30 mg/day) Six weeks	*C. sativus* 80 mg plus fluoxetine was more effective in the treatment of mild to moderate depressive disorders	[223]
Crocin/aqueous extract	Clinical trial	15 mg twice daily	Well tolerated by schizophrenic patients with no severe side effects	[221]
Saffron extract (Affron®)	Randomized, double-blind, placebo-controlled study, youth aged 12–16 years	14 mg twice daily 8 weeks	Improved anxiety and depressive symptoms	[218]

**Table 4:** Toxicological studies of *Crocus sativus* L.

Extract/compound	Doses	*In vitro*/*in vivo*	Route of administration	Model	Adverse effects	Ref.
Aqueous extract	1.2-2 g/bw	*In vivo*	Intraperitoneal	Mice	Nausea, vomiting, diarrhea, bleeding	[234]
Aqueous extract	4 g/bw daily	*In vivo*	Oral	Mice	Nontoxic	[235]
Aqueous extract	IC_50_ = 50 − 400 mg/mL	*In vitro* cytotoxic assay	—	CCD-18Lu Human normal lung cells	Noncytotoxic	[90]
Aqueous extract	500, 1000, and 2000 mg/kg daily saffron, three weeks	*In vivo*	Oral	Mice Neonates mice during lactation	LD_50_ = 4120 mg/kg ↑morphological changes in the kidney of neonates	[236]
Aqueous extract	50, 100, and 200 mg/kg	*In vivo*	Intraperitoneal	Rats Diazinon-induced toxicity	Prevented the toxicity induced by diazinon in rats	[238]
Ethanol extract stigmas	0.35, 0.70, 1.05 g/kg daily	*In vivo*	Intraperitoneal	Rats Subacute toxicity	↓Hb, ↓HCT, ↓RBC ↑AST, ↑ALT, ↑urea, ↑uric acid ↑creatinine ↑hepatic and renal tissue injuries, dose-dependent	[227]
Ethanol extract	2 mg/kg of cisplatin	*In vivo*	Oral	Mice Cisplatin-induced toxicity	↑life span of cisplatin-treated mice almost threefold	[250]
Aqueous extract	25-100 mg/kg	*In vivo*	Intraperitoneal	Rats Acute and subacute toxicity	↑survival No mortality at dose 10 mg/kg	[228]
Aqueous extract	Several doses	*In vivo*	Oral	Rats	21.42 mL/kg	[251]
Aqueous extract	Several doses	*In vivo*	Intraperitoneal	Rats	1.48 mL/kg	
Aqueous extract	Several doses	*In vivo*	Oral	Mice	5.53 mL/kg	
Aqueous extract	Several doses	*In vivo*	Intraperitoneal	Mice	3500 mg/kg	
Total extract	0.35, 0.70, and 1.05 g/kg	*In vivo*	Intraperitoneal	Rats	Hepatic, renal tissue damages anemia ↓Hb, ↓HCT, ↓RBC	[226]
Crocetin	10, 25, 50, 100, and 200 mM	*In vitro*	—	Frog (*Xenopus*) embryos	Crocetin is a teratogen, but less potent than ATRA	[252]
Safranal	0.1, 0.5, 1 mL/kg	*In vivo*	Intraperitoneal	Rats Immunotoxin effect	Showed important toxicity than other active constituents in saffron stigma	[247]
Safranal	1.2 mL/kg	*In vivo*	Intraperitoneal	Rats Acute, subacute toxicity	↓cytotoxicity	[228]
Safranal	1.2 mL/kg	*In vivo*	Intraperitoneal	Mice	LD_50_ = 1.48 mL/kg	[225]
Safranal	1.2 mL/kg	*In vivo*	Intraperitoneal	Mice	LD_50_ = 1.88 mL/kg	
Safranal	1.2 mL/kg	*In vivo*	Intraperitoneal	Rats	LD_50_ = 1.50 mL/kg	
Safranal	1.2 mL/kg	*In vivo*	Oral	Mice	LD_50_ = 21.42 mL/kg in	
Safranal	1.2 mL/kg	*In vivo*	Oral	Mice	LD_50_ = 11.42 mL/kg	
Safranal	1.2 mL/kg	*In vivo*	Oral	Rats	LD_50_ = 5.53 mL/kg	
Crocin	150-210 g	*In vivo*	Oral, intraperitoneal	Mice Rats	↑platelets, ↑creatinine ↓food intake	[242]

Abbreviations and symbols: ↑ increase, ↓ decrease, ALT (alanine aminotransferase), AST (aspartate transaminase), ATRA (all-trans retinoic acid), Hb (hemoglobin), HCT (hematocrit), and RBC (red blood cell).

## Data Availability

All the data used to support the findings of this study are included within the article.

## Abbreviations

AChE: Acetylcholinesterase
ADA: Adenosine deaminase
AHH: Aryl hydrocarbon hydroxylase
ALP: Alkaline phosphatase
ALT: Alanine aminotransferase
AST: Aspartate transaminase
AV: Atrioventricular
A*β*: Amyloid *β*
BHT: Butyl hydroxyl toluene
BP: Blood pressure
BUN: Blood urea nitrogen
CaOx: Calcium oxalate
CAT: Catalase
CK: Creatine kinase
CMEC: Cortical microvascular endothelial cells
CNS: Central nervous system
DAD: Diode-array detection
DEN: Dimethylnitrosamine
DOCA: Desoxycorticosterone acetate
DPPH, 2: 2-Diphenyl-1-picryl-hydrazyl-hydrate
ESI: Electrospray ionization
FRAP: Fluorescence recovery after photobleaching
GC: Gas chromatography
GGT: *γ*-Glutamyl transferase
GPx: Glutathione peroxidase
GSH: Reduced glutathione
GSSG: Oxidized glutathione
GST: Glutathione S-transferase
Hb: Hemoglobin
HCT: Hematocrit
HPLC: Performance liquid chromatography
HPTLC: High-performance thin-layer chromatography
ICV: Intracerebroventricular
iNOS: Induced nitric oxide synthase
ISO: Isoproterenol
LC: Liquid chromatography
LDH: Lactate dehydrogenase
LDL: Low-density lipoprotein
MDA: Malondialdehyde
MDD: Major depressive disorder
MES: Maximal electroshock seizure
MMP: Matrix metalloproteinase
MPTP: 1-Methyl-4-phenyl-1,2,3,6-tetrahydropyridine
MS: Mass spectrometry
NE: Norepinephrine
NF-*κ*B: Nuclear factor *κ*B
NMDA: N-Methyl-D-aspartate
NMR: Nuclear magnetic resonance
NO: Nitric oxide production
NS: Nervous system
PARP: Poly (ADP-ribose) polymerase
PI3K/AKT: Phosphatidylinositol 3-kinase
PTZ: Pentylenetetrazole
RBC: Red blood cell
RGCs: Retinal ganglion cells
ROS: Reactive oxygen species
SOD: Superoxide dismutase
SRF: Serum response factor
TBARS: Thiobarbituric acid
TC: Total cholesterol
TG: Triglyceride
TLC: Thin-layer chromatography
TNF-*α*: Tumor necrosis factor-*α*
VCAM-1: Vascular cell adhesion molecule-1
VEGFR-2: Vascular endothelial growth factor receptor-2
VLDL: Very-low-density lipoprotein
WBC: White blood cells.

## Supplementary Materials

Supplementary Materials Table S1: phytoconstituents of *Crocus* species detected by various techniques. Table S2: phytoconstituents of *Crocus* species detected by gas chromatography coupled to mass spectrometry (GC-MS). The Supplementary Material for this article can be found online.
