# Supplementary material for: The Pharmacological Activities of Crocus sativus L.: A Review Based on the Mechanisms and Therapeutic Opportunities of its Phytoconstituents
Source: Oxid Med Cell Longev. 2022 Feb 14;2022:8214821. doi: 10.1155/2022/8214821 (PMC8860555; doi:10.1155/2022/8214821)
Supplement: Supplementary Materials — Table S1: phytoconstituents of Crocus species detected by various techniques. Table S2: phytoconstituents of Crocus species detected by gas chromatography coupled to mass spectrometry (GC-MS). The Supplementary Material for this article can be found online. [file 8214821.f1.docx]

SUPPLEMENTARY MATERIAL

1. Habitat and Cultivation of *Crocus* Plants

1. Habitat

The genus *Crocus* comprises more than eighty-eight corm bearing perennial species. Most *Crocus* species are adapted to the cool Mediterranean environment [1]. They are distributed to Central and Southern Europe, North Africa, Southwest Asia, and Western China [2-5]. *Crocus* species occur in climates chilly or cold winter, rainy spring and autumn, and hot and dry summer.

*C. sativus* is a monocotyledonous triploid plant that is vegetatively propagated because it is genetically sterile. Recently published research on its possible progenitors suggested that the most probable are the species *Crocus cartwrightianus* Herb. and *Crocus pallasii* Goldb. [6]. *C. sativus* is a perennial plant native to southwest Asia [7,8].

It is a low‐rise plant, typically 10 to 25 cm high, and develops from a bulbous-tuberous structure that replicates every year. The flower consists of six tepals, three stamens, and a style which culminates with three red branched stigmas that are used to obtain the spice [9]. It is most probable that this species was first cultivated in Greece and nearby regions [10], while nowadays, it is cultivated in different regions worldwide.

2. Cultivation

It has been reported that the annual production of saffron is 50 tons worldwide, estimating the cost of about 50 million dollars [11]. Primarily the *Crocus* species are cultivated for the production of the spice saffron [12]. The cultivation of saffron has gradually declined, and in some countries, it has almost disappeared [13,14]. *C. sativus* species cannot propagate in the wild and require the involvement of humans to reproduce [15]. The crop generally grows to woodland, scrub, and meadows. It has been reported that *Crocus* species are cultivated in Afghanistan, Australia, Azerbaijan, China, Egypt, France, Greece, India, Iran, Iraq, Israel, Italy, Japan, Pakistan, Morocco, Spain, Switzerland, Turkey, the United Arab Emirates since ancient times [16,17].

2.2.1. Propagation, Planting, and Spacing

*Crocus sativus* is triploid, which is “self-incompatible,” and the male-sterile plant is propagated vegetatively through rhizomes commonly called corms [2]. Corms are dormant in summer, but flower differentiation starts at this season. Studies have shown that dense planting of 67 corms/ m^2^ gave the highest stigma yield per unit area [18]. McGimpsey*, et al.* [19] recommended an intermediate density with an inter-row spacing of 20 cm with an intra-row spacing of 10 cm. It was observed that saffron yield increased in the first one or two years if the corms were planted in higher densities, but become overcrowded and soon required to be dug up and transplanted [19]. The best yield of flowers and corms was obtained with space of 2-3 cm between corms in the row [20].

Greece is the second producer with 5.7 tons, followed by India and Morocco (2.3 tons) [21]. Within the Mediterranean basin, Greece, Spain, and Italy are the main producers. In particular, Italian saffron from L'Aquila, San Gimignano and Sardinia has received the PDO (Protected Designation of Origin) trademark (Reg. CE no. 205 04/02/05 and GUCE L 33 05/03/05 for saffron from L'Aquila and San Gimignano and Reg. CE no. 98 02/02/09 and GUCE L 33 03/02/09 for Sardinian saffron), based on peculiar characteristic and properties, mainly determined by the geographical environment but also including natural and human factors, which strongly support the high quality of these products [22]. It has been reported that about 170 thousand flowers are needed to obtain 1 kg of saffron [23].

2.2.2. Soil

The ***Crocus* species** grow in different soil forms but thrive well in calcareous, humus-rich, and well-drained, deep soil with a pH between 6 and 8 [24]. It has also reported that *Crocus* requires well-ploughed sandy-loamy soil or well-drained clay soil [12]. **Corms** in wet or semi-wet soil types must be well-drained to prevent corms from rotting or getting infected during periods of wet weather.

2.2.3. Climate

*Crocus* species are cultivated in different environmental conditions. Climatic data from various *Crocus* growing areas around the world indicate that the crop can be grown successfully with an average annual rainfall of about 420-1370 mm, and average annual temperatures are around 5.9-18.6 °C. Among several climatic conditions, the temperature is considered as the predominant factor in the control of growth and flowering of bulbs [25]. *Crocus* can thrive well indirect exposure to the sun even though in India, it is cultivated together with almond trees [26]. It has been reported that the best climatic conditions for high yields are rainfall in the autumn, warm summers, and mild winters [12].

2.2.4. Irrigation

Irrigation is not a serious factor in *Crocus* cultivation. The water requirement for saffron cultivation can be fulfilled by the scarce rainfall in the semi-arid conditions. Even in the Mediterranean environment, the crop is not irrigated regularly [20,24]. Skrubis [24] reported that irrigation in early September resulted in quick flowering, while irrigation at the end of September and during October showed higher saffron production. Generally, three kinds of irrigation systems are practised, surface watering, sprinkler irrigation (artificial rain), and drip irrigation. In the case of small scale fields, surface irrigation is used, but it should be avoided in loamy soil with the bad drainage system. Drip irrigation is specially implemented for soils with salinity problems and is suitable for intensive cultivation under plastic or greenhouses.

2.2.5. Fertilizing

Fertilizing is recommended three months before planting. The application of organic manure at 20-30 tons per ha is commonly used all over the world [27,28]. Tammaro [20] reported that mature cow and horse manure of about 25-30 t/ha gave good results. In Greece, NPK fertilizer is applied in a ratio of 4:3:4 [29]. The higher yield of spice was collected from the younger fields in Kashmir that generally had a higher soil nutrient status [30]. The application of urea in winter (from January to March) resulted in a significant increase in flower number in a two-year experiment carried out in Iran [31].

2.2.6. Plant Cycle in Cultivation

There are two stages, active and dormant periods, during a year. The activity period starts when root penetration, sprouting, flowering, and leaf growth take place. During the dormant period, the corms reach the mature stage and are no longer producing new bulbs. This stage is characterized by wilted leaves and dried up roots. There is a transitory period between these two periods in which mitosis and differentiation occur, however, to a less important rhythm [32,33].

2.2.7. Pest and Diseases

One of the main problems in *Crocus* cultivation is the abundance of relatively different pests and diseases. *Rhizoglyphus* spp. (crown mites) which infect *Crocus* leaves that turn yellow with white spots; *Anaphothrips obscurus* (Saffron thrips) infested plants showed silvering and browning of leaves with damaged and deformed fruit. There are several nematodes present in the soil of which the lesion nematode –*Pratylenchus* spp. are mainly damaging the crop. Swellings, chlorosis, wilting lesions, or dark spots are common symptoms of nematode infection. Bacterial rot of *Crocus* is mainly caused by *Bacillus* spp. which spread along with the drift of soil and rain splashes. It has been reported that the corm rot of *Crocus* was initiated by numerous fungal pathogens like *Rhizoctonia crocorum* [34], *Macrophomina phaseolina* [35], *Fusarium oxysporum*, *Fusarium solani*, *Fusarium pallidoroseum*, *Fusarium equiseti*, *Mucor* spp., *Penicillium* spp. [36], *Sclerotium rolfsii* [37]*,* and *Uromyces croci* [38]. Among these notorious fungal pathogens, corm rot of saffron caused by *F. oxysporum* and *F. solani* is considered most destructive [36]. To avoid fungal infection, the best practices are crop rotation, destruction, and burning of infected plants and the use of anti-fungal products before planting, such as benomyl or copper-based solution [26]

| **Table 1.** **Phytoconstituents of *Crocus* species detected by various techniques** | | | | | |
| --- | --- | --- | --- | --- | --- |
| **Classifications** | **Species** | **Parts** | **Chemical component** | **Technique** | **References** |
| Apocarotenoid | *Crocus cartwrightianus* Herb. | Stigma | *trans*-crocetin β-D-gentibiosyl-β-D-neapolitanosyl ester,  *trans-crocetin* di-β-D-gentiobiosyl ester,  *trans-crocetin* β-D-glucosyl-β-D-gentiobiosyl ester,  *cis*-crocetin di-β-D-gentiobiosyl ester | HPLC-ESI-MS | [39] |
| Polyphenolic compositions | *Crocus chrysanthus* (Herb.) Herb. | Flower | galactonic or gluconic acid, proline, nicotinamide, homovanillic acid, tuberonic acid glucoside, *N*-acetylphenylalanine,  quercetin-3-*O*-glucoside-7-*O*-rhamnoside, spiraeoside, eriodictyol-7-*O*-glucoside, luteolin-8-*C*-glucoside, apigenin-6-*C*-glucoside,  tamarixetin-3-*O*-rutinoside, scoparin, pentahydroxyflavone, quercetin-3-*O*-rhamnoside, kaempferol-3-*O*-glucoside,  isorhamnetin-3-*O*-rutinoside, 2*''*-*O*-acetylrutin, kaempferol-3-*O*-galactoside-6*''*-*O*-rhamnoside, tricin, cirsiliol, α-linolenic acid. | HPLC-MS/MS profiles | [40] |
| Monoterpenoids | *Crocus sativus* L. | Pollen | crocusatin-A,  crocusatin-B,  crocusatin-C,  crocusatin-D,  crocusatin-E | ^1^H-NMR spectrum | [41] |
| Lactate |  |  | sodium (2*S*)-(*O*-hydroxyphenyl) lactate | UV, IR, ^1^H-NMR spectrum and mass spectroscopy |  |
| Miscellaneous |  |  | 3,5,5-trimethyl-2-hydroxy-cyclohex-2-en-1,4-dione, 2,4,4-trimethyl-3-formyl-6-hydroxy-2,5-cyclohexadien-1-one,  3,5,5-trimethyl-4-hydroxy-1-cyclohex-2-en-1-one, methylparaben, protocatechuic acid methyl ester,  4-hydroxybenzoic acid, kaempferid, 4-hydroxyphenethyl alcohol, benzoic acid, pyridin-3-yl-methanol, nicotinamide, 1  -*O*-(4-hydroxybenzoyl)-β-D-glucose, adenosine, isorhamnetin-3,4*'*-diglucoside, isorhamnetin-3-*O*-robinobioside, isorhamnetin-3-β-D-glucoside,  5-methyluracil and uracil | UV, IR, ^1^H-NMR spectrum and mass spectroscopy |  |
| Saponins | *Crocus sativus* L. | Corm | 3-*O*-β-D-glucopyranosiduronic acid echinocystic acid 28-*O*-[β-D-galactopyranosyl-(1→2)-α-L-arabinopyranosyl-(1→2)-[β-D-xylopyranosyl-(1→4)]-α-L-arabinopyranosyl-(1→2)-[β-D-xylopyranosyl-(1→4)]-α-L-rhamnopyranosyl-(1→2)-[4-*O*-di-α-L-rhamnopyranosyl-3,16-dihydroxy-10-oxo-hexadecanoyl]-β-D-fucopyranoside  3-*O*-β-D-galactopyranosiduronic acid echinocystic acid 28-*O*-[β-D-galactopyranosyl-(1→2)-α-L-arabinopyranosyl-(1→2)-[β-D-xylopyranosyl-(1→4)]-α-L-arabinopyranosyl-(1→2)-[β-D-xylopyranosyl-(1→4)]-α-L-rhamnopyranosyl-(1→2)-[4-*O*-di-α-L-rhamnopyranosyl-(1→2)-[4-*O*-di-α-L-rhamnopyranosyl-3,16-dihydroxy-10-oxo-hexadecanoyl]-β-D-fucopyranoside | RP-HPLC | [42] |
| Glycosidic carotenoid | *Crocus sativus* L. | Stigma | total crocins (crocins I, II, I*'* and II*'*), picrocrocin, safranal, crocetin | LC-MS, HPLC-DAD-MS & HPTLC | [43] |
| Hydroxycinnamic acid | *Crocus sativus* L. | Corm | sinapic acid and crocetin | LC MS | [44] |
| Flavonoids |  | Leaves | naringenin |  |  |
|  |  | Stigma | dihydrokaempferol 3-*O*-hexoside, kaemferol 7-*O*-bihexoside, kaempferol caffeoyl *p*-coumaroyl diester, isorhamnetin-3-*O*-β-D-glycopyranoside, tamarixetin *O*-kaempferol biflavonoid, taxifolin 7-*O*-hexoside, kaempferol esterified derivative |  |  |
| Carotenoids | *Crocus sativus* L. | Stigma | carotenes, lycopene, zeaxanthin, mangicrocin and xanthone-carotenoid glycosidic conjugate | Not reported | [45-48] |
| Glycosidic Constituents | *Crocus sativus* L. | Stigma | *β*-D-glucosides of (4*R*)-4-hydroxy-3,5,5 trimethylcyclohex-2-enone, (4*S*)-4-hydroxy-3,5,5-trimethylcyclohex-2-enone, (4*S*)-4 (hydroxymethyl)-3,5,5-trimethylcyclohex-2-enone, *β*-D-gentiobiosyl ester of 2-methyl-6-oxohepta-2,4-dienoic acid | UV, MS, NMR | [49] |
| Crocetin |  |  | *β*-D-gentiobiosyl and *β*-D-gentiobiosyl-*β*-D-glucopyranosyl esters |  |  |
| Flavonoid glycosides | *Crocus sativus* L. | Petal Extracts | crocusatin, kaempferol 7-*O*-bihexoside-3-*O* hexoside, dihydrokaempferol 3-*O*-hexoside, taxifolin 7-*O*-hexoside, kaempferol 3-*O*-hexoside, 7-*O*-(acetyl)-hexoside, sinapic acid, kaempferol 3,7-*O*-bihexoside, tribulusterine, 4*'*-methyl ether dihydrokaempferol-3-*O*-deoxyhexoside, kaempferol 3-*O*-(*p*-coumaroyl)-bihexoside, tribulusterine derivative, quercetin 3,7-*O*-bihexoside, quercetin 3-*O*-bihexoside, sinapic acid derivative, kaempferol 3-*O*-(acetyl)-hexoside-7-*O*-hexoside, adenosine, tamarixetin 3-*O*-bihexoside, kaempferol 3-*O*-bihexoside, naringenin 7-*O*-hexoside, isorhamnetin 3-*O*-(desoxyhexosyl)-hexoside, kaempferol 7-*O*-hexoside, quercetin 3,3*'*-dimethyl ether 7-*O*-bihexoside, tamarixetin *O*-kaempferol biflavonoid hexoside, kaempferol 3-*O*-(acetyl)-bihexoside, kaempferol 3-*O*-hexoside, naringenin, kaempferol 3-*O*-(acetyl)-hexoside, quercetin 3-*O*-(*p*-coumaroyl)-hexoside, kaempferol caffeoyl, *p*-coumaroyl diester, 3,3*'*,4*'*-trimethyl ether, quercetin 7-*O*-bihexoside, tamarixetin caffeoyl, *p*-coumaroyl diester, kaempferol, rhamnetin, kaempferol esterified derivative. | LC-DAD-MS (ESI+) Analysis | [50] |
| Saffron metabolite | *Crocus sativus* L. | Stigma | picrocrocin, 2,6,6-trimethyl-4-hydroxy-1-carboxaldehyde-1-cyclohexene (HTCC), 3-gentiobiosyl-kaempherol (kaempferol), α-crocin, crocin 2, crocin 3, safranal, crocin 4, crocin 5, crocin 6 | RP-HPLC | [51] |
| Phenolic fingerprint | *Crocus sativus* L. | Stigma | gallic acid, catechin, chlorogenic acid, vanillic acid, epicatechin, 3-hydroxybenzoic acid, isovanillin, rutin, naringin, benzoic acid, *o*-coumaric acid, quercetin, crocin, safranal | HPLC-PDA | [52] |
|  |  | Byproducts (Tepals + Anthers) | gallic acid, catechin, chlorogenic acid, vanillic acid, epicatechin, isovanillin, rutin, benzoic acid, quercetin, crocin, safranal |  |  |
| Flavonoids and aglycones Q & K | *Crocus vernus* (L.) Hill | Tepals | quercetin−3,4´-*O*-diglucoside, quercetin−3-*O*-glucoside, kaempferol−3-*O*-rutinoside, quercetin, kaempferol | RP-HPLC | [53] |
| Flavonoid glycosides in non hydrolyzed state, and flavonoid aglycones in hydrolyzed state | *Crocus malyi* Vis. |  | kaempferol-3-*O*-rutinoside, quercetin-3-*O*-glucoside, quercetin-3,4*'*-*O*-diglucoside, kaempferol, quercetin |  |  |
|  | *Crocus caeruleus* Weston |  |  |  |  |
|  | *Crocus vernus* (L.) Hill |  |  |  |  |
| Bidesmosidic saponins | *Crocus sativus* L. | Corms | 3-*O*-β-D-glucopyranosiduronic acid echinocystic acid 28-*O*-β-galactopyranosyl-(1→2)-α-L-arabinopyranosyl-(1→2)-[β-D-xylopyranosyl-(1→4)]-α-D-rhamnopyranosyl-(1→2)-[4-*O*-di-α-L-rhamnopyranosyl-3,16-dihydroxy-10-oxo-hexadecanoyl]-α-D-fucopyranoside | RP-HPLC , ^1^H and ^13^C NMR | [54] |
| Carotenoids | *Crocus sativus* L. | Not reported | (4*R*)-4-hydroxy-2,6,6-trimethylcyclohex-1-enecarbaldehyde *O*-β-D-gentiobioside, (4*R*)-4-hydroxy-2,6,6-trimethylcyclohex-1-enecarboxylic acid *O*-β-D-glucopyranoside, 6-hydroxy-3-(hydroxymethyl)-2,4,4-trimethylcyclohexa-2,5-dienone 6-*O*-β-D-glucopyranoside, (2*Z*)-3-methylpent-2-enedioic acid 1-[1-(2,4,4-trimethyl-3,6-dioxocyclohexenyloxy)-*O*-β-D-glucopyranosid-6-yl] ester, (5*S*)-5-hydroxy-7,7-dimethyl-4,5,6,7-tetrahydro-3*H*-isobenzofuran-1-one *O*-β-D-glucopyranoside, (1*R*,5*S*,6*R*)-5-(hydroxymethyl)-4,4,6-trimethyl-7-oxabicyclo[4.1.0]heptan-2-one *O*-β-D-glucopyranoside, and (1*R*)-3,5,5-trimethylcyclohex-3-enol *O*-β-D-glucopyranoside | UV, FTIR, MS, NMR, CD | [55] |
| Carotenoids | *Crocus sativus* L. | Stigmas | picrocrocin, crocin 1, crocin 2, crocin 3 | LC profiles | [56] |
| Crocins, Flavonols and Anthocyanins | *Crocus sativus* L. | Stigmas, Spaths and Leaves | crocin-5-*trans*, crocin-5-*cis*, crocin-4-*trans*, crocin-4-*cis*, crocin-3-*trans*, crocin-3-*cis*, crocin-2-*trans*, crocin-2-*cis*, crocin-20-*trans*, crocin-20-*cis*, crocin-1-*trans*, crocin derivative, kaempferol 3,7-di-*O*-glucoside, picrocrocin, malvidin 3-*O*-glucoside, malvidin 3-*p*-coumaroyl-glucoside, malvidin, malvidin 3-*p*-coumaroyl rutinoside, malvidin derivative, quercetin-3,4*'*-di-*O*-glucoside, luteolin-C-[(*O*-caffeoyl-hexosyl)-*O*-hexoside), unidentified with saccharide moieties, isorhamnetin 3-*O*-rutinoside or isorhamnetin 3-*O*-(desoxyhexosyl)-hexoside, isorhamnetin 3-*O*-rutinoside, unidentified with malonylglucosyl moieties, isorhamnetin 3-rutinoside-4*'*-rhamnoside, isorhamnetin-*O*-glucoside, unidentified with malonylglucosyl moieties | LC-DAD-ESI-MS^n^ data. | [17] |
| Anthocyanins | *Crocus sativus* L. | Tepals | delphinidin 3,5-di-*O*-β-glucoside, delphinidin 3-*O*-glucoside, petunidin 3,5-di-*O*-β-glucoside, petunidin 3-*O*-glucoside. | NMR | [22] |
| Flavanoids |  |  | kaempferol *tri*‐hexoside, kaempferol di‐hexoside, myricetin di‐hexoside, quercetin 3,4′‐di*‐O*‐glucoside, isorhamnetin di‐hexoside, kaempferol‐3‐*O*‐(2‐rhamnosyl)‐glucoside, quercetin‐hexoside‐hexoside acetylated, apigenin dihexoside, kaempferol hexoside‐hexoside acetylated, kaempferol 3‐*O*‐glucoside | HPLC‐DAD |  |
| Anthocyanins | *Crocus etruscus* Parl. | Perianth | delphinidin 3,5-*di*-*O*-β-glucoside, petunidin 3,5-*di*-*O*-β-glucoside | HPLC and UV-spectra | [57] |
| Flavonoids |  |  | quercetin 3,4*'*-*di*-*O*-β-glucoside, kaempferol 3,4'-*di*-*O*-β-glucoside, isorhamnetin 3,4'-*di*-*O*-β-glucoside, myricetin 3-*O*-α-(2-*O*-β-glucosyl)-rhamnoside-7-*O*-β-glucoside, quercetin 3-*O*-α-(2-*O*-β-glucosyl)-rhamnoside-7-*O*-β-glucoside, kaempferol 3-*O*-α-(2-*O*-β-glucosyl)-rhamnoside-7-*O*-β-glucoside, kaempferol 3-*O*-α-(2-*O*-β-glucosyl)-rhamnoside-7-*O*-β-(6-*O*-malonyl) glucoside, kaempferol 3-*O*-α-(2,3-*di*-*O*-β-glucosyl) rhamnoside, kaempferol 3-*O*-α-(2-*O*-β-glucosyl) rhamnoside-7-*O*-β-(6-*O*-acetyl) glucoside, kaempferol 3-*O*-α-(2-*O*-β-glucosyl)-rhamnoside |  |  |
| Anthocyanins | *Crocus baytopiorum* B.Mathew | Perianth | delphinidin 3,7-*di*-*O*-β-glucoside, petunidin 3,7-*di*-*O*-β-glucoside |  |  |
| Flavonoids |  |  | quercetin 3-*O*-β-sophoroside, kaempferol 3-*O*-β-sophoroside, quercetin 3,4′-*di*-*O*-β-glucoside, kaempferol 3,4′-*di*-*O*-β-glucoside, isorhamnetin 3,4′-*di*-*O*-β-glucoside |  |  |
| Anthocyanins | *Crocus kosaninii* Pulevic | Perianth | delphinidin 3,5-*di*-*O*-β-glucoside, petunidin 3,5-*di*-*O*-β-glucoside, delphinidin 3-*O*-β-rutinoside |  |  |
| Flavonoids |  |  | quercetin 3,4′-*di*-*O*-β-glucoside, kaempferol 3,4′-*di*-*O*-β-glucoside, isorhamnetin 3,4′-*di*-*O*-β-glucoside, myricetin 3-*O*-α-(2-*O*-β-glucosyl)-rhamnoside-7-*O*-β-glucoside, quercetin 3-*O*-α-(2-*O*-β-glucosyl)-rhamnoside-7-*O*-β-glucoside, kaempferol 3-*O*-α-(2-*O*-β-glucosyl)-rhamnoside-7-*O*-β-glucoside, kaempferol 3-*O*-α-(2-*O*-β-glucosyl)-rhamnoside-7-*O*-β-(6-*O*-malonyl) glucoside, kaempferol 3-*O*-α-(2,3-*di*-*O*-β-glucosyl) rhamnoside, kaempferol 3-*O*-α-(2-*O*-β-glucosyl) rhamnoside-7-*O*-β-(6-*O*-acetyl) glucoside, kaempferol 3-*O*-α-(2-*O*-β-glucosyl)-rhamnoside |  |  |
| Anthocyanins | *Crocus vernus* (L.) Hill |  | delphinidin 3,5-*di*-*O*-β-glucoside, petunidin 3,5-*di*-*O*-β-glucoside, selphinidin 3-*O*-β-rutinoside, petunidin 3-*O*-β-rutinoside |  |  |
|  |  | Perianth | delphinidin 3,5-*di*-*O*-β-glucoside, petunidin 3,5-*di*-*O*-β-glucoside, selphinidin 3-*O*-β-rutinoside, petunidin 3-*O*-β-rutinoside |  |  |
| Flavonoids |  |  | quercetin 3,4′-*di*-*O*-β-glucoside, kaempferol 3,4′-*di*-*O*-β-glucoside, isorhamnetin 3,4′-*di*-*O*-β-glucoside, myricetin 3-*O*-α-(2-*O*-β glucosyl)-rhamnoside-7-*O*-β-glucoside, quercetin 3-*O*-α-(2-*O*-β-glucosyl)-rhamnoside-7-*O*-β-glucoside, kaempferol 3-*O*-α-(2-*O*-β-glucosyl)-rhamnoside-7-*O*-β-glucoside, kaempferol 3-*O*-α-(2-*O*-β-glucosyl)-rhamnoside-7-*O*-β-(6-*O*-malonyl) glucoside, kaempferol 3-*O*-α-(2,3-*di*-*O*-β-glucosyl) rhamnoside, kaempferol 3-*O*-α-(2-*O*-β-glucosyl) rhamnoside-7-*O*-β-(6-*O*-acetyl) glucoside, kaempferol 3-*O*-α-(2-*O*-β-glucosyl)-rhamnoside |  |  |
|  |  | Perianth | quercetin 3,4′-*di*-*O*-β-glucoside, kaempferol 3,4′-*di*-*O*-β-glucoside, isorhamnetin 3,4′-*di*-*O*-β-glucoside, myricetin 3-*O*-α-(2-*O*-β-glucosyl)-rhamnoside-7-*O*-β-glucoside, quercetin 3-*O*-α-(2-*O*-β-glucosyl)-rhamnoside-7-*O*-β-glucoside, kaempferol 3-*O*-α-(2-*O*-β-glucosyl)-rhamnoside-7-*O*-β-glucoside, kaempferol 3-*O*-α-(2-*O*-β-glucosyl)-rhamnoside-7-*O*-β-(6-*O*-malonyl) glucoside, kaempferol 3-*O*-α-(2,3-*di*-*O*-β-glucosyl) rhamnoside, kaempferol 3-*O*-α-(2-*O*-β-glucosyl) rhamnoside-7-*O*-β-(6-*O*-acetyl) glucoside, kaempferol 3-*O*-α-(2-*O*-β-glucosyl)-rhamnoside |  |  |
| Anthocyanins | *Crocus tommasinianus* Herb. |  | delphinidin 3,5-*di*-*O*-β-glucoside, petunidin 3,5-*di*-*O*-β-glucoside, delphinidin 3-*O*-β-rutinoside |  |  |
| Flavonoids |  |  | quercetin 3,4′-*di*-*O*-β-glucoside, kaempferol 3,4′-*di*-*O*-β-glucoside, isorhamnetin 3,4′-*di*-*O*-β-glucoside, myricetin 3-*O*-α-(2-*O*-β-glucosyl)-rhamnoside-7-*O*-β-glucoside, Quercetin 3-*O*-α-(2-*O*-β-glucosyl)-rhamnoside-7-*O*-β-glucoside, kaempferol 3-*O*-α-(2-*O*-β-glucosyl)-rhamnoside-7-*O*-β-glucoside, kaempferol 3-*O*-α-(2-*O*-β-glucosyl)-rhamnoside-7-*O*-β-(6-*O*-malonyl) glucoside, kaempferol 3-*O*-α-(2,3-*di*-*O*-β-glucosyl) rhamnoside, kaempferol 3-*O*-α-(2-*O*-β-glucosyl) rhamnoside-7-*O*-β-(6-*O*-acetyl) glucoside, kaempferol 3-*O*-α-(2-*O*-β-glucosyl)-rhamnoside |  |  |
| Anthocyanins | *Crocus pelistericus* Pulevic | Perianth | delphinidin 3,5-*di*-*O*-β-glucoside, petunidin 3,5-*di*-*O*-β-glucoside, petunidin 3-*O*-β-rutinoside |  |  |
| Flavonoids |  |  | quercetin 3,4*'*-*di*-*O*-β-glucoside, kaempferol 3,4*'*-*di*-*O*-β-glucoside, isorhamnetin 3,4*'*-*di*-*O*-β-glucoside, myricetin 3-*O*-α-(2-*O*-β-glucosyl)-rhamnoside-7-*O*-β-glucoside, quercetin 3-*O*-α-(2-*O*-β-glucosyl)-rhamnoside-7-*O*-β-glucoside, kaempferol 3-*O*-α-(2-*O*-β-glucosyl)-rhamnoside-7-*O*-β-glucoside, kaempferol 3-*O*-α-(2-*O*-β-glucosyl)-rhamnoside-7-*O*-β-(6-*O*-malonyl) glucoside, kaempferol 3-*O*-α-(2,3-*di*-*O*-β-glucosyl) rhamnoside, kaempferol 3-*O*-α-(2-*O*-β-glucosyl) rhamnoside-7-*O*-β-(6-*O*-acetyl) glucoside, kaempferol 3-*O*-α-(2-*O*-β-glucosyl)-rhamnoside |  |  |
| Anthocyanins | *Crocus minimus* DC. |  | delphinidin 3,5-*di*-*O*-β-glucoside, petunidin 3,5-*di*-*O*-β-glucoside, delphinidin 3-*O*-β-rutinoside, petunidin 3-*O*-β-rutinoside |  |  |
| Flavonoids |  |  | myricetin 3-*O*-α-(2-*O*-β-glucosyl)-rhamnoside-7-*O*-β-glucoside, quercetin 3-*O*-α-(2-*O*-β-glucosyl)-rhamnoside-7-*O*-β-glucoside, kaempferol 3-*O*-α-(2-*O*-β-glucosyl)-rhamnoside-7-*O*-β-glucoside, kaempferol 3-*O*-α-(2-*O*-β-glucosyl)-rhamnoside-7-*O*-β-(6-*O*-malonyl) glucoside, kaempferol 3-*O*-α-(2,3-*di*-*O*-β-glucosyl) rhamnoside, kaempferol 3-*O*-α-(2-*O*-β-glucosyl) rhamnoside-7-*O*-β-(6-*O*-acetyl) glucoside, kaempferol 3-*O*-α-(2-*O*-β-glucosyl)-rhamnoside |  |  |
| Anthocyanins | *Crocus corsicus* Vanucchi |  | delphinidin 3,5-*di*-*O*-β-glucoside, petunidin 3,5-*di*-*O*-β-glucoside, delphinidin 3-*O*-β-rutinoside, petunidin 3-*O*-β-rutinoside |  |  |
| Flavonoids |  |  | myricetin 3-*O*-α-(2-*O*-β-glucosyl)-rhamnoside-7-*O*-β-glucoside, quercetin 3-*O*-α-(2-*O*-β-glucosyl)-rhamnoside-7-*O*-β-glucoside, kaempferol 3-*O*-α-(2-*O*-β-glucosyl)-rhamnoside-7-*O*-β-glucoside, kaempferol 3-*O*-α-(2-*O*-β-glucosyl)-rhamnoside-7-*O*-β-(6-*O*-malonyl) glucoside, kaempferol 3-*O*-α-(2,3-*di*-*O*-β-glucosyl) rhamnoside, kaempferol 3-*O*-α-(2-*O*-β-glucosyl) rhamnoside-7-*O*-β-(6-*O*-acetyl) glucoside, kaempferol 3-*O*-α-(2-*O*-β-glucosyl)-rhamnoside |  |  |
| Anthocyanins | *Crocus imperati* Ten. |  | delphinidin 3,5-*di*-*O*-β-glucoside, petunidin 3,5-*di*-*O*-β-glucoside, delphinidin 3-*O*-β-rutinoside, petunidin 3-*O*-β-rutinoside |  |  |
| Flavonoids |  |  | myricetin 3-*O*-α-(2-*O*-β-glucosyl)-rhamnoside-7-*O*-β-glucoside,Quercetin 3-*O*-α-(2-*O*-β-glucosyl)-rhamnoside-7-*O*-β-glucoside, kaempferol 3-*O*-α-(2-*O*-β-glucosyl)-rhamnoside-7-*O*-β-glucoside, kaempferol 3-*O*-α-(2-*O*-β-glucosyl)-rhamnoside-7-*O*-β-(6-*O*-malonyl)glucoside, kaempferol 3-*O*-α-(2,3-*di*-*O*-β-glucosyl) rhamnoside, kaempferol 3-*O*-α-(2-*O*-β-glucosyl) rhamnoside-7-*O*-β-(6-*O*-acetyl) glucoside, kaempferol 3-*O*-α-(2-*O*-β-glucosyl)-rhamnoside |  |  |
| Anthocyanins | *Crocus versicolor* Ker Gawl. | Perianth | delphinidin 3,5-*di*-*O*-β-glucoside, petunidin 3,5-*di*-*O*-β-glucoside, delphinidin 3-*O*-β-rutinoside, petunidin 3-*O*-β-rutinoside, delphinidin 3-*O*-β-glucoside-5-*O*-β-(6-*O*-malonyl) glucoside, petunidin 3,7-*di*-*O*-β-(6-*O*-malonyl) glucoside, malvidin 3,7-*di*-*O*-β-(6-*O*-malonyl) glucoside |  |  |
| Flavonoids |  |  | myricetin 3-*O*-α-(2-*O*-β-glucosyl)-rhamnoside-7-*O*-β-glucoside, quercetin 3-*O*-α-(2-*O*-β-glucosyl)-rhamnoside-7-*O*-β-glucoside, kaempferol 3-*O*-α-(2-*O*-β-glucosyl)-rhamnoside-7-*O*-β-glucoside, kaempferol 3-*O*-α-(2-*O*-β-glucosyl)-rhamnoside-7-*O*-β-(6-*O*-malonyl) glucoside, kaempferol 3-*O*-α-(2,3-*di*-*O*-β-glucosyl) rhamnoside, kaempferol 3-*O*-α-(2-*O*-β-glucosyl) rhamnoside-7-*O*-β-(6-*O*-acetyl) glucoside, kaempferol 3-*O*-α-(2-*O*-β-glucosyl)-rhamnoside |  |  |
| Anthocyanins | *Crocus longiflorus* Raf. |  | delphinidin 3,5-*di*-*O*-β-glucoside, petunidin 3,5-*di*-*O*-β-glucoside |  |  |
| Flavonoids |  |  | quercetin 3-*O*-β-sophoroside, kaempferol 3-*O*-β-sophoroside, quercetin 3,4*'*-*di*-*O*-β-glucoside, kaempferol 3,4*'*-*di*-*O*-β-glucoside, isorhamnetin 3,4*'*-*di*-*O*-β-glucoside, myricetin 3-*O*-α-(2-*O*-β-glucosyl)-rhamnoside-7-*O*-β-glucoside, quercetin 3-*O*-α-(2-*O*-β-glucosyl)-rhamnoside-7-*O*-β-glucoside, kaempferol 3-*O*-α-(2-*O*-β-glucosyl)-rhamnoside-7-*O*-β-glucoside, kaempferol 3-*O*-α-(2-*O*-β-glucosyl)-rhamnoside-7-*O*-β-(6-*O*-malonyl) glucoside, kaempferol 3-*O*-α-(2,3-*di*-*O*-β-glucosyl) rhamnoside, kaempferol 3-*O*-α-(2-*O*-β-glucosyl) rhamnoside-7-*O*-β-(6-*O*-acetyl) glucoside, kaempferol 3-*O*-α-(2-*O*-β-glucosyl)-rhamnoside |  |  |
| Anthocyanins | *Crocus niveus* Bowles |  | delphinidin 3,5-*di*-*O*-β-glucoside, petunidin 3,5-*di*-*O*-β-glucoside |  |  |
| Flavonoids |  |  | quercetin 3,4*'*-*di*-*O*-β-glucoside, kaempferol 3,4*'*-*di*-*O*-β-glucoside, isorhamnetin 3,4*'*-*di*-*O*-β-glucoside, myricetin 3-*O*-α-(2-*O*-β-glucosyl)-rhamnoside-7-*O*-β-glucoside, quercetin 3-*O*-α-(2-*O*-β-glucosyl)-rhamnoside-7-*O*-β-glucoside, kaempferol 3-*O*-α-(2-*O*-β-glucosyl)-rhamnoside-7-*O*-β-glucoside, kaempferol 3-*O*-α-(2-*O*-β-glucosyl)-rhamnoside-7-*O*-β-(6-*O*-malonyl) glucoside, kaempferol 3-*O*-α-(2,3-*di*-*O*-β-glucosyl) rhamnoside, kaempferol 3-*O*-α-(2-*O*-β-glucosyl) rhamnoside-7-*O*-β-(6-*O*-acetyl) glucoside, kaempferol 3-*O*-α-(2-*O*-β-glucosyl)-rhamnoside |  |  |
| Anthocyanins | *Crocus serotinus* subsp. *clusii* (J.Gay) B.Mathew | Perianth | delphinidin 3,5-*di*-*O*-β-glucoside, petunidin 3,5-*di*-*O*-β-glucoside |  |  |
| Flavonoids |  |  | quercetin 3-*O*-β-sophoroside, kaempferol 3-*O*-β-sophoroside, quercetin 3,4*'*-*di*-*O*-β-glucoside, kaempferol 3,4*'*-*di*-*O*-β-glucoside, isorhamnetin 3,4*'*-*di*-*O*-β-glucoside, myricetin 3-*O*-α-(2-*O*-β-glucosyl)-rhamnoside-7-*O*-β-glucoside, quercetin 3-*O*-α-(2-*O*-β-glucosyl)-rhamnoside-7-*O*-β-glucoside, kaempferol 3-*O*-α-(2-*O*-β-glucosyl)-rhamnoside-7-*O*-β-glucoside, kaempferol 3-*O*-α-(2-*O*-β-glucosyl)-rhamnoside-7-*O*-β-(6-*O*-malonyl) glucoside, kaempferol 3-*O*-α-(2,3-*di*-*O*-β-glucosyl) rhamnoside, kaempferol 3-*O*-α-(2-*O*-β-glucosyl) rhamnoside-7-*O*-β-(6-*O*-acetyl) glucoside, kaempferol 3-*O*-α-(2-*O*-β-glucosyl)-rhamnoside |  |  |
| Anthocyanins | *Crocus serotinus* subsp. *salzmannii* (J.Gay) B.Mathew |  | delphinidin 3,5-*di*-*O*-β-glucoside, petunidin 3,5-*di*-*O*-β-glucoside |  |  |
| Flavonoids |  |  | quercetin 3-*O*-β-sophoroside, kaempferol 3-*O*-β-sophoroside, quercetin 3,4*'*-*di*-*O*-β-glucoside, kaempferol 3,4*'*-*di*-*O*-β-glucoside, isorhamnetin 3,4*'*-*di*-*O*-β-glucoside, myricetin 3-*O*-α-(2-*O*-β-glucosyl)-rhamnoside-7-*O*-β-glucoside, quercetin 3-*O*-α-(2-*O*-β-glucosyl)-rhamnoside-7-*O*-β-glucoside, kaempferol 3-*O*-α-(2-*O*-β-glucosyl)-rhamnoside-7-*O*-β-glucoside, kaempferol 3-*O*-α-(2-*O*-β-glucosyl)-rhamnoside-7-*O*-β-(6-*O*-malonyl) glucoside, kaempferol 3-*O*-α-(2,3-*di*-*O*-β-glucosyl) rhamnoside, kaempferol 3-*O*-α-(2-*O*-β-glucosyl) rhamnoside-7-*O*-β-(6-*O*-acetyl) glucoside, kaempferol 3-*O*-α-(2-*O*-β-glucosyl)-rhamnoside |  |  |
| Anthocyanins | *Crocus nudiflorus* Sm. |  | delphinidin 3,5-*di*-*O*-β-glucoside, petunidin 3,5-*di*-*O*-β-glucoside, delphinidin 3-*O*-β-glucoside-5-*O*-β-(6-*O*-malonyl) glucoside |  |  |
|  |  | Perianth | delphinidin 3,5-*di*-*O*-β-glucoside, petunidin 3,5-*di*-*O*-β-glucoside, delphinidin 3-*O*-β-rutinoside, petunidin 3-*O*-β-rutinoside |  |  |
| Flavonoids |  |  | quercetin 3-*O*-β-sophoroside, kaempferol 3-*O*-β-sophoroside, myricetin 3-*O*-α-(2-*O*-β-glucosyl)-rhamnoside-7-*O*-β-glucoside, quercetin 3-*O*-α-(2-*O*-β-glucosyl)-rhamnoside-7-*O*-β-glucoside, kaempferol 3-*O*-α-(2-*O*-β-glucosyl)-rhamnoside-7-*O*-β-glucoside, kaempferol 3-*O*-α-(2-*O*-β-glucosyl)-rhamnoside-7-*O*-β-(6-*O*-malonyl) glucoside, kaempferol 3-*O*-α-(2,3-*di*-*O*-β-glucosyl) rhamnoside, kaempferol 3-*O*-α-(2-*O*-β-glucosyl) rhamnoside-7-*O*-β-(6-*O*-acetyl) glucoside, kaempferol 3-*O*-α-(2-*O*-β-glucosyl)-rhamnoside |  |  |
| Flavonoids |  |  | myricetin 3-*O*-α-(2-*O*-β-glucosyl)-rhamnoside-7-*O*-β-glucoside, quercetin 3-*O*-α-(2-*O*-β-glucosyl)-rhamnoside-7-*O*-β-glucoside, kaempferol 3-*O*-α-(2-*O*-β-glucosyl)-rhamnoside-7-*O*-β-glucoside, kaempferol 3-*O*-α-(2-*O*-β-glucosyl)-rhamnoside-7-*O*-β-(6-*O*-malonyl) glucoside, kaempferol 3-*O*-α-(2,3-*di*-*O*-β-glucosyl) rhamnoside, kaempferol 3-*O*-α-(2-*O*-β-glucosyl) rhamnoside-7-*O*-β-(6-*O*-acetyl) glucoside,  kaempferol 3-*O*-α-(2-*O*-β-glucosyl)-rhamnoside |  |  |
| Anthocyanins | *Crocus kotschyanus* K.Koch |  | delphinidin 3,5-*di*-*O*-β-glucoside |  |  |
| Flavonoids |  |  | quercetin 3-*O*-β-sophoroside, kaempferol 3-*O*-β-sophoroside, myricetin 3-*O*-α-(2-*O*-β-glucosyl)-rhamnoside-7-*O*-β-glucoside,  quercetin 3-*O*-α-(2-*O*-β-glucosyl)-rhamnoside-7-*O*-β-glucoside, kaempferol 3-*O*-α-(2-*O*-β-glucosyl)-rhamnoside-7-*O*-β-glucoside, kaempferol 3-*O*-α-(2-*O*-β-glucosyl)-rhamnoside-7-*O*-β-(6-*O*-malonyl) glucoside, kaempferol 3-*O*-α-(2,3-*di*-*O*-β-glucosyl) rhamnoside, kaempferol 3-*O*-α-(2-*O*-β-glucosyl) rhamnoside-7-*O*-β-(6-*O*-acetyl) glucoside, kaempferol 3-*O*-α-(2-*O*-β-glucosyl)-rhamnoside |  |  |
| Anthocyanins | *Crocus vallicola* Herb. |  | delphinidin 3,7-*di*-*O*-β-glucoside, petunidin 3,7-*di*-*O*-β-glucoside |  |  |
| Flavonoids |  |  | myricetin 3-*O*-α-(2-*O*-β-glucosyl)-rhamnoside-7-*O*-β-glucoside, quercetin 3-*O*-α-(2-*O*-β-glucosyl)-rhamnoside-7-*O*-β-glucoside, kaempferol 3-*O*-α-(2-*O*-β-glucosyl)-rhamnoside-7-*O*-β-glucoside |  |  |
| Anthocyanins | *Crocus mathewii* Kerndorff & Pasche | Perianth | delphinidin 3-*O*-β-rutinoside, petunidin 3-*O*-β-rutinoside, delphinidin 3-*O*-β-glucoside-5-*O*-β-(6-*O*-malonyl) glucoside, malvidin 3,7-*di*-*O*-β-(6-*O*-malonyl) glucoside |  |  |
| Flavonoids |  |  | kaempferol 3-*O*-β-(2-*O*-α-rhamnosyl)-glucoside, isorhamnetin 3-*O*-β-(2-*O*-α-rhamnosyl)-glucoside |  |  |
| Anthocyanins | *Crocus cartwrightianus* Herb. |  | delphinidin 3,5-*di*-*O*-β-glucoside, petunidin 3,5-*di*-*O*-β-glucoside, delphinidin 3-*O*-β-rutinoside, petunidin 3-*O*-β-rutinoside, petunidin 3,7-*di*-*O*-β-(6-*O*-malonyl) glucoside, malvidin 3,7-*di*-*O*-β-(6-*O*-malonyl) glucoside |  |  |
| Flavonoids |  |  | quercetin 3,4*'*-*di*-*O*-β-glucoside, kaempferol 3,4*'*-*di*-*O*-β-glucoside, isorhamnetin 3,4*'*-*di*-*O*-β-glucoside, myricetin 3-*O*-α-(2-*O*-β-glucosyl)-rhamnoside-7-*O*-β-glucoside, quercetin 3-*O*-α-(2-*O*-β-glucosyl)-rhamnoside-7-*O*-β-glucoside, kaempferol 3-*O*-α-(2-*O*-β-glucosyl)-rhamnoside-7-*O*-β-glucoside, kaempferol 3-*O*-α-(2-*O*-β-glucosyl)-rhamnoside-7-*O*-β-(6-*O*-malonyl) glucoside, kaempferol 3-*O*-α-(2,3-*di*-*O*-β-glucosyl) rhamnoside, kaempferol 3-*O*-α-(2-*O*-β-glucosyl) rhamnoside-7-*O*-β-(6-*O*-acetyl) glucoside, kaempferol 3-*O*-α-(2-*O*-β-glucosyl)-rhamnoside |  |  |
| Anthocyanins | *Crocus pallasii* Goldb. |  | delphinidin 3,5-*di*-*O*-β-glucoside, petunidin 3,5-*di*-*O*-β-glucoside, delphinidin 3-*O*-β-rutinoside, petunidin 3-*O*-β-rutinoside |  |  |
| Flavonoids |  |  | kaempferol 3-*O*-β-(2-*O*-α-rhamnosyl)-glucoside, isorhamnetin 3-*O*-β-(2-*O*-α-rhamnosyl)-glucoside, quercetin 3,4*'*-*di*-*O*-β-glucoside, kaempferol 3,4*'*-*di*-*O*-β-glucoside, Isorhamnetin 3,4*'*-*di*-*O*-β glucoside |  |  |
| Anthocyanins | *Crocus asumaniae* B.Mathew & T.Baytop | Perianth | delphinidin 3,5-*di*-*O*-β-glucoside, petunidin 3,5-*di*-*O*-β-glucoside, delphinidin 3-*O*-β-rutinoside |  |  |
| Flavonoids |  |  | myricetin 3-*O*-α-(2-*O*-β-glucosyl)-rhamnoside-7-*O*-β-glucoside, quercetin 3-*O*-α-(2-*O*-β-glucosyl)-rhamnoside-7-*O*-β-glucoside, kaempferol 3-*O*-α-(2-*O*-β-glucosyl)-rhamnoside-7-*O*-β-glucoside, kaempferol 3-*O*-α-(2-*O*-β-glucosyl)-rhamnoside-7-*O*-β-(6-*O*-malonyl) glucoside, kaempferol 3-*O*-α-(2,3-*di*-*O*-β-glucosyl) rhamnoside, kaempferol 3-*O*-α-(2-*O*-β-glucosyl) rhamnoside-7-*O*-β-(6-*O*-acetyl) glucoside, kaempferol 3-*O*-α-(2-*O*-β-glucosyl)-rhamnoside |  |  |
| Anthocyanins | *Crocus hadriaticus* Herb. |  | delphinidin 3,5-*di*-*O*-β-glucoside, petunidin 3,5-*di*-*O*-β-glucoside, delphinidin 3-*O*-β-rutinoside, petunidin 3-*O*-β-rutinoside |  |  |
| Flavonoids |  |  | myricetin 3-*O*-α-(2-*O*-β-glucosyl)-rhamnoside-7-*O*-β-glucoside, quercetin 3-*O*-α-(2-*O*-β-glucosyl)-rhamnoside-7-*O*-β-glucoside, kaempferol 3-*O*-α-(2-*O*-β-glucosyl)-rhamnoside-7-*O*-β-glucoside,  kaempferol 3-*O*-α-(2-*O*-β-glucosyl)-rhamnoside-7-*O*-β-(6-*O*-malonyl) glucoside, kaempferol 3-*O*-α-(2,3-*di*-*O*-β-glucosyl) rhamnoside, kaempferol 3-*O*-α-(2-*O*-β-glucosyl) rhamnoside-7-*O*-β-(6-*O*-acetyl) glucoside, kaempferol 3-*O*-α-(2-*O*-β-glucosyl)-rhamnoside |  |  |
| Anthocyanins | *Crocus oreocreticus* B.L.Burtt |  | delphinidin 3,5-*di*-*O*-β-glucoside, petunidin 3,5-*di*-*O*-β-glucoside, delphinidin 3-*O*-β-rutinoside, petunidin 3-*O*-β-rutinoside |  |  |
| Flavonoids |  |  | myricetin 3-*O*-α-(2-*O*-β-glucosyl)-rhamnoside-7-*O*-β-glucoside, quercetin 3-*O*-α-(2-*O*-β-glucosyl)-rhamnoside-7-*O*-β-glucoside, kaempferol 3-*O*-α-(2-*O*-β-glucosyl)-rhamnoside-7-*O*-β-glucoside,  kaempferol 3-*O*-α-(2-*O*-β-glucosyl)-rhamnoside-7-*O*-β-(6-*O*-malonyl) glucoside, kaempferol 3-*O*-α-(2,3-*di*-*O*-β-glucosyl) rhamnoside, kaempferol 3-*O*-α-(2-*O*-β-glucosyl) rhamnoside-7-*O*-β-(6-*O*-acetyl) glucoside, kaempferol 3-*O*-α-(2-*O*-β-glucosyl)-rhamnoside |  |  |
| Anthocyanins | *Crocus sativus* L. | Perianth | delphinidin 3,5-*di*-*O*-β-glucoside, petunidin 3,5-*di*-*O*-β-glucoside, delphinidin 3-*O*-β-rutinoside, petunidin 3-*O*-β-rutinoside, delphinidin 3-*O*-β-glucoside-5-*O*-β-(6-*O*-malonyl) glucoside, petunidin 3,7-*di*-*O*-β-(6-*O*-malonyl) glucoside, malvidin 3,7-*di*-*O*-β-(6-*O*-malonyl) glucoside |  |  |
| Flavonoids |  |  | quercetin 3-*O*-β-sophoroside, kaempferol 3-*O*-β-sophoroside, myricetin 3-*O*-α-(2-*O*-β-glucosyl)-rhamnoside-7-*O*-β-glucoside, quercetin 3-*O*-α-(2-*O*-β-glucosyl)-rhamnoside-7-*O*-β-glucoside, kaempferol 3-*O*-α-(2-*O*-β-glucosyl)-rhamnoside-7-*O*-β-glucoside, kaempferol 3-*O*-α-(2-*O*-β-glucosyl)-rhamnoside-7-*O*-β-(6-*O*-malonyl) glucoside, kaempferol 3-*O*-α-(2,3-*di*-*O*-β-glucosyl) rhamnoside, kaempferol 3-*O*-α-(2-*O*-β-glucosyl) rhamnoside-7-*O*-β-(6-*O*-acetyl) glucoside, kaempferol 3-*O*-α-(2-*O*-β-glucosyl)-rhamnoside |  |  |
| Anthocyanins | *Crocus ancyrensis* (Herb.) Maw |  | delphinidin 3-*O*-β-rutinoside |  |  |
| Flavonoids |  |  | kaempferol 3-*O*-β-(2-*O*-α-rhamnosyl)-glucoside, isorhamnetin 3-*O*-β-(2-*O*-α-rhamnosyl)-glucoside, quercetin 3,4*'*-*di*-*O*-β-glucoside, kaempferol 3,4*'*-*di*-*O*-β-glucoside, isorhamnetin 3,4*'*-*di*-*O*-β-glucoside, myricetin 3-*O*-α-(2-*O*-β-glucosyl)-rhamnoside-7-*O*-β-glucoside, quercetin 3-*O*-α-(2-*O*-β-glucosyl)-rhamnoside-7-*O*-β-glucoside, kaempferol 3-*O*-α-(2-*O*-β-glucosyl)-rhamnoside-7-*O*-β-glucoside, kaempferol 3-*O*-α-(2-*O*-β-glucosyl)-rhamnoside-7-*O*-β-(6-*O*-malonyl) glucoside, kaempferol 3-*O*-α-(2,3-*di*-*O*-β-glucosyl) rhamnoside, kaempferol 3-*O*-α-(2-*O*-β-glucosyl) rhamnoside-7-*O*-β-(6-*O*-acetyl) glucoside, kaempferol 3-*O*-α-(2-*O*-β-glucosyl)-rhamnoside |  |  |
| Anthocyanins | *Crocus cvijicii* Kosanin |  | petunidin 3-*O*-β-rutinoside |  |  |
| Flavonoids |  |  | quercetin 3,4*'*-*di*-*O*-β-glucoside, kaempferol 3,4*'*-*di*-*O*-β-glucoside, isorhamnetin 3,4*'*-*di*-*O*-β-glucoside, myricetin 3-*O*-α-(2-*O*-β-glucosyl)-rhamnoside-7-*O*-β-glucoside, quercetin 3-*O*-α-(2-*O*-β-glucosyl)-rhamnoside-7-*O*-β-glucoside, kaempferol 3-*O*-α-(2-*O*-β-glucosyl)-rhamnoside-7-*O*-β-glucoside, kaempferol 3-*O*-α-(2-*O*-β-glucosyl)-rhamnoside-7-*O*-β-(6-*O*-malonyl) glucoside, kaempferol 3-*O*-α-(2,3-*di*-*O*-β-glucosyl) rhamnoside, kaempferol 3-*O*-α-(2-*O*-β-glucosyl) rhamnoside-7-*O*-β-(6-*O*-acetyl) glucoside, kaempferol 3-*O*-α-(2-*O*-β-glucosyl)-rhamnoside |  |  |
| Anthocyanins | *Crocus hittiticus* T.Baytop & B.Mathew | Perianth | delphinidin 3-*O*-β-rutinoside, petunidin 3-*O*-β-rutinoside |  |  |
| Flavonoids |  |  | quercetin 3,4*'*-*di*-*O*-β-glucoside, kaempferol 3,4*'*-*di*-*O*-β-glucoside, isorhamnetin 3,4*'*-*di*-*O*-β-glucoside, myricetin 3-*O*-α-(2-*O*-β-glucosyl)-rhamnoside-7-*O*-β-glucoside, quercetin 3-*O*-α-(2-*O*-β-glucosyl)-rhamnoside-7-*O*-β-glucoside, kaempferol 3-*O*-α-(2-*O*-β-glucosyl)-rhamnoside-7-*O*-β-glucoside, kaempferol 3-*O*-α-(2-*O*-β-glucosyl)-rhamnoside-7-*O*-β-(6-*O*-malonyl) glucoside, kaempferol 3-*O*-α-(2,3-*di*-*O*-β-glucosyl) rhamnoside, kaempferol 3-*O*-α-(2-*O*-β-glucosyl) rhamnoside-7-*O*-β-(6-*O*-acetyl) glucoside, kaempferol 3-*O*-α-(2-*O*-β-glucosyl)-rhamnoside |  |  |
| Anthocyanins | *Crocus reticulatus* Steven ex Adam |  | delphinidin 3-*O*-β-rutinoside, petunidin 3-*O*-β-rutinoside |  |  |
| Flavonoids |  |  | quercetin 3,4*'*-*di*-*O*-β-glucoside, kaempferol 3,4*'*-*di*-*O*-β-glucoside, isorhamnetin 3,4*'*-*di*-*O*-β-glucoside, myricetin 3-*O*-α-(2-*O*-β-glucosyl)-rhamnoside-7-*O*-β-glucoside, quercetin 3-*O*-α-(2-*O*-β-glucosyl)-rhamnoside-7-*O*-β-glucoside, kaempferol 3-*O*-α-(2-*O*-β-glucosyl)-rhamnoside-7-*O*-β-glucoside, kaempferol 3-*O*-α-(2-*O*-β-glucosyl)-rhamnoside-7-*O*-β-(6-*O*-malonyl) glucoside, kaempferol 3-*O*-α-(2,3-*di*-*O*-β-glucosyl) rhamnoside, kaempferol 3-*O*-α-(2-*O*-β-glucosyl) rhamnoside-7-*O*-β-(6-*O*-acetyl) glucoside, kaempferol 3-*O*-α-(2-*O*-β-glucosyl)-rhamnoside |  |  |
| Anthocyanins | *Crocus gargaricus* Herb. |  | delphinidin 3,5-*di*-*O*-β-glucoside, petunidin 3,5-*di*-*O*-β-glucoside |  |  |
| Flavonoids |  |  | myricetin 3-*O*-α-(2-*O*-β-glucosyl)-rhamnoside-7-*O*-β-glucoside, quercetin 3-*O*-α-(2-*O*-β-glucosyl)-rhamnoside-7-*O*-β-glucoside, kaempferol 3-*O*-α-(2-*O*-β-glucosyl)-rhamnoside-7-*O*-β-glucoside, kaempferol 3-*O*-α-(2-*O*-β-glucosyl)-rhamnoside-7-*O*-β-(6-*O*-malonyl) glucoside, kaempferol 3-*O*-α-(2,3-*di*-*O*-β-glucosyl) rhamnoside, kaempferol 3-*O*-α-(2-*O*-β-glucosyl) rhamnoside-7-*O*-β-(6-*O*-acetyl),glucoside, kaempferol 3-*O*-α-(2-*O*-β-glucosyl)-rhamnoside |  |  |
| Anthocyanins | *Crocus robertianus* C.D.Brickell | Perianth | delphinidin 3,5-*di*-*O*-β-glucoside |  |  |
| Flavonoids |  |  | quercetin 3,4*'*-*di*-*O*-β-glucoside, kaempferol 3,4*'*-*di*-*O*-β-glucoside, isorhamnetin 3,4*'*-*di*-*O*-β-glucoside, quercetin 3-*O*-β-sophoroside, kaempferol 3-*O*-β-sophoroside |  |  |
| Anthocyanins | *Crocus nivalis* Bory & Chaub. |  | delphinidin 3,5-*di*-*O*-β-glucoside, petunidin 3,5-*di*-*O*-β-glucoside |  |  |
| Flavonoids |  |  | quercetin 3-*O*-β-sophoroside, kaempferol 3-*O*-β-sophoroside, myricetin 3-*O*-α-(2-*O*-β-glucosyl)-rhamnoside-7-*O*-β-glucoside, quercetin 3-*O*-α-(2-*O*-β-glucosyl)-rhamnoside-7-*O*-β-glucoside, kaempferol 3-*O*-α-(2-*O*-β-glucosyl)-rhamnoside-7-*O*-β-glucoside, kaempferol 3-*O*-α-(2-*O*-β-glucosyl)-rhamnoside-7-*O*-β-(6-*O*-malonyl) glucoside, kaempferol 3-*O*-α-(2,3-*di*-*O*-β-glucosyl) rhamnoside, kaempferol 3-*O*-α-(2-*O*-β-glucosyl) rhamnoside-7-*O*-β-(6-*O*-acetyl) glucoside, kaempferol 3-*O*-α-(2-*O*-β-glucosyl)-rhamnoside |  |  |
| Anthocyanins | *Crocus sieberi* J.Gay |  | delphinidin 3,5-*di*-*O*-β-glucoside, petunidin 3,5-*di*-*O*-β-glucoside |  |  |
| Flavonoids |  |  | quercetin 3-*O*-β-sophoroside, kaempferol 3-*O*-β-sophoroside, myricetin 3-*O*-α-(2-*O*-β-glucosyl)-rhamnoside-7-*O*-β-glucoside, quercetin 3-*O*-α-(2-*O*-β-glucosyl)-rhamnoside-7-*O*-β-glucoside, kaempferol 3-*O*-α-(2-*O*-β-glucosyl)-rhamnoside-7-*O*-β-glucoside, kaempferol 3-*O*-α-(2-*O*-β-glucosyl)-rhamnoside-7-*O*-β-(6-*O*-malonyl) glucoside, kaempferol 3-*O*-α-(2,3-*di*-*O*-β-glucosyl) rhamnoside, kaempferol 3-*O*-α-(2-*O*-β-glucosyl) rhamnoside-7-*O*-β-(6-*O*-acetyl) glucoside, kaempferol 3-*O*-α-(2-*O*-β-glucosyl)-rhamnoside |  |  |
| Anthocyanins | *Crocus sublimis* Herb. |  | delphinidin 3,5-*di*-*O*-β-glucoside, petunidin 3,5-*di*-*O*-β-glucoside |  |  |
| Flavonoids |  |  | quercetin 3-*O*-β-sophoroside, kaempferol 3-*O*-β-sophoroside, myricetin 3-*O*-α-(2-*O*-β-glucosyl)-rhamnoside-7-*O*-β-glucoside, quercetin 3-*O*-α-(2-*O*-β-glucosyl)-rhamnoside-7-*O*-β-glucoside, kaempferol 3-*O*-α-(2-*O*-β-glucosyl)-rhamnoside-7-*O*-β-glucoside, kaempferol 3-*O*-α-(2-*O*-β-glucosyl)-rhamnoside-7-*O*-β-(6-*O*-malonyl) glucoside, kaempferol 3-*O*-α-(2,3-*di*-*O*-β-glucosyl) rhamnoside, kaempferol 3-*O*-α-(2-*O*-β-glucosyl) rhamnoside-7-*O*-β-(6-*O*-acetyl) glucoside, kaempferol 3-*O*-α-(2-*O*-β-glucosyl)-rhamnoside |  |  |
| Anthocyanins | *Crocus veluchensis* Herb. | Perianth | delphinidin 3,7-*di*-*O*-β-glucoside, petunidin 3,7-*di*-*O*-β-glucoside, delphinidin 3,5-*di*-*O*-β-glucoside, petunidin 3,5-*di*-*O*-β-glucoside |  |  |
| Flavonoids |  |  | quercetin 3,4*'*-*di*-*O*-β-glucoside, kaempferol 3,4*'*-*di*-*O*-β-glucoside, isorhamnetin 3,4*'*-*di*-*O*-β-glucoside |  |  |
| Anthocyanins | *Crocus abantensis* T.Baytop & B.Mathew |  | petunidin 3,5-*di*-*O*-β-glucoside, delphinidin 3-*O*-β-rutinoside, petunidin 3-*O*-β-rutinoside |  |  |
| Flavonoids |  |  | quercetin 3-*O*-β-sophoroside, kaempferol 3-*O*-β-sophoroside, myricetin 3-*O*-α-(2-*O*-β-glucosyl)-rhamnoside-7-*O*-β-glucoside, quercetin 3-*O*-α-(2-*O*-β-glucosyl)-rhamnoside-7-*O*-β-glucoside, kaempferol 3-*O*-α-(2-*O*-β-glucosyl)-rhamnoside-7-*O*-β-glucoside, kaempferol 3-*O*-α-(2-*O*-β-glucosyl)-rhamnoside-7-*O*-β-(6-*O*-malonyl) glucoside, kaempferol 3-*O*-α-(2,3-*di*-*O*-β-glucosyl) rhamnoside, kaempferol 3-*O*-α-(2-*O*-β-glucosyl) rhamnoside-7-*O*-β-(6-*O*-acetyl) glucoside, kaempferol 3-*O*-α-(2-*O*-β-glucosyl)-rhamnoside |  |  |
| Anthocyanins | *Crocus angustifolius* Weston |  | delphinidin 3,5-*di*-*O*-β-glucoside, petunidin 3,5-*di*-*O*-β-glucoside, delphinidin 3-*O*-β-rutinoside, petunidin 3-*O*-β-rutinoside |  |  |
|  |  | Perianth | delphinidin 3,7-*di*-*O*-β-glucoside, petunidin 3,7-*di*-*O*-β-glucoside, delphinidin 3,5-*di*-*O*-β-glucoside, petunidin 3,5-*di*-*O*-β-glucoside, delphinidin 3-*O*-β-rutinoside, petunidin 3-*O*-β-rutinoside |  |  |
| Flavonoids |  |  | quercetin 3-*O*-β-sophoroside, kaempferol 3-*O*-β-sophoroside, myricetin 3-*O*-α-(2-*O*-β-glucosyl)-rhamnoside-7-*O*-β-glucoside, quercetin 3-*O*-α-(2-*O*-β-glucosyl)-rhamnoside-7-*O*-β-glucoside, kaempferol 3-*O*-α-(2-*O*-β-glucosyl)-rhamnoside-7-*O*-β-glucoside, kaempferol 3-*O*-α-(2-*O*-β-glucosyl)-rhamnoside-7-*O*-β-(6-*O*-malonyl) glucoside, kaempferol 3-*O*-α-(2,3-*di*-*O*-β-glucosyl) rhamnoside, kaempferol 3-*O*-α-(2-*O*-β-glucosyl) rhamnoside-7-*O*-β-(6-*O*-acetyl) glucoside, kaempferol 3-*O*-α-(2-*O*-β-glucosyl)-rhamnoside, quercetin 3,4*'*-*di*-*O*-β-glucoside, kaempferol 3,4*'*-*di*-*O*-β-glucoside, isorhamnetin 3,4*'*-*di*-*O*-β-glucoside |  |  |
| Flavonoids |  | Perianth | quercetin 3-*O*-β-sophoroside, kaempferol 3-*O*-β-sophoroside, myricetin 3-*O*-α-(2-*O*-β-glucosyl)-rhamnoside-7-*O*-β-glucoside, quercetin 3-*O*-α-(2-*O*-β-glucosyl)-rhamnoside-7-*O*-β-glucoside, kaempferol 3-*O*-α-(2-*O*-β-glucosyl)-rhamnoside-7-*O*-β-glucoside, kaempferol 3-*O*-α-(2-*O*-β-glucosyl)-rhamnoside-7-*O*-β-(6-*O*-malonyl) glucoside, kaempferol 3-*O*-α-(2,3-*di*-*O*-β-glucosyl) rhamnoside, kaempferol 3-*O*-α-(2-*O*-β-glucosyl) rhamnoside-7-*O*-β-(6-*O*-acetyl) glucoside, kaempferol 3-*O*-α-(2-*O*-β-glucosyl)-rhamnoside, quercetin 3,4*'*-*di*-*O*-β-glucoside, kaempferol 3,4*'*-*di*-*O*-β-glucoside, isorhamnetin 3,4*'*-*di*-*O*-β-glucoside |  |  |
| Anthocyanins | *Crocus cancellatus* Herb. |  | delphinidin 3,5-*di*-*O*-β-glucoside, petunidin 3,5-*di*-*O*-β-glucoside, delphinidin 3-*O*-β-rutinoside, petunidin 3-*O*-β-rutinoside |  |  |
| Flavonoids |  |  | myricetin 3-*O*-α-(2-*O*-β-glucosyl)-rhamnoside-7-*O*-β-glucoside, quercetin 3-*O*-α-(2-*O*-β-glucosyl)-rhamnoside-7-*O*-β-glucoside, kaempferol 3-*O*-α-(2-*O*-β-glucosyl)-rhamnoside-7-*O*-β-glucoside, kaempferol 3-*O*-α-(2-*O*-β-glucosyl)-rhamnoside-7-*O*-β-(6-*O*-malonyl) glucoside, kaempferol 3-*O*-α-(2,3-*di*-*O*-β-glucosyl) rhamnoside, kaempferol 3-*O*-α-(2-*O*-β-glucosyl) rhamnoside-7-*O*-β-(6-*O*-acetyl) glucoside, kaempferol 3-*O*-α-(2-*O*-β-glucosyl)-rhamnoside, quercetin 3,4*'*-*di*-*O*-β-glucoside, kaempferol 3,4*'*-*di*-*O*-β-glucoside, isorhamnetin 3,4*'*-*di*-*O*-β-glucoside |  |  |
| Anthocyanins | *Crocus cancellatus* subsp. *mazziaricus* (Herb.) B.Mathew |  | delphinidin 3,5-*di*-*O*-β-glucoside, petunidin 3,5-*di*-*O*-β-glucoside, delphinidin 3-*O*-β-rutinoside, petunidin 3-*O*-β-rutinoside |  |  |
| Flavonoids |  |  | myricetin 3-*O*-α-(2-*O*-β-glucosyl)-rhamnoside-7-*O*-β-glucoside, quercetin 3-*O*-α-(2-*O*-β-glucosyl)-rhamnoside-7-*O*-β-glucoside, kaempferol 3-*O*-α-(2-*O*-β-glucosyl)-rhamnoside-7-*O*-β-glucoside, kaempferol 3-*O*-α-(2-*O*-β-glucosyl)-rhamnoside-7-*O*-β-(6-*O*-malonyl) glucoside, kaempferol 3-*O*-α-(2,3-*di*-*O*-β-glucosyl) rhamnoside, kaempferol 3-*O*-α-(2-*O*-β-glucosyl) rhamnoside-7-*O*-β-(6-*O*-acetyl) glucoside, kaempferol 3-*O*-α-(2-*O*-β-glucosyl)-rhamnoside, quercetin 3,4*'*-*di*-*O*-β-glucoside, kaempferol 3,4*'*-*di*-*O*-β-glucoside, isorhamnetin 3,4*'*-*di*-*O*-β-glucoside |  |  |
| Anthocyanins | *Crocus atticus* (Boiss. & Orph.) Orph. | Perianth | petunidin 3,7-*di*-*O*-β-glucoside, delphinidin 3,5-*di*-*O*-β-glucoside, petunidin 3,5-*di*-*O*-β-glucoside, delphinidin 3-*O*-β-rutinoside, petunidin 3-*O*-β-rutinoside |  |  |
| Flavonoids |  |  | quercetin 3-*O*-β-sophoroside, kaempferol 3-*O*-β-sophoroside |  |  |
| Anthocyanins | *Crocus reticulatus* Steven ex Adam × *Crocus angustifolius* Weston |  | petunidin 3,5-*di*-*O*-β-glucoside, delphinidin 3-*O*-β-rutinoside, petunidin 3-*O*-β-rutinoside, delphinidin 3-*O*-β-glucoside-5-*O*-β-(6-*O*-malonyl) glucoside, petunidin 3,7-*di*-*O*-β-(6-*O*-malonyl) glucoside |  |  |
| Flavonoids |  |  | myricetin 3-*O*-α-(2-*O*-β-glucosyl)-rhamnoside-7-*O*-β-glucoside, quercetin 3-*O*-α-(2-*O*-β-glucosyl)-rhamnoside-7-*O*-β-glucoside, kaempferol 3-*O*-α-(2-*O*-β-glucosyl)-rhamnoside-7-*O*-β-glucoside, kaempferol 3-*O*-α-(2-*O*-β-glucosyl)-rhamnoside-7-*O*-β-(6-*O*-malonyl) glucoside, kaempferol 3-*O*-α-(2,3-*di*-*O*-β-glucosyl) rhamnoside, kaempferol 3-*O*-α-(2-*O*-β-glucosyl) rhamnoside-7-*O*-β-(6-*O*-acetyl) glucoside, kaempferol 3-*O*-α-(2-*O*-β-glucosyl)-rhamnoside, quercetin 3,4*'*-*di*-*O*-β-glucoside, kaempferol 3,4*'*-*di*-*O*-β-glucoside, isorhamnetin 3,4*'*-*di*-*O*-β-glucoside |  |  |
| Anthocyanins | *Crocus chrysanthus* (Herb.) Herb. |  | delphinidin 3-*O*-β-rutinoside, petunidin 3-*O*-β-rutinoside |  |  |
|  |  | Perianth | delphinidin 3-*O*-β-rutinoside, petunidin 3-*O*-β-rutinoside |  |  |
| Flavonoids |  |  | myricetin 3-*O*-α-(2-*O*-β-glucosyl)-rhamnoside-7-*O*-β-glucoside, quercetin 3-*O*-α-(2-*O*-β-glucosyl)-rhamnoside-7-*O*-β-glucoside, kaempferol 3-*O*-α-(2-*O*-β-glucosyl)-rhamnoside-7-*O*-β-glucoside, kaempferol 3-*O*-α-(2-*O*-β-glucosyl)-rhamnoside-7-*O*-β-(6-*O*-malonyl) glucoside, kaempferol 3-*O*-α-(2,3-*di*-*O*-β-glucosyl) rhamnoside, kaempferol 3-*O*-α-(2-*O*-β-glucosyl) rhamnoside-7-*O*-β-(6-*O*-acetyl) glucoside, kaempferol 3-*O*-α-(2-*O*-β-glucosyl)-rhamnoside, quercetin 3,4*'*-*di*-*O*-β-glucoside, kaempferol 3,4*'*-*di*-*O*-β-glucoside, isorhamnetin 3,4*'*-*di*-*O*-β-glucoside, kaempferol 3-*O*-β-(2-*O*-α-rhamnosyl)-glucoside, isorhamnetin 3-*O*-β-(2-*O*-α-rhamnosyl)-glucoside |  |  |
| Anthocyanins | *Crocus danfordiae* Maw |  | delphinidin 3-*O*-β-rutinoside, petunidin 3-*O*-β-rutinoside |  |  |
| Flavonoids |  |  | myricetin 3-*O*-α-(2-*O*-β-glucosyl)-rhamnoside-7-*O*-β-glucoside, quercetin 3-*O*-α-(2-*O*-β-glucosyl)-rhamnoside-7-*O*-β-glucoside, kaempferol 3-*O*-α-(2-*O*-β-glucosyl)-rhamnoside-7-*O*-β-glucoside, kaempferol 3-*O*-α-(2-*O*-β-glucosyl)-rhamnoside-7-*O*-β-(6-*O*-malonyl) glucoside, kaempferol 3-*O*-α-(2,3-*di*-*O*-β-glucosyl) rhamnoside, kaempferol 3-*O*-α-(2-*O*-β-glucosyl) rhamnoside-7-*O*-β-(6-*O*-acetyl) glucoside, kaempferol 3-*O*-α-(2-*O*-β-glucosyl)-rhamnoside |  |  |
| Anthocyanins | *Crocus melantherus* Boiss. & Orph. ex Maw |  | delphinidin 3-*O*-β-rutinoside, petunidin 3-*O*-β-rutinoside |  |  |
| Flavonoids |  |  | myricetin 3-*O*-α-(2-*O*-β-glucosyl)-rhamnoside-7-*O*-β-glucoside, quercetin 3-*O*-α-(2-*O*-β-glucosyl)-rhamnoside-7-*O*-β-glucoside, kaempferol 3-*O*-α-(2-*O*-β-glucosyl)-rhamnoside-7-*O*-β-glucoside, kaempferol 3-*O*-α-(2-*O*-β-glucosyl)-rhamnoside-7-*O*-β-(6-*O*-malonyl) glucoside, kaempferol 3-*O*-α-(2,3-*di*-*O*-β-glucosyl) rhamnoside, kaempferol 3-*O*-α-(2-*O*-β-glucosyl) rhamnoside-7-*O*-β-(6-*O*-acetyl) glucoside, kaempferol 3-*O*-α-(2-*O*-β-glucosyl)-rhamnoside, kaempferol 3-*O*-β-(2-*O*-α-rhamnosyl)-glucoside, isorhamnetin 3-*O*-β-(2-*O*-α-rhamnosyl)-glucoside |  |  |
| Anthocyanins | *Crocus biflorus* subsp. *stridii* (Papan. & Zacharof) B.Mathew |  | delphinidin 3-*O*-β-rutinoside, petunidin 3-*O*-β-rutinoside |  |  |
|  |  | Perianth | delphinidin 3-*O*-β-rutinoside, petunidin 3-*O*-β-rutinoside |  |  |
| Flavonoids |  |  | myricetin 3-*O*-α-(2-*O*-β-glucosyl)-rhamnoside-7-*O*-β-glucoside, quercetin 3-*O*-α-(2-*O*-β-glucosyl)-rhamnoside-7-*O*-β-glucoside, kaempferol 3-*O*-α-(2-*O*-β-glucosyl)-rhamnoside-7-*O*-β-glucoside, kaempferol 3-*O*-α-(2-*O*-β-glucosyl)-rhamnoside-7-*O*-β-(6-*O*-malonyl) glucoside, kaempferol 3-*O*-α-(2,3-*di*-*O*-β-glucosyl) rhamnoside, kaempferol 3-*O*-α-(2-*O*-β-glucosyl) rhamnoside-7-*O*-β-(6-*O*-acetyl) glucoside, kaempferol 3-*O*-α-(2-*O*-β-glucosyl)-rhamnoside |  |  |
|  |  | Perianth | myricetin 3-*O*-α-(2-*O*-β-glucosyl)-rhamnoside-7-*O*-β-glucoside, quercetin 3-*O*-α-(2-*O*-β-glucosyl)-rhamnoside-7-*O*-β-glucoside, kaempferol 3-*O*-α-(2-*O*-β-glucosyl)-rhamnoside-7-*O*-β-glucoside, kaempferol 3-*O*-α-(2-*O*-β-glucosyl)-rhamnoside-7-*O*-β-(6-*O*-malonyl) glucoside, kaempferol 3-*O*-α-(2,3-*di*-*O*-β-glucosyl) rhamnoside, kaempferol 3-*O*-α-(2-*O*-β-glucosyl) rhamnoside-7-*O*-β-(6-*O*-acetyl) glucoside, kaempferol 3-*O*-α-(2-*O*-β-glucosyl)-rhamnoside |  |  |
| Anthocyanins | *Crocus biflorus* subsp. *nubigena* (Herb.) B.Mathew |  | delphinidin 3-*O*-β-rutinoside, petunidin 3-*O*-β-rutinoside, delphinidin 3-*O*-β-glucoside-5-*O*-β-(6-*O*-malonyl) glucoside, petunidin 3,7-*di*-*O*-β-(6-*O*-malonyl) glucoside, malvidin 3,7-*di*-*O*-β-(6-*O*-malonyl) glucoside |  |  |
|  |  | Perianth | delphinidin 3-*O*-β-rutinoside, petunidin 3-*O*-β-rutinoside, delphinidin 3-*O*-β-glucoside-5-*O*-β-(6-*O*-malonyl) glucoside, petunidin 3,7-*di*-*O*-β-(6-*O*-malonyl) glucoside, malvidin 3,7-*di*-*O*-β-(6-*O*-malonyl) glucoside |  |  |
| Flavonoids |  |  | myricetin 3-*O*-α-(2-*O*-β-glucosyl)-rhamnoside-7-*O*-β-glucoside, quercetin 3-*O*-α-(2-*O*-β-glucosyl)-rhamnoside-7-*O*-β-glucoside, kaempferol 3-*O*-α-(2-*O*-β-glucosyl)-rhamnoside-7-*O*-β-glucoside, kaempferol 3-*O*-α-(2-*O*-β-glucosyl)-rhamnoside-7-*O*-β-(6-*O*-malonyl) glucoside, kaempferol 3-*O*-α-(2,3-*di*-*O*-β-glucosyl) rhamnoside, kaempferol 3-*O*-α-(2-*O*-β-glucosyl) rhamnoside-7-*O*-β-(6-*O*-acetyl) glucoside, kaempferol 3-*O*-α-(2-*O*-β-glucosyl)-rhamnoside |  |  |
|  |  | Perianth | myricetin 3-*O*-α-(2-*O*-β-glucosyl)-rhamnoside-7-*O*-β-glucoside, quercetin 3-*O*-α-(2-*O*-β-glucosyl)-rhamnoside-7-*O*-β-glucoside, kaempferol 3-*O*-α-(2-*O*-β-glucosyl)-rhamnoside-7-*O*-β-glucoside, kaempferol 3-*O*-α-(2-*O*-β-glucosyl)-rhamnoside-7-*O*-β-(6-*O*-malonyl),glucoside, kaempferol 3-*O*-α-(2,3-*di*-*O*-β-glucosyl) rhamnoside, kaempferol 3-*O*-α-(2-*O*-β-glucosyl) rhamnoside-7-*O*-β-(6-*O*-acetyl) glucoside, kaempferol 3-*O*-α-(2-*O*-β-glucosyl)-rhamnoside |  |  |
| Anthocyanins | *Crocus leichtlinii* (Dewer) Bowles | Perianth | delphinidin 3-*O*-β-rutinoside, petunidin 3-*O*-β-rutinoside, delphinidin 3-*O*-β-glucoside-5-*O*-β-(6-*O*-malonyl) glucoside, petunidin 3,7-*di*-*O*-β-(6-*O*-malonyl) glucoside, malvidin 3,7-*di*-*O*-β-(6-*O*-malonyl) glucoside |  |  |
| Flavonoids |  |  | quercetin 3,4*'*-*di*-*O*-β-glucoside, kaempferol 3,4*'*-*di*-*O*-β-glucoside, isorhamnetin 3,4*'*-*di*-*O*-β-glucoside |  |  |
| Anthocyanins | *Crocus adanensis* T.Baytop & B.Mathew |  | delphinidin 3,5-*di*-*O*-β-glucoside, petunidin 3,5-*di*-*O*-β-glucoside |  |  |
| Flavonoids |  |  | quercetin 3-*O*-β-sophoroside, kaempferol 3-*O*-β-sophoroside |  |  |
| Anthocyanins | *Crocus biflorus* subsp. *weldenii* (Hoppe & Fürnr.) K.Richt. |  | delphinidin 3,5-*di*-*O*-β-glucoside, petunidin 3,5-*di*-*O*-β-glucoside |  |  |
| Flavonoids |  |  | myricetin 3-*O*-α-(2-*O*-β-glucosyl)-rhamnoside-7-*O*-β-glucoside, quercetin 3-*O*-α-(2-*O*-β-glucosyl)-rhamnoside-7-*O*-β-glucoside, kaempferol 3-*O*-α-(2-*O*-β-glucosyl)-rhamnoside-7-*O*-β-glucoside, kaempferol 3-*O*-α-(2-*O*-β-glucosyl)-rhamnoside-7-*O*-β-(6-*O*-malonyl) glucoside, kaempferol 3-*O*-α-(2,3-*di*-*O*-β-glucosyl) rhamnoside, kaempferol 3-*O*-α-(2-*O*-β-glucosyl) rhamnoside-7-*O*-β-(6-*O*-acetyl) glucoside, kaempferol 3-*O*-α-(2-*O*-β-glucosyl)-rhamnoside |  |  |
| Anthocyanins | *Crocus aerius* Herb. |  | delphinidin 3,5-*di*-*O*-β-glucoside, petunidin 3,5-*di*-*O*-β-glucoside, delphinidin 3-*O*-β-rutinoside |  |  |
| Flavonoids |  |  | myricetin 3-*O*-α-(2-*O*-β-glucosyl)-rhamnoside-7-*O*-β-glucoside, quercetin 3-*O*-α-(2-*O*-β-glucosyl)-rhamnoside-7-*O*-β-glucoside, kaempferol 3-*O*-α-(2-*O*-β-glucosyl)-rhamnoside-7-*O*-β-glucoside, kaempferol 3-*O*-α-(2-*O*-β-glucosyl)-rhamnoside-7-*O*-β-(6-*O*-malonyl) glucoside, kaempferol 3-*O*-α-(2,3-*di*-*O*-β-glucosyl) rhamnoside, kaempferol 3-*O*-α-(2-*O*-β-glucosyl) rhamnoside-7-*O*-β-(6-*O*-acetyl) glucoside, kaempferol 3-*O*-α-(2-*O*-β-glucosyl)-rhamnoside |  |  |
| Anthocyanins | *Crocus biflorus* subsp. *tauri* (Maw) B.Mathew |  | delphinidin 3,7-*di*-*O*-β-glucoside, petunidin 3,7-*di*-*O*-β-glucoside, delphinidin 3,5-*di*-*O*-β-glucoside, petunidin 3,5-*di*-*O*-β-glucoside, delphinidin 3-*O*-β-rutinoside, petunidin 3-*O*-β-rutinoside |  |  |
| Flavonoids |  |  | kaempferol 3-*O*-β-(2-*O*-α-rhamnosyl)-glucoside, isorhamnetin 3-*O*-β-(2-*O*-α-rhamnosyl)-glucoside, quercetin 3,4*'*-*di*-*O*-β-glucoside, kaempferol 3,4*'*-*di*-*O*-β-glucoside, isorhamnetin 3,4*'*-*di*-*O*-β-glucoside |  |  |
| Anthocyanins | *Crocus biflorus* subsp. *crewei* (Hook.f.) B.Mathew | Perianth | petunidin 3,5-*di*-*O*-β-glucoside, delphinidin 3-*O*-β-rutinoside, petunidin 3-*O*-β-rutinoside |  |  |
| Flavonoids |  |  | kaempferol 3-*O*-β-(2-*O*-α-rhamnosyl)-glucoside, isorhamnetin 3-*O*-β-(2-*O*-α-rhamnosyl)-glucoside, quercetin 3,4*'*-*di*-*O*-β-glucoside, kaempferol 3,4*'*-*di*-*O*-β-glucoside, isorhamnetin 3,4*'*-*di*-*O*-β-glucoside |  |  |
| Anthocyanins | *Crocus biflorus* subsp. *adamii* (J.Gay) K.Richt. |  | petunidin 3,7-*di*-*O*-β-glucoside, delphinidin 3,5-*di*-*O*-β-glucoside, petunidin 3,5-*di*-*O*-β-glucoside, delphinidin 3-*O*-β-rutinoside, petunidin 3-*O*-β-rutinoside, delphinidin 3-*O*-β-glucoside-5-*O*-β-(6-*O*-malonyl) glucoside, malvidin 3,7-*di*-*O*-β-(6-*O*-malonyl) glucoside |  |  |
| Flavonoids |  |  | quercetin 3,4*'*-*di*-*O*-β-glucoside, kaempferol 3,4*'*-*di*-*O*-β-glucoside, isorhamnetin 3,4*'*-*di*-*O*-β-glucoside |  |  |
| Anthocyanins | *Crocus biflorus* subsp. *alexandri* (Nicic ex Velen.) B.Mathew |  | delphinidin 3,5-*di*-*O*-β-glucoside, petunidin 3,5-*di*-*O*-β-glucoside, delphinidin 3-*O*-β-rutinoside, petunidin 3-*O*-β-rutinoside, petunidin 3,7-*di*-*O*-β-(6-*O*-malonyl) glucoside, malvidin 3,7-*di*-*O*-β-(6-*O*-malonyl) glucoside |  |  |
| Flavonoids |  |  | quercetin 3,4*'*-*di*-*O*-β-glucoside, kaempferol 3,4*'*-*di*-*O*-β-glucoside, isorhamnetin 3,4*'*-*di*-*O*-β-glucoside, myricetin 3-*O*-α-(2-*O*-β-glucosyl)-rhamnoside-7-*O*-β-glucoside, quercetin 3-*O*-α-(2-*O*-β-glucosyl)-rhamnoside-7-*O*-β-glucoside, kaempferol 3-*O*-α-(2-*O*-β-glucosyl)-rhamnoside-7-*O*-β-glucoside, kaempferol 3-*O*-α-(2-*O*-β-glucosyl)-rhamnoside-7-*O*-β-(6-*O*-malonyl) glucoside, kaempferol 3-*O*-α-(2,3-*di*-*O*-β-glucosyl) rhamnoside, kaempferol 3-*O*-α-(2-*O*-β-glucosyl) rhamnoside-7-*O*-β-(6-*O*-acetyl) glucoside, kaempferol 3-*O*-α-(2-*O*-β-glucosyl)-rhamnoside |  |  |
| Anthocyanins | *Crocus biflorus* subsp. *isauricus* (Siehe ex Bowles) B.Mathew |  | delphinidin 3,5-*di*-*O*-β-glucoside, petunidin 3,5-*di*-*O*-β-glucoside, delphinidin 3-*O*-β-rutinoside, petunidin 3-*O*-β-rutinoside, delphinidin 3-*O*-β-glucoside-5-*O*-β-(6-*O*-malonyl) glucoside, petunidin 3,7-*di*-*O*-β-(6-*O*-malonyl) glucoside, malvidin 3,7-*di*-*O*-β-(6-*O*-malonyl) glucoside |  |  |
| Flavonoids |  |  | myricetin 3-*O*-α-(2-*O*-β-glucosyl)-rhamnoside-7-*O*-β-glucoside, quercetin 3-*O*-α-(2-*O*-β-glucosyl)-rhamnoside-7-*O*-β-glucoside, kaempferol 3-*O*-α-(2-*O*-β-glucosyl)-rhamnoside-7-*O*-β-glucoside, kaempferol 3-*O*-α-(2-*O*-β-glucosyl)-rhamnoside-7-*O*-β-(6-*O*-malonyl) glucoside, kaempferol 3-*O*-α-(2,3-*di*-*O*-β-glucosyl) rhamnoside, kaempferol 3-*O*-α-(2-*O*-β-glucosyl) rhamnoside-7-*O*-β-(6-*O*-acetyl) glucoside, kaempferol 3-*O*-α-(2-*O*-β-glucosyl)-rhamnoside |  |  |
| Anthocyanins | *Crocus biflorus* subsp. *pulchricolor* (Herb.) B.Mathew | Perianth | petunidin 3,5-*di*-*O*-β-glucoside, delphinidin 3-*O*-β-rutinoside, petunidin 3-*O*-β-rutinoside, delphinidin 3-*O*-β-glucoside-5-*O*-β-(6-*O*-malonyl) glucoside, petunidin 3,7-*di*-*O*-β-(6-*O*-malonyl) glucoside, malvidin 3,7-*di*-*O*-β-(6-*O*-malonyl) glucoside |  |  |
| Flavonoids |  |  | myricetin 3-*O*-α-(2-*O*-β-glucosyl)-rhamnoside-7-*O*-β-glucoside, quercetin 3-*O*-α-(2-*O*-β-glucosyl)-rhamnoside-7-*O*-β-glucoside, kaempferol 3-*O*-α-(2-*O*-β-glucosyl)-rhamnoside-7-*O*-β-glucoside, kaempferol 3-*O*-α-(2-*O*-β-glucosyl)-rhamnoside-7-*O*-β-(6-*O*-malonyl) glucoside, kaempferol 3-*O*-α-(2,3-*di*-*O*-β-glucosyl) rhamnoside, kaempferol 3-*O*-α-(2-*O*-β-glucosyl) rhamnoside-7-*O*-β-(6-*O*-acetyl) glucoside, kaempferol 3-*O*-α-(2-*O*-β-glucosyl)-rhamnoside |  |  |
| Anthocyanins | *Crocus korolkowii* Maw & Regel |  | delphinidin 3-*O*-β-rutinoside, petunidin 3-*O*-β-rutinoside |  |  |
| Flavonoids |  |  | kaempferol 3-*O*-β-(2-*O*-α-rhamnosyl)-glucoside, isorhamnetin 3-*O*-β-(2-*O*-α-rhamnosyl)-glucoside, quercetin 3-*O*-β-sophoroside, kaempferol 3-*O*-β-sophoroside |  |  |
| Anthocyanins | *Crocus alatavicus* Regel & Semen. |  | delphinidin 3-*O*-β-rutinoside, petunidin 3-*O*-β-rutinoside |  |  |
| Flavonoids |  |  | myricetin 3-*O*-α-(2-*O*-β-glucosyl)-rhamnoside-7-*O*-β-glucoside, quercetin 3-*O*-α-(2-*O*-β-glucosyl)-rhamnoside-7-*O*-β-glucoside, kaempferol 3-*O*-α-(2-*O*-β-glucosyl)-rhamnoside-7-*O*-β-glucoside, kaempferol 3-*O*-α-(2-*O*-β-glucosyl)-rhamnoside-7-*O*-β-(6-*O*-malonyl) glucoside, kaempferol 3-*O*-α-(2,3-*di*-*O*-β-glucosyl) rhamnoside, kaempferol 3-*O*-α-(2-*O*-β-glucosyl) rhamnoside-7-*O*-β-(6-*O*-acetyl) glucoside, kaempferol 3-*O*-α-(2-*O*-β-glucosyl)-rhamnoside |  |  |
| Anthocyanins | *Crocus flavus* Weston | Perianth | delphinidin 3-*O*-β-rutinoside |  |  |
| Flavonoids |  |  | myricetin 3-*O*-α-(2-*O*-β-glucosyl)-rhamnoside-7-*O*-β-glucoside, quercetin 3-*O*-α-(2-*O*-β-glucosyl)-rhamnoside-7-*O*-β-glucoside, kaempferol 3-*O*-α-(2-*O*-β-glucosyl)-rhamnoside-7-*O*-β-glucoside, kaempferol 3-*O*-α-(2-*O*-β-glucosyl)-rhamnoside-7-*O*-β-(6-*O*-malonyl) glucoside, kaempferol 3-*O*-α-(2,3-*di*-*O*-β-glucosyl) rhamnoside, kaempferol 3-*O*-α-(2-*O*-β-glucosyl) rhamnoside-7-*O*-β-(6-*O*-acetyl) glucoside, kaempferol 3-*O*-α-(2-*O*-β-glucosyl)-rhamnoside, kaempferol 3-*O*-β-(2-*O*-α-rhamnosyl)-glucoside, isorhamnetin 3-*O*-β-(2-*O*-α-rhamnosyl)-glucoside |  |  |
| Anthocyanins | *Crocus olivieri* J.Gay |  | delphinidin 3-*O*-β-rutinoside |  |  |
| Flavonoids |  |  | kaempferol 3-*O*-β-(2-*O*-α-rhamnosyl)-glucoside, isorhamnetin 3-*O*-β-(2-*O*-α-rhamnosyl)-glucoside, quercetin 3,4*'*-*di*-*O*-β-glucoside, kaempferol 3,4*'*-*di*-*O*-β-glucoside, isorhamnetin 3,4*'*-*di*-*O*-β-glucoside |  |  |
| Anthocyanins | *Crocus vitellinus* Wahlenb. |  | delphinidin 3-*O*-β-rutinoside, petunidin 3-*O*-β-rutinoside |  |  |
| Flavonoids |  |  | myricetin 3-*O*-α-(2-*O*-β-glucosyl)-rhamnoside-7-*O*-β-glucoside, quercetin 3-*O*-α-(2-*O*-β-glucosyl)-rhamnoside-7-*O*-β-glucoside, kaempferol 3-*O*-α-(2-*O*-β-glucosyl)-rhamnoside-7-*O*-β-glucoside, kaempferol 3-*O*-α-(2-*O*-β-glucosyl)-rhamnoside-7-*O*-β-(6-*O*-malonyl) glucoside, kaempferol 3-*O*-α-(2,3-*di*-*O*-β-glucosyl) rhamnoside, kaempferol 3-*O*-α-(2-*O*-β-glucosyl) rhamnoside-7-*O*-β-(6-*O*-acetyl) glucoside, kaempferol 3-*O*-α-(2-*O*-β-glucosyl)-rhamnoside, kaempferol 3-*O*-β-(2-*O*-α-rhamnosyl)-glucoside, isorhamnetin 3-*O*-β-(2-*O*-α-rhamnosyl)-glucoside |  |  |
| Anthocyanins | *Crocus candidus* E.D.Clarke | Perianth | delphinidin 3,5-*di*-*O*-β-glucoside, petunidin 3,5-*di*-*O*-β-glucoside, Delphinidin 3-*O*-β-rutinoside, petunidin 3-*O*-β-rutinoside |  |  |
| Flavonoids |  |  | quercetin 3,4*'*-*di*-*O*-β-glucoside, kaempferol 3,4*'*-*di*-*O*-β-glucoside, isorhamnetin 3,4*'*-*di*-*O*-β-glucoside |  |  |
| Anthocyanins | *Crocus graveolens* Boiss. & Reut. |  | delphinidin 3,5-*di*-*O*-β-glucoside, delphinidin 3-*O*-β-rutinoside, petunidin 3-*O*-β-rutinoside |  |  |
| Flavonoids |  |  | quercetin 3,4*'*-*di*-*O*-β-glucoside, kaempferol 3,4*'*-*di*-*O*-β-glucoside, isorhamnetin 3,4*'*-*di*-*O*-β-glucoside, myricetin 3-*O*-α-(2-*O*-β-glucosyl)-rhamnoside-7-*O*-β-glucoside, quercetin 3-*O*-α-(2-*O*-β-glucosyl)-rhamnoside-7-*O*-β-glucoside, kaempferol 3-*O*-α-(2-*O*-β-glucosyl)-rhamnoside-7-*O*-β-glucoside, kaempferol 3-*O*-α-(2-*O*-β-glucosyl)-rhamnoside-7-*O*-β-(6-*O*-malonyl) glucoside, kaempferol 3-*O*-α-(2,3-*di*-*O*-β-glucosyl) rhamnoside, kaempferol 3-*O*-α-(2-*O*-β-glucosyl) rhamnoside-7-*O*-β-(6-*O*-acetyl) glucoside, kaempferol 3-*O*-α-(2-*O*-β-glucosyl)-rhamnoside |  |  |
| Anthocyanins | *Crocus antalyensis* B.Mathew |  | delphinidin 3,7-*di*-*O*-β-glucoside, petunidin 3,7-*di*-*O*-β-glucoside, delphinidin 3,5-*di*-*O*-β-glucoside, petunidin 3,5-*di*-*O*-β-glucoside, delphinidin 3-*O*-β-rutinoside, petunidin 3-*O*-β-rutinoside, delphinidin 3-*O*-β-glucoside-5-*O*-β-(6-*O*-malonyl) glucoside, petunidin 3,7-*di*-*O*-β-(6-*O*-malonyl) glucoside, malvidin 3,7-*di*-*O*-β-(6-*O*-malonyl) glucoside |  |  |
| Flavonoids |  |  | quercetin 3,4*'*-*di*-*O*-β-glucoside, kaempferol 3,4*'*-*di*-*O*-β-glucoside, isorhamnetin 3,4*'*-*di*-*O*-β-glucoside, kaempferol 3-*O*-β-(2-*O*-α-rhamnosyl)-glucoside, isorhamnetin 3-*O*-β-(2-*O*-α-rhamnosyl)-glucoside, quercetin 3-*O*-β-sophoroside, kaempferol 3-*O*-β-sophoroside |  |  |
| Anthocyanins | *Crocus carpetanus* Boiss. & Reut. |  | delphinidin 3,5-*di*-*O*-β-glucoside, petunidin 3,5-*di*-*O*-β-glucoside, petunidin 3-*O*-β-rutinoside |  |  |
| Flavonoids |  |  | quercetin 3,4*'*-*di*-*O*-β-glucoside, kaempferol 3,4*'*-*di*-*O*-β-glucoside, isorhamnetin 3,4*'*-*di*-*O*-β-glucoside, kaempferol 3-*O*-β-(2-*O*-α-rhamnosyl)-glucoside, isorhamnetin 3-*O*-β-(2-*O*-α-rhamnosyl)-glucoside |  |  |
| Anthocyanins | *Crocus fleischeri* J.Gay |  | delphinidin 3-*O*-β-rutinoside, petunidin 3-*O*-β-rutinoside |  |  |
| Flavonoids |  |  | quercetin 3-*O*-β-sophoroside, kaempferol 3-*O*-β-sophoroside |  |  |
| Anthocyanins | *Crocus speciosus* M.Bieb. |  | delphinidin 3-*O*-β-rutinoside, petunidin 3-*O*-β-rutinoside |  |  |
|  |  | Perianth | delphinidin 3,5-*di*-*O*-β-glucoside, petunidin 3,5-*di*-*O*-β-glucoside, delphinidin 3-*O*-β-rutinoside, petunidin 3-*O*-β-rutinoside, delphinidin 3-*O*-β-glucoside-5-*O*-β-(6-*O*-malonyl) glucoside, petunidin 3,7-*di*-*O*-β-(6-*O*-malonyl) glucoside, malvidin 3,7-*di*-*O*-β-(6-*O*-malonyl) glucoside |  |  |
| Flavonoids |  |  | quercetin 3,4*'*-*di*-*O*-β-glucoside, kaempferol 3,4*'*-*di*-*O*-β-glucoside, isorhamnetin 3,4*'*-*di*-*O*-β-glucoside, myricetin 3-*O*-α-(2-*O*-β-glucosyl)-rhamnoside-7-*O*-β-glucoside, quercetin 3-*O*-α-(2-*O*-β-glucosyl)-rhamnoside-7-*O*-β-glucoside, kaempferol 3-*O*-α-(2-*O*-β-glucosyl)-rhamnoside-7-*O*-β-glucoside, kaempferol 3-*O*-α-(2-*O*-β-glucosyl)-rhamnoside-7-*O*-β-(6-*O*-malonyl) glucoside, kaempferol 3-*O*-α-(2,3-*di*-*O*-β-glucosyl) rhamnoside, kaempferol 3-*O*-α-(2-*O*-β-glucosyl) rhamnoside-7-*O*-β-(6-*O*-acetyl) glucoside, kaempferol 3-*O*-α-(2-*O*-β-glucosyl)-rhamnoside |  |  |
|  |  | Perianth | quercetin 3,4*'*-*di*-*O*-β-glucoside, kaempferol 3,4*'*-*di*-*O*-β-glucoside, isorhamnetin 3,4*'*-*di*-*O*-β-glucoside, myricetin 3-*O*-α-(2-*O*-β-glucosyl)-rhamnoside-7-*O*-β-glucoside, quercetin 3-*O*-α-(2-*O*-β-glucosyl)-rhamnoside-7-*O*-β-glucoside, kaempferol 3-*O*-α-(2-*O*-β-glucosyl)-rhamnoside-7-*O*-β-glucoside, kaempferol 3-*O*-α-(2-*O*-β-glucosyl)-rhamnoside-7-*O*-β-(6-*O*-malonyl) glucoside, kaempferol 3-*O*-α-(2,3-*di*-*O*-β-glucosyl) rhamnoside, kaempferol 3-*O*-α-(2-*O*-β-glucosyl) rhamnoside-7-*O*-β-(6-*O*-acetyl) glucoside, kaempferol 3-*O*-α-(2-*O*-β-glucosyl)-rhamnoside |  |  |
| Anthocyanins | *Crocus pulchellus* Herb. |  | petunidin 3,5-*di*-*O*-β-glucoside, delphinidin 3-*O*-β-rutinoside, petunidin 3-*O*-β-rutinoside |  |  |
|  |  | Perianth | delphinidin 3-*O*-β-rutinoside, petunidin 3-*O*-β-rutinoside |  |  |
| Flavonoids |  |  | quercetin 3,4*'*-*di*-*O*-β-glucoside, kaempferol 3,4*'*-*di*-*O*-β-glucoside, isorhamnetin 3,4*'*-*di*-*O*-β-glucoside, myricetin 3-*O*-α-(2-*O*-β-glucosyl)-rhamnoside-7-*O*-β-glucoside, quercetin 3-*O*-α-(2-*O*-β-glucosyl)-rhamnoside-7-*O*-β-glucoside, kaempferol 3-*O*-α-(2-*O*-β-glucosyl)-rhamnoside-7-*O*-β-glucoside, kaempferol 3-*O*-α-(2-*O*-β-glucosyl)-rhamnoside-7-*O*-β-(6-*O*-malonyl) glucoside, kaempferol 3-*O*-α-(2,3-*di*-*O*-β-glucosyl) rhamnoside, kaempferol 3-*O*-α-(2-*O*-β-glucosyl) rhamnoside-7-*O*-β-(6-*O*-acetyl) glucoside, kaempferol 3-*O*-α-(2-*O*-β-glucosyl)-rhamnoside |  |  |
|  |  | Perianth | quercetin 3,4*'*-*di*-*O*-β-glucoside, kaempferol 3,4*'*-*di*-*O*-β-glucoside, isorhamnetin 3,4*'*-*di*-*O*-β-glucoside, myricetin 3-*O*-α-(2-*O*-β-glucosyl)-rhamnoside-7-*O*-β-glucoside, quercetin 3-*O*-α-(2-*O*-β-glucosyl)-rhamnoside-7-*O*-β-glucoside, kaempferol 3-*O*-α-(2-*O*-β-glucosyl)-rhamnoside-7-*O*-β-glucoside, kaempferol 3-*O*-α-(2-*O*-β-glucosyl)-rhamnoside-7-*O*-β-(6-*O*-malonyl) glucoside, kaempferol 3-*O*-α-(2,3-*di*-*O*-β-glucosyl) rhamnoside, kaempferol 3-*O*-α-(2-*O*-β-glucosyl) rhamnoside-7-*O*-β-(6-*O*-acetyl) glucoside, kaempferol 3-*O*-α-(2-*O*-β-glucosyl)-rhamnoside |  |  |
| Anthocyanins | *Crocus laevigatus* Bory & Chaub. |  | delphinidin 3-*O*-β-rutinoside, petunidin 3-*O*-β-rutinoside |  |  |
| Flavonoids |  |  | quercetin 3,4*'*-*di*-*O*-β-glucoside, kaempferol 3,4*'*-*di*-*O*-β-glucoside, isorhamnetin 3,4*'*-*di*-*O*-β-glucoside |  |  |
| Anthocyanins | *Crocus banaticus* J.Gay |  | delphinidin 3,5-*di*-*O*-β-glucoside, petunidin 3,5-*di*-*O*-β-glucoside, delphinidin 3-*O*-β-rutinoside, petunidin 3-*O*-β-rutinoside |  |  |
| Flavonoids |  |  | myricetin 3-*O*-α-(2-*O*-β-glucosyl)-rhamnoside-7-*O*-β-glucoside, quercetin 3-*O*-α-(2-*O*-β-glucosyl)-rhamnoside-7-*O*-β-glucoside, kaempferol 3-*O*-α-(2-*O*-β-glucosyl)-rhamnoside-7-*O*-β-glucoside, kaempferol 3-*O*-α-(2-*O*-β-glucosyl)-rhamnoside-7-*O*-β-(6-*O*-malonyl) glucoside, kaempferol 3-*O*-α-(2,3-*di*-*O*-β-glucosyl) rhamnoside, kaempferol 3-*O*-α-(2-*O*-β-glucosyl) rhamnoside-7-*O*-β-(6-*O*-acetyl) glucoside, kaempferol 3-*O*-α-(2-*O*-β-glucosyl)-rhamnoside |  |  |
| Anthocyanins | *Crocus boryi* J.Gay | Perianth | Not reported |  |  |
| Flavonoids |  |  | quercetin 3,4*'*-*di*-*O*-β-glucoside, kaempferol 3,4*'*-*di*-*O*-β-glucoside, isorhamnetin 3,4*'*-*di*-*O*-β-glucoside |  |  |

| **Table 2.** Phytoconstituents of *Crocus* species detected by gas chromatography coupled to mass spectrometry (GC-MS) | | | | | |
| --- | --- | --- | --- | --- | --- |
| **Classifications** | **Parts** | **Species** | **Chemical component** | **Technique** | **References** |
| Lipids & Fatty Acid Methyl Ester | Leaves | *Crocus vallicola* Herb. | hexanal, 1-propoxy-2-propanol, methyl octanoate, methyl 8-oxooctanoate, methyl decanoate, methyl cyclopentaneundecanoate, methyl 9-oxononanoate, 1-(1-ethoxyethoxy) octane, methyl dodecanoate, dimethyl nonanedioate, methyl 7-hexadecenoate, 8,8-dimethoxy-2,6-dimethyl-2-octanol, methyl 8-(2-furyl)octanoate, methyl tetradecanoate, methyl pentadecanoate, methyl 9-hexadecenoate, methyl hexadecanoate, methyl 14-methylhexadecanoate, methyl heptadecanoate, methyl 2-hydroxy hexadecanoate, methyl octadecanoate, methyl 10-octadecenoate, methyl 16-methylheptadecanoate, 1,1-dimethoxy-9-octadecene, methyl (11*R*,12*R*,13*S*)-12,13-epoxy-11-methoxy-9-octadecenoate, 8-nitro-11-dodecanolide, methyl docosanoate, methyl tetracosanoate | GC-MS analysis | [58] |
| Lipids & Fatty Acid Methyl Ester | Roots |  | 1-propoxy-2-propanol, methyl 8-oxooctanoate, methyl 9-oxononanoate, methyl dodecanoate, methyl tetradecanoate, methyl pentadecanoate, 6,10,14-trimethyl-2-pentadecanone, methyl 7-hexadecenoate, methyl hexadecanoate, methyl heptadecanoate, methyl octadecanoate, methyl 10-octadecenoate, methyl 9-octadecenoate, methyl 3-octyloxiraneoctanoate, *N*-methyl-*N*-4,4-methoxy-1-hexahydropyridyl-2-butylacetamide, methyl eicosanoate, 2-dodecen-1-yl(-)succinic anhydride, methyl docosanoate, methyl tricosanoate, methyl tetracosanoate, methyl pentacosanoate |  |  |
| Volatile Carotenoid-Derived Components of Saffron | Flowers | *Crocus sativus* L. | 2,6,6-trimethyl-1,3-cyclohexadien-1-carboxyaldehyde (safranal); 3,5,5-trimethyl-2-cyclohexen-l-one (isophorone), 4-hydroxy-3,5,5-trimethyl-2-cyclohexen-l-one,  2,2,6-trimethylcyclohexane-1,4-dione (isomers), 2,6,6-trimethyl-2-cyclohexen-1,4-dione, 2-hydroxy-3,5,5-trimethyl-2-cyclohexen-l,4-dione,  4-hydroxy-2,6,6-trimethyl-1-cyclohexen-1-carboxaldehyde (hydroxy-β-cyclocitral, 4-β-hydroxysafranal), 4-hydroxy-2,6,6-trimethyl-3-oxo-1,4-cyclohexadien-1-carboxaldehyde, 2,3-epoxy-4-(hydroxymethylene)-3,5,5-trimethylcyclohexanone,  3-hydroxy-2,6,6-trimethyl-4-oxo-2-cyclohexen-1-carboxaldehyde, 2-methylene-6,6-dimethyl-3-cyclohexen-1-carboxaldehyde, 3,5,5-trimethyl-4-methylene-2-cyclohexen-l-one, 2,6,6-trimethyl-3-oxo-1,4-cyclohexadien-1-carboxyaldehyde,  2-hydroxy-4,4,6-trimethy 1-2,5-cyclohexadien-1-one, 2,4,6-trimethylbenzaldehyde, 4-hydroxy-2,6,6-trimethyl-3-oxo-1-cyclohexen-1-carboxaldehyde, 5,5-dimethyl-2-cyclohexene-1,4-dione, 3,5,5-trimethyl-3-cyclohexen-1-one, 2,6,6-trimethyl-3-oxo-1-cyclohexen-1-carboxaldehyde, 3,3-dimethyl-l-cyclohexene, 2,2-dimethyl-4-oxocyclohexan-1-carboxaldehyde, 2-hydroxy-3,5,5-trimethyl-4-methylene-2-cyclohexen-1-one, 2,4,6,6-tetramethyl-1-cyclohexene-1-carboxaldehyde, 2-hydroxy-3-methyl-5,6,7,8-tetrahydro-1,4-naphthochinon, 2,3-dihydro-l,4-naphthochinon, 3-(but-1-enyl)-2,4,4-trimethyl-2-cyclohexen-l-ol, 2,6,6-trimethyl-5-oxo-l,3-cyclohexadien-1-carboxaldehyde, methyl 2,6-dimethylbenzoate, 2,6,6-trimethyl-1,3-eyclohexadien-1-carboxylic acid, 2,2-dimethylcyclohexyl-1-carboxaldehyde, 1-(but-1-enyl)-2,6,6-trimethylcyclohexa-l,3-diene (isomers), 3-(but-l-enyl)-2,4,4-trimethylcyclohexan-l-ol, 5-(buta-1,3-dienyl)-4,4,6-trimethyl-1,5-cyclohexadien-1-ol, l,3,3-trimethyl-2-(3-oxobut-l-enyl)-1-cyclohexene (β-ionone), 2,4,4-trimethyl-3-(3-oxobut-l-enyl)-cyclohexan-l-ol (2 isomers), 3,7-dimethy1-1,6-octadiene, 2,6,6-trimethyl-1,4-cyclohexadien-1-carboxaldehyde, 2-hydroxy-5-cyclohexen-1,4-dione, 3,3,4,5-tetramethylcyclohexan-1-one, 4,6,6-trimethylbicyclo[3.1.1]hept-3-en-2-one, 4-hydroxy-2,6,6-trimethyl-3-oxocyclohexan-1-carboxaldehyde, 4-(2,2,6-trimethylcyclohexan-1-yl)-3-buten-2-one (dihydro-β-ionone), 2,4,4-trimethyl-3-(3-oxo-1-butenyl)-2-cyclohexen-l-ol, megastigma-7,9,13-triene (isomers), megastigma-4,6,8-triene (isomers), 2,6,6-trimethyl-4-oxo-2-cyclohexen-1-carboxaldehyde, 4-hydroxy-2,6,6-trimethyl-2-cyclohexen-1-one, 2-hydroxy-3,5,5-trimethyl-2-cyclohexen-l-one, 2,6,6-trimethyl-2,4-cycloheptadien-1-one, 5*-tert-*butyl-1,3-cyclopentadiene, geraniol, 6,10-dimethylundeca-5,9-dien-2-one (geranyl acetone),  2,6,6-trimethyl-2-cyclohexen-1-one, 4-hydroxymethy1-3,5,5-trimethyl-2 cyclohexen-l-one, 3,5,5-trimethyl-4-(3-hydroxy-1-butenyl)-1-cyclohexen-1-ol (3-hydroxy-α-ionone) | GC-MS analysis | [1,59-66] |
| Aroma compounds | Flowers | *Crocus sativus* L. | 2,3-butanedione, 4-hydroxy-2,5-dimethyl-3(2*H*)-furanone, 3,5,5-trimethyl-3-cyclohexen-1-one, linalool, 2-phenylethanol, 2,6,6-trimethyl-1,3-cyclohexadien-1-carboxaldehyde (safranal), 2-hydroxy-4,4,6-trimethyl-2,5-cyclohexadien-1-one, (*E*,*Z*)-2,6-nonadienal, (*E*,*E*)-2,4-decadienal, l-octen-3-one, 3-methylbutanoic acid, acetic acid, 2-acetyl-1-pyrroline, 3-(methylthio)-propanal | gas chromatography olfactometry (GCO) | [60,61] |
| Volatile oil | Styles | *Crocus sativus* L. | nonanal, 3,5,5-trimethyl-3-cyclohexen-1-one (β-isoforone), *n*-undecane, phenylethyl alcohol, 3,5,5-trimethyl-2-cyclohexen-1-one (isophorone), 2,6,6-trimethyl-2-cyclohexene-1,4-dione (4-ketoisophorone), 2,2,6-trimethyl-1,4-cyclohexanedione (dihydrooxophorone), 2,2-dimethyl-4-oxocyclohexan-1-carboxaldehyde, 2,6,6-trimethyl-1,3-cyclohexadiene-1-carboxaldehyde (safranal), *n*-dodecane, 4-methylene-3,5,5-trimethyl-2-cyclohexen-1-one (4-methylene-isophorone), 3,5,5-trimethyl-2-hydroxy-1,4-cyclohexadione-2-ene, isomer of 4-hydroxy-3,5,5-trimethyl-2-cyclohexen-1-one, decanol-1, 4-hydroxy-3,5,5-trimethyl-2-cyclohexen-1-one, 2,3-dihydroxy-1,4-naphthoquinone, 4-hydroxy-2,6,6-trimethyl-3-oxocyclohex-1-en-1-carboxaldehyde, 4-hydroxy-2,6,6-trimethyl-3-oxocyclohexa-1,4-diene-1-carboxaldehyde, 4-hydroxy-2,6,6-trimethyl-1-cyclohexene-1-carboxaldehyde (HTCC), 2-hydroxy-3,5,5-trimethylcyclohex-2-en-1,4-dione, 4-(2,6,6-trimethyl-1-cyclohexen-1yl)-3-buten-2-one (*trans*-β-ionone), 2-(hydroxyphenylmethyl)-cyclohexanone, palmitic acid, *n*-eicosane | GC-MS analysis | [67] |
| Aroma Compounds of Saffron | Stigmata | *Crocus sativus* L. | 5,5-dimethyl-2-cyclohexen-1,4-dione, 3,5,5-trimethyl-3-cyclohexen-1-one, 2-hydroxy-5-cyclohexen-1,4-dione, 2,6,6-trimethyl-1,4-cyclohexadien-1-carboxaldehyde, 3,7-dimethyl-1,6-octadiene, 3,5,5-trimethyl-2-cyclohexen-1-one (isophorone), 2,6,6-trimethyl-1,3-cyclohexadien-1-carboxaldehyde (safranal), 3,3,4,5-tetramethylcyclohexan-1-one, 2,6,6-trimethyl-2-cyclohexen-1,4-dione, 2,2-dimethyl-4-oxocyclohexan-1-carboxaldehyde, 2-hydroxy-4,4,6-trimethyl-2,5-cyclohexadien-1-one, 4-methylene-3,5,5-trimethyl-2-cyclohexen-1-one, 4,6,6-trimethylbicyclo[3.1.1]hept-3-en-2-one, 2,6,6-trimethylcyclohexan-1,4-dione, 2-phenylethanol, 4-(2,6,6,-trimethyl-1-cyclohexen-1-yl)-3-buten-2-one, 4-(2,2,6,-trimethyl-cyclohexan-1-yl)-3-buten-2-one, 4-hydroxy-3,5,5-trimethyl-2-cyclohexen-1-one, 2,6,6-trimethyl-3-oxo-1-cyclohexen-1-carboxaldehyde, 2,6,6-trimethyl-1,3-cyclohexadien-1-carboxylic acid, 4-hydroxy-2,6,6-trimethyl-3-oxo-1,4-cyclohexadien-1-carboxaldehyde, 2,4,4-trimethyl-3-(3-oxo-1-butenyl)-2-cyclohexen-1-ol, 4-hydroxy-2,6,6-trimethyl-3-oxo-cyclohexan-1-carboxaldehyde. | GC-MS analysis | [68] |
| Volatile oil | Styles | *Crocus laevigatus* Bory & Chaub. | nonanal, 3,5,5-trimethyl-3-cyclohexen-1-one (β-isoforone), *n*-undecane, phenylethyl alcohol, 3,5,5-trimethyl-2-cyclohexen-1-one (isophorone), 2,6,6-trimethyl-2-cyclohexene-1,4-dione (4-ketoisophorone), 2-hydroxy-3,5,5-trimethyl-cyclohex-2-enone, 2,2,6-trimethyl-1,4-cyclohexanedione (dihydrooxophorone), 2,2-dimethyl-4-oxocyclohexan-1-carboxaldehyde, 2,6,6-trimethyl-1,3-cyclohexadiene-1-carboxaldehyde (safranal), *n*-dodecane, 4-methylene-3,5,5-trimethyl-2-cyclohexen-1-one (4-methylene-isophorone), decanol-1, 4-hydroxy-3,5,5-trimethyl-2-cyclohexen-1-one, 2,3-dihydroxy-1,4-naphthoquinone, 4-hydroxy-2,6,6-trimethyl-3-oxocyclohexa-1,4-diene-1-carboxaldehyde, 4-hydroxy-2,6,6-trimethyl-1-cyclohexene-1-carboxaldehyde (HTCC), 2-hydroxy-3,5,5-trimethylcyclohex-2-en-1,4-dione, 4-(2,6,6-trimethyl-1-cyclohexen-1yl)-3-buten-2-one (*trans*-β-ionone), *n*-eicosane, *n*-octadecane, *n*-hexadecane, *n*-tetradecane, | GC-MS analysis | [67] |
| Volatile oil | Styles | *Crocus oreocreticus* B.L.Burtt | nonanal, 3,5,5-trimethyl-3-cyclohexen-1-one (β-isophorone), *n*-undecane, 3,5,5-trimethyl-2-cyclohexen-1-one (isophorone), 2,6,6-trimethyl-2-cyclohexene-1,4-dione (4-ketoisophorone), 2-hydroxy-3,5,5-trimethyl-cyclohex-2-enone, 2,2,6-trimethyl-1,4-cyclohexanedione (dihydrooxophorone), 2,2-dimethyl-4-oxocyclohexan-1-carboxaldehyde, 2,6,6-trimethyl-1,3-cyclohexadiene-1-carboxaldehyde (safranal), *n*-dodecane, 4-methylene-3,5,5-trimethyl-2-cyclohexen-1-one (4-methylene-isophorone), decanol-1, 4-hydroxy-3,5,5-trimethyl-2-cyclohexen-1-one, 4-hydroxy-2,6,6-trimethyl-3-oxocyclohexa-1,4-diene-1-carboxaldehyde, *n*-tetradecane, 4-hydroxy-2,6,6-trimethyl-1-cyclohexene-1-carboxaldehyde (HTCC), 2-hydroxy-3,5,5-trimethylcyclohex-2-ene-1,4-dione, 4-(2,6,6-trimethyl-1-cyclohexen-1yl)-3-buten-2-one (*trans*-β-ionone), *n*-hexadecane(n), *n*-octadecane, palmitic acid, *n*-eicosane | GC-MS analysis | [67] |
| Volatile oil | Styles | *Crocus cartwrightianus* Herb. | nonanal, 3,5,5-trimethyl-3-cyclohexen-1-one (β-isophorone), *n*-undecane, phenylethyl alcohol, 3,5,5-trimethyl-2-cyclohexen-1-one (isophorone), 2,6,6-trimethyl-2-cyclohexene-1,4-dione (4-ketoisophorone), 2,2,6-trimethyl-1,4-cyclohexanedione (dihydrooxophorone), 2,2-dimethyl-4-oxocyclohexan-1-carboxaldehyde, 2,6,6-trimethyl-1,3-cyclohexadiene-1-carboxaldehyde (safranal), *n*-dodecane, 4-methylene-3,5,5-trimethyl-2-cyclohexen-1-one (4-methylene-isophorone), 3,5,5-trimethyl-2-hydroxy-1,4-cyclohexadione-2-ene, decanol-1, 4-hydroxy-3,5,5-trimethyl-2-cyclohexen-1-one, 4-hydroxy-2,6,6-trimethyl-3-oxocyclohex-1-en-1-carboxaldehyde, 4-hydroxy-2,6,6-trimethyl-3-oxocyclohexa-1,4-diene-1-carboxaldehyde, *n*-tetradecane, 4-hydroxy-2,6,6-trimethyl-1-cyclohexene-1-carboxaldehyde (HTCC), 2-hydroxy-3,5,5-trimethylcyclohex-2-en-1,4-dione, 4-(2,6,6-trimethyl-1-cyclohexen-1-yl)-3-buten-2-one (*trans*-β-ionone), 4-(2,6,6-trimethyl-1,3-cyclohexadien-1-yl)-2-butanone (dihydrodehydro-α-ionone), *n*-hexadecane, *n*-octadecane | GC-MS analysis | [67] |
| Volatile oil | Stigmas | *Crocus cancellatus* subsp. *damascenus* (Herb.) B.Mathew | 2-furancarboxaldehyde, 5-methyl-, 4*H*-pyran-4-one-2,3-dihydro-3,5-dihydroxy-6-methyl-, 2-furancarboxaldehyde, 5-(hydroxymethyl)-, methyl palmitate, palmitic acid, xanthotoxin, methyl oleate, methyl stearate, osthol, isopimpinellin, methyl arachidate | GC-MS analysis | [69] |

.

References

1. Winterhalter, P.; Straubinger, M. Saffron-renewed interest in an ancient spice. *Food Reviews International* **2000**, *16*, 39-59, doi:10.1081/FRI-100100281.

2. Mathew, B. The Crocus, a Revision of the Genus Crocus (Iridaceae). *Portland: Timber Press* **1982**.

3. Peter, G.; Aaron, R.; Powell, M.P.; Davies, T.J.; Manning, J.C.; Bank, M.v.d.; Vincent, S. - Iridaceae ‘Out of Australasia’? Phylogeny, Biogeography, and Divergence Time Based on Plastid DNA Sequences. **2008**, *- 33*, - 508.

4. Petersen, G.; Seberg, O.; Thorsøe, S.; Jørgensen, T.; Mathew, B. A Phylogeny of the Genus Crocus (Iridaceae) Based on Sequence Data from Five Plastid Regions. *Taxon* **2008**, *57*, 487-499, doi:10.2307/25066017.

5. Harpke, D.; Meng, S.; Rutten, T.; Kerndorff, H.; Blattner, F.R. Phylogeny of Crocus (Iridaceae) based on one chloroplast and two nuclear loci: ancient hybridization and chromosome number evolution. *Molecular phylogenetics and evolution* **2013**, *66*, 617-627, doi:10.1016/j.ympev.2012.10.007.

6. Alsayied, N.F.; Fernández, J.A.; Schwarzacher, T.; Heslop-Harrison, J.S. Diversity and relationships of Crocus sativus and its relatives analysed by inter-retroelement amplified polymorphism (IRAP). *Annals of botany* **2015**, *116*, 359-368, doi:10.1093/aob/mcv103.

7. Grigg, D.B. *The Agricultural Systems of the World: An Evolutionary Approach*; Cambridge University Press: Cambridge, 1974; DOI: 10.1017/CBO9780511665882.

8. Jan, S.; Wani, A.; Kamili, A.; Kashtwari, M. Distribution, chemical composition and medicinal importance of saffron (*Crocus sativus* L.). *Afr J Plant Sci.* **2014**, *8*, 537-545, doi:10.5897/AJPS2014.1221.

9. Mathew, B. Crocus sativus and its allies (Iridaceae). *Plant Syst Evol* **1977**, *128*, 89-103.

10. Ault, A. On Food and Cooking. The Science and Lore of the Kitchen (McGee, Harold). *Journal of Chemical Education* **2003**, *80*, 880, doi:10.1021/ed080p880.1.

11. Negbi, M. Saffron Cultivation: Past, Present and Future Prospects. In *Saffron Crocus sativus L. Harwood Academy Publication, Amsterdam, Netherland*, Negbi, M., Ed. 1999; pp. 19-30.

12. Fernandez, J.A. Biology, biotechnology and biomedicine of saffron. *Recent research developments in plant science Vol* **2004**, *2*, 127-159.

13. Chen, S.A.; Zhao, B.; Wang, X.; Yuan, X.; Wang, Y. Promotion of the growth of Crocus sativus cells and the production of crocin by rare earth elements. *Biotechnology letters* **2004**, *26*, 27-30.

14. Molina, R.V.; Valero, M.; Navarro, Y.; Guardiola, J.L.; García-Luis, A. Temperature effects on flower formation in saffron (Crocus sativus L.). *Scientia Horticulturae* **2005**, *103*, 361-379, doi:<https://doi.org/10.1016/j.scienta.2004.06.005>.

15. Valijonovich, M.A. Creation of Plantation Crocus sativus L. in the Conditions of Uzbekistan. *Journal of Agricultural Science and Food Research* **2018**, *9*, 222-227.

16. Singla, R.K.; Bhat, V.G. Crocin: an overview. Indo Global Journal of Pharmaceutical Sciences. *Indo Global Journal of Pharmaceutical Sciences* **2011**, *1*, 281-286.

17. Lahmass, I.; Lamkami, T.; Delporte, C.; Sikdar, S.; Van Antwerpen, P.; Saalaoui, E.; Megalizzi, V. The waste of saffron crop, a cheap source of bioactive compounds. *Journal of Functional Foods* **2017**, *35*, 341-351, doi:<https://doi.org/10.1016/j.jff.2017.05.057>.

18. Bullitta, P.; Milia, M.; Pinna, M.E.; Satta, M.; Scarpa, G.M. Sowing density and crom size: two fundamental aspects of the cultivation of saffron. *Rivista Italiana EPPOS* **1996**, *19*, 139-145.

19. McGimpsey, J.A.; Douglas, M.H.; Wallace, A.R. Evaluation of saffron (Crocus sativus L.) production in New Zealand. *New Zealand Journal of Crop and Horticultural Science* **1997**, *25*, 159-168, doi:10.1080/01140671.1997.9514002.

20. Tammaro, F. Saffron (Crocus sativus L.) in Italy. In *Saffron: Crocus sativus L.*, Negbi, M., Ed. Harwood Academic Publishers: 1999; pp. 53-62.

21. Ghorbani, M. The Efficiency of saffron’s marketing channel in Iran. *World Applied Sciences Journal* **2008**.

22. Cusano, E.; Consonni, R.; Petrakis, E.A.; Astraka, K.; Cagliani, L.R.; Polissiou, M.G. Integrated analytical methodology to investigate bioactive compounds in Crocus sativus L. flowers. **2018**, *29*, 476-486, doi:10.1002/pca.2753.

23. Serrano-Diaz, J.; Sanchez, A.M.; Martinez-Tome, M.; Winterhalter, P.; Alonso, G.L. Flavonoid Determination in the Quality Control of Floral Bioresidues from Crocus sativus L. *Journal of agricultural and food chemistry* **2014**, *62*, 3125-3133, doi:10.1021/jf4057023.

24. Skrubis, B. The cultivation in Greece of Crocus sativus L. In *Proceedings of the international conference on saffron (Crocus sativus L.)*, Tammaro, F., Marra, L., Eds. L’Aquila: 1990; pp. 171-182.

25. Mzabri, I.; Legsayer, M.; Chetouani, M.; Aamar, A.; Kouddane, N.; Boukroute, A.; Bekkouch, I.; Berrichi, A. Saffron (*Crocus sativus* L.) yield parameter assessment of abiotic stressed corms stored in Low Temperature. *Journal of Materials and Environmental Science* **2017**, *8*, 3588-3597.

26. Gresta, F.; Lombardo, G.M.; Siracusa, L.; Ruberto, G. Saffron, an alternative crop for sustainable agricultural systems. A review. *Agronomy for Sustainable Development* **2008**, *28*, 95-112, doi:10.1051/agro:2007030.

27. Tammaro, F. Crocus sativus L. – cv. Piano di Navelli (L’Aquila saffron): environment, cultivation, morphometric characteristics, active principles, uses. In *Proceedings of the international conference on saffron (Crocus sativus L.)*, Tammaro, F., Marra, L., Eds. L’Aquila: 1990; pp. 47-57.

28. Koocheki, A.A. Indigenous knowledge in agriculture with particular reference to saffron production in Iran. *Acta Horticulturae* **2004**, *650*, 175-182.

29. Goliaris, A.H. Saffron cultivation in Greece. In *Saffron: Crocus sativus L.*, Negbi, M., Ed. Harwood Academic Publishers: 1999; pp. 73-85.

30. Dhar, A.K.; Sapru, R.; Rekha, K. Studies on saffron in Kashmir 1: Variation in natural population and its cytological behaviour. *Crop Improvement* **1988**, *15*, 52.

31. Hosseini, M.; Sadeghiand, B.; S.A., A. Influence of foliarfertilization on yield of saffron (*Crocus sativus* L.). *Acta Horticulturae* **2004**, *650*, 207-209.

32. Mylyaeva, E.L.; Azizbekova, N.S. Cytophysiological changes in the course of the development of the stem apices of saffron crocus. *Soviet Plant Physiology* **1978**, *25*, 227-233.

33. Azizbekova, N.; Milyaeva, S.H.; Lobova, E.L.; Chailakhyan, N.K.H. Effects of giberellin and kinetin on formation of flower organs insaffron crocus. *Soviet Plant Physiology* **1978**, *25*, 471-476.

34. Madan, C.L.; Kapoor, B.M.; Gupta, U.S. Saffro. *Economic Botany* **1967**, *20*, 377-385.

35. Thakur, R.N.; Singh, C.; Koul, B.L. First report of corm rot in Crocus sativus L. *Indian Phytopathology* **1992**, *45*.

36. Wani, A. Studies on corm rot of saffron (Crocus sativus L). *Division of Plant Pathology, SKUAST-K, Srinagar (J&K), India, P108+XVII.* **2004**.

37. Kalha, C.S.; Gupta, V.; Gupta, D. First report of sclerotial rot of saffron caused by *Sclerotium rolfsii*. *Plant Disease* **2007**, *91*, 203.

38. Boerema, G.H.; van Kesteren, H.A. The underground attacks on Crocus and Colchicum by the rusts Uromyces croci and Uromyces colchici respectively. *European Journal of Plant Pathology* **1965**, *71*, 136-144.

39. Moraga, A.R.; Trapero-Mozos, A.; Gómez-Gómez, L.; Ahrazem, O. Intersimple sequence repeat markers for molecular characterization of Crocus cartwrightianus cv. albus. *Industrial Crops and Products* **2010**, *32*, 147-151, doi:<https://doi.org/10.1016/j.indcrop.2010.04.012>.

40. Zengin, G.; Aumeeruddy, M.Z.; Diuzheva, A.; Jeko, J.; Cziaky, Z.; Yildiztugay, A.; Yildiztugay, E.; Mahomoodally, M.F. A comprehensive appraisal on Crocus chrysanthus (Herb.) Herb. flower extracts with HPLC-MS/MS profiles, antioxidant and enzyme inhibitory properties. *Journal of pharmaceutical and biomedical analysis* **2019**, *164*, 581-589, doi:10.1016/j.jpba.2018.11.022.

41. Li, C.Y.; Wu, T.S. Constituents of the pollen of Crocus sativus L. and their tyrosinase inhibitory activity. *Chemical & pharmaceutical bulletin* **2002**, *50*, 1305-1309.

42. Castro-Diaz, N.; Salaun, B.; Perret, R.; Sierro, S.; Romero, J.F.; Fernandez, J.A.; Rubio-Moraga, A.; Romero, P. Saponins from the Spanish saffron Crocus sativus are efficient adjuvants for protein-based vaccines. *Vaccine* **2012**, *30*, 388-397, doi:10.1016/j.vaccine.2011.10.080.

43. Kabiri, M.; Rezadoost, H.; Ghassempour, A. A comparative quality study of saffron constituents through HPLC and HPTLC methods followed by isolation of crocins and picrocrocin. *LWT* **2017**, *84*, 1-9, doi:<https://doi.org/10.1016/j.lwt.2017.05.033>.

44. Baba, S.A.; Malik, A.H.; Wani, Z.A.; Mohiuddin, T.; Shah, Z.; Abbas, N.; Ashraf, N. Phytochemical analysis and antioxidant activity of different tissue types of Crocus sativus and oxidative stress alleviating potential of saffron extract in plants, bacteria, and yeast. *South African Journal of Botany* **2015**, *99*, 80-87, doi:<https://doi.org/10.1016/j.sajb.2015.03.194>.

45. Bathaie, S.Z.; Mousavi, S.Z. New applications and mechanisms of action of saffron and its important ingredients. *Critical reviews in food science and nutrition* **2010**, *50*, 761-786, doi:10.1080/10408390902773003.

46. Maggi, L.; Sánchez, A.M.; Carmona, M.; Kanakis, C.D.; Anastasaki, E.; Tarantilis, P.A.; Polissiou, M.G.; Alonso, G.L. Rapid determination of safranal in the quality control of saffron spice (Crocus sativus L.). *Food chemistry* **2011**, *127*, 369-373, doi:<https://doi.org/10.1016/j.foodchem.2011.01.028>.

47. Rahaiee, S.; Moini, S.; Hashemi, M.; Shojaosadati, S.A. Evaluation of antioxidant activities of bioactive compounds and various extracts obtained from saffron (Crocus sativus L.): a review. *Journal of food science and technology* **2015**, *52*, 1881-1888, doi:10.1007/s13197-013-1238-x.

48. Licón, C.C.; Carmona, M.; Rubio, R.; Molina, A.; Berruga, M.I. Preliminary study of saffron (Crocus sativus L. stigmas) color extraction in a dairy matrix. *Dyes and Pigments* **2012**, *92*, 1355-1360, doi:<https://doi.org/10.1016/j.dyepig.2011.09.022>.

49. Straubinger, M.; Jezussek, M.; Waibel, R.; Winterhalter, P. Novel Glycosidic Constituents from Saffron. *Journal of agricultural and food chemistry* **1997**, *45*, 1678-1681, doi:10.1021/jf960861k.

50. Termentzi, A.; Kokkalou, E. LC-DAD-MS (ESI+) analysis and antioxidant capacity of crocus sativus petal extracts. *Planta medica* **2008**, *74*, 573-581, doi:10.1055/s-2008-1074498.

51. Lozano, P.; Castellar, M.R.; Simancas, M.J.; Iborra, J.L. A quantitative high-performance liquid chromatographic method to analyse commercial saffron (Crocus sativus L.) products. *Journal of Chromatography A* **1999**, *830*, 477-483, doi:<https://doi.org/10.1016/S0021-9673(98)00938-8>.

52. Menghini, L.; Leporini, L.; Vecchiotti, G.; Locatelli, M.; Carradori, S.; Ferrante, C.; Zengin, G.; Recinella, L.; Chiavaroli, A.; Leone, S., et al. Crocus sativus L. stigmas and byproducts: Qualitative fingerprint, antioxidant potentials and enzyme inhibitory activities. *Food research international (Ottawa, Ont.)* **2018**, *109*, 91-98, doi:10.1016/j.foodres.2018.04.028.

53. Sola, I.; Stipanicev, M.; Vujcic, V.; Mitic, B.; Hudek, A.; Rusak, G. Comparative Analysis of Native Crocus Taxa as a Great Source of Flavonoids with High Antioxidant Activity. *Plant foods for human nutrition (Dordrecht, Netherlands)* **2018**, *73*, 189-195, doi:10.1007/s11130-018-0674-1.

54. Rubio-Moraga, Á.; Gerwig, G.J.; Castro-Díaz, N.; Jimeno, M.L.; Escribano, J.; Fernández, J.-A.; Kamerling, J.P. Triterpenoid saponins from corms of Crocus sativus: Localization, extraction and characterization. *Industrial Crops and Products* **2011**, *34*, 1401-1409, doi:<https://doi.org/10.1016/j.indcrop.2011.04.013>.

55. Straubinger, M.; Bau, B.; Eckstein, S.; Fink, M.; Winterhalter, P. Identification of Novel Glycosidic Aroma Precursors in Saffron (Crocus sativus L.). *Journal of agricultural and food chemistry* **1998**, *46*, 3238-3243, doi:10.1021/jf980119f.

56. Wang, Y.; Han, T.; Zhang, X.G.; Zheng, C.J.; Rahman, K.; Qin, L.P. LC Fingerprint and Hierarchical Cluster Analysis of Crocus sativus L. from Different Locations in China. *Chromatographia* **2009**, *70*, 143-149.

57. Nørbæk, R.; Brandt, K.; Nielsen, J.K.; Ørgaard, M.; Jacobsen, N. Flower pigment composition of Crocus species and cultivars used for a chemotaxonomic investigation. *Biochemical Systematics and Ecology* **2002**, *30*, 763-791, doi:<https://doi.org/10.1016/S0305-1978(02)00020-0>.

58. Yayli, N.; Kiran, Z.; Seymen, H.; GenÇ, H. Characterization of Lipids and Fatty Acid Methyl Ester Contents in Leaves and Roots of Crocus vallicola. *Turkish Journal Chemistry* **2001**, *25*, 391- 395.

59. Tarantilis, P.A.; Tsoupras, G.; Polissiou, M. Determination of saffron (Crocus sativus L.) components in crude plant extract using high-performance liquid chromatography-UV-visible photodiode-array detection-mass spectrometry. *Journal of Chromatography A* **1995**, *699*, 107-118, doi:<https://doi.org/10.1016/0021-9673(95)00044-N>.

60. Cadwallader, K.; Baek, H.; Cai, M. Spices:  Flavor Chemistry and Antioxidant Properties. *Journal of the American Chemical Society* **1997**, *119*, 12423-12423, doi:10.1021/ja975606a.

61. Cadwallader, K.R. Flavor Chemistry of Saffron. In *Carotenoid-Derived Aroma Compounds*, American Chemical Society: 2001; Vol. 802, pp. 220-239.

62. Sujata, V.; Ravishankar, G.A.; Venkataraman, L.V. Methods for the analysis of the saffron metabolites crocin, crocetins, picrocrocin and safranal for the determination of the quality of the spice using thin-layer chromatography, high-performance liquid chromatography and gas chromatography. *Journal of Chromatography A* **1992**, *624*, 497-502, doi:<https://doi.org/10.1016/0021-9673(92)85699-T>.

63. Tarantilis, P.A.; Polissiou, M.; Manfait, M. Separation of picrocrocin, cis-trans-crocins and safranal of saffron using high-performance liquid chromatography with photodiode-array detection. *Journal of chromatography. A* **1994**, *664*, 55-61, doi:10.1016/0021-9673(94)80628-4.

64. Rödel, W.; Petrzika, M. Analysis of the volatile components of saffron. *Journal of High Resolution Chromatography* **1991**, *14*, 771-774, doi:10.1002/jhrc.1240141118.

65. Alonso, G.L.; Salinas, M.R.; Esteban-Infantes, F.J.; Sánchez-Fernández, M.A. Determination of Safranal from Saffron (Crocus sativus L.) by Thermal Desorption−Gas Chromatography. *Journal of agricultural and food chemistry* **1996**, *44*, 185-188, doi:10.1021/jf940665i.

66. Raina, B.L.; Agarwal, S.G.; Bhatia, A.K.; Gaur, G.S. Changes in pigments and volatiles of saffron (Crocus sativus L.) during processing and storage. *Journal of the Science of Food and Agriculture* **1996**, *71*, 32.

67. Lamari, F.N.; Papasotiropoulos, V.; Tsiris, D.; Bariamis, S.E.; Sotirakis, K.; Pitsi, E.; Vogiatzoglou, A.P.; Iatrou, G. Phytochemical and genetic characterization of styles of wild Crocus species from the island of Crete, Greece and comparison to those of cultivated C. sativus. *Fitoterapia* **2018**, *130*, 225-233, doi:10.1016/j.fitote.2018.09.003.

68. Tarantilis, P.A.; Polissiou, M.G. Isolation and Identification of the Aroma Components from Saffron (Crocus sativus). *Journal of agricultural and food chemistry* **1997**, *45*, 459-462, doi:10.1021/jf960105e.

69. Loizzo, M.R.; Marrelli, M.; Pugliese, A.; Conforti, F.; Nadjafi, F.; Menichini, F.; Tundis, R. Crocus cancellatus subsp. damascenus stigmas: chemical profile, and inhibition of alpha-amylase, alpha-glucosidase and lipase, key enzymes related to type 2 diabetes and obesity. *Journal of enzyme inhibition and medicinal chemistry* **2016**, *31*, 212-218, doi:10.3109/14756366.2015.1016510.
